# International Systematic Review of Utility Values Associated with Cardiovascular Disease and Reflections on Selecting Evidence for a UK Decision-Analytic Model

**DOI:** 10.1177/0272989X231214782

**Published:** 2024-01-04

**Authors:** Rob Hainsworth, Alexander J. Thompson, Bruce Guthrie, Katherine Payne, Gabriel Rogers

**Affiliations:** Manchester Centre for Health Economics, The University of Manchester, Manchester, UK; Manchester Centre for Health Economics, The University of Manchester, Manchester, UK; Advanced Care Research Centre, Centre for Population Health Sciences, Usher Institute, University of Edinburgh, Edinburgh, UK; Manchester Centre for Health Economics, The University of Manchester, Manchester, UK; Manchester Centre for Health Economics, The University of Manchester, Manchester, UK

**Keywords:** systematic review, utility values, cardiovascular disease, health-related quality of life, health economics, economic evaluation, cost-utility analysis, decision-analytic modelling, cost-effectiveness analysis

## Abstract

**Purpose:**

Evaluating interventions for cardiovascular disease (CVD) requires estimates of its effect on utility. We aimed to 1) systematically review utility estimates for CVDs published since 2013 and 2) critically appraise UK-relevant estimates and calculate corresponding baseline utility multipliers.

**Methods:**

We searched MEDLINE and Embase (April 22, 2021) using CVD and utility terms. We screened results for primary studies reporting utility distributions for people with experience of heart failure, myocardial infarction, peripheral arterial disease, stable angina, stroke, transient ischemic attack, or unstable angina. We extracted characteristics from studies included. For UK estimates based on the EuroQoL 5-dimension (EQ-5D) measure, we assessed risk of bias and applicability to a decision-analytic model, pooled arms/time points as appropriate, and estimated baseline utility multipliers using predicted utility for age- and sex- matched populations without CVD. We sought utility sources from directly applicable studies with low risk of bias, prioritizing plausibility of severity ordering in our base-case model and highest population ascertainment in a sensitivity analysis.

**Results:**

Most of the 403 studies identified used EQ-5D (*n* = 217) and most assessed Organisation for Economic Co-operation and Development populations (*n* = 262), although measures and countries varied widely. UK studies using EQ-5D (*n* = 29) produced very heterogeneous baseline utility multipliers for each type of CVD, precluding meta-analysis and implying different possible severity orderings. We could find sources that provided a plausible ordering of utilities while adequately representing health states.

**Conclusions:**

We cataloged international CVD utility estimates and calculated UK-relevant baseline utility multipliers. Modelers should consider unreported sources of heterogeneity, such as population differences, when selecting utility evidence from reviews.

**Highlights:**

- Published systematic reviews have summarized estimates of utility associated with cardiovascular disease published up to 2013.
- We 1) reviewed utility estimates for 7 types of cardiovascular disease published since 2013, 2) critically appraised UK-relevant studies, and 3) estimated the effect of each cardiovascular disease on baseline utility.
- Our review 1) recommends a consistent and reliable set of baseline utility multipliers for 7 types of cardiovascular disease and 2) provides systematically identified reference information for researchers seeking utility evidence for their own context.

Cardiovascular diseases (CVDs) are a leading cause of death and ill-health worldwide,^1^ and they represent a large share of health care spending across countries.^2–4^ Funders should consider the cost-effectiveness of interventions for CVDs compared with other possible uses of their budget. Many governments use thresholds based on cost-utility (a measure of cost-effectiveness) to approximate the cost to a health system of one funding decision preventing other investment opportunities. In health care, a cost-utility analysis measures the benefits and harms of an intervention by adjusting life expectancy for expected utility (usually representing health-related quality of life).

Utility is most commonly measured indirectly. This involves 1) study participants describing their health state using a standardized system, then 2) the researcher valuing the description according to general population preferences (tariffs).^5^ Descriptive systems include the EuroQol 5-dimension descriptive system^6^ (EQ-5D), Health Utilities Index^7^ (HUI), and the 12- and 36-item versions of the Short Form Health Survey^8,9^ (SF-12 and SF-36). To generate a preference-based measure of utility from the SF-12 or SF-36, researchers must convert individual items to the SF-6D descriptive system^10^ or map scores for the health domains it uses to the EQ-5D.^11^ Tariffs for the EQ-5D are available for many countries,^12^ including the United Kingdom.^13^ In some studies, participants directly assign a utility value to their own health state or one described. Direct measurement methods include 1) EuroQol’s visual analog scale (EQ-VAS)^6^ and 2) choosing between options varying a) time spent in alternate scenarios (time tradeoff)^14^ or b) levels of risk of experiencing them (standard gamble).^5^ Comparisons have shown that direct methods produce higher utility values than indirect measures do.^15^ The EQ-VAS differs from other utility measures (and arguably is conceptually limited), because a score of 0 represents the worst health state imaginable rather than death.^6^

Health economists often base cost-utility analysis on decision-analytic models (hereafter “models”). These models simulate the health states of an eligible population, each with associated health-state utility values (HSUVs). Most European guidelines express a preference for HSUVs derived using indirect methods and valued according to national tariffs.^16^ Most commonly, they recommend the 3-level version^6^ of the EQ-5D (EQ-5D-3L). In England and Wales, the National Institute for Health and Care Excellence’s (NICE’s) reference case^17^ stipulates that utility estimates must be based on EQ-5D-3L health state descriptions valued using a UK tariff.^13^

The Professional Society for Health Economics and Outcomes Research (ISPOR) recommends that modelers use systematic reviews to identify HSUVs^18^ and have published guidelines for conducting and using evidence from such a review.^19^ These guidelines recommend accounting for uncertainty in HSUVs using probabilistic analysis. Other guidelines^20^ emphasize the importance of capturing the relative effect of a modeled disease on utility by comparing its HSUV with the utility of the relevant population at risk (the “baseline” population). To do this, modelers can adjust the utility of a baseline populations with a particular age, sex, and comorbidity composition using an additive or multiplicative effect (hereafter, “baseline utility multiplier” refers to a multiplicative effect). Evidence that age and gender have statistically significant effects on HSUVs for people experiencing myocardial infarction (MI), stroke, and angina^21^ also supports the use of baseline utility multipliers in economic models of CVD interventions. The baseline utility of a cohort reflects their age, sex, and comorbidity composition, so multiplying this by a scalar representing the effect of a health state on utility will produce different HSUVs according to these factors.

NICE recommends preventive treatment (statins) for people with a 10-y risk of CVD exceeding 10% on the basis of their own cost-utility model (NICE CG181, 2014).^22^ For each of the 7 CVDs considered, the model includes 1 health state for the first year experiencing the disease and another for all later years. A systematic review of clinical and cost-effectiveness evidence for statins^23^ partly informed the choice of HSUVs in the CG181 model. We aimed to 1) review utility estimates for the 7 CVDs considered in CG181 and 2) critically appraise utility evidence and calculate baseline utility multipliers to suit CG181 and other UK models.

## Methods

We conducted and reported an international review according to PRISMA guidelines,^24^ cataloging included studies. We summarized UK studies meeting the NICE reference case for economic evaluations and assessed risk of bias and applicability to yearly model states. For studies meeting the NICE reference case, we transformed estimates to HSUVs for the first year experiencing the disease and after and calculated baseline utility multipliers. We selected preferred baseline utility multipliers for each health state.

### Review

We searched for studies published between January 1, 2013, and April 22, 2021 (since the date of a previous review^25^ of utilities for angina, MI, and stroke) assessing utility for adults (aged ≥ 18 y) who had experienced 1 of 7 prespecified CVDs. These diseases were heart failure, MI, peripheral arterial disease (PAD), stable angina, stroke, transient ischemic attack (TIA), and unstable angina. We included studies surveying participants with experience of the diseases, members of the general public, or both. Although we were primarily interested in UK evidence based on the EQ-5D, we included all countries and recognized utility instruments in the search strategy so that we would have alternative sources if UK and EQ-5D estimates were not available for any health state. Recognized direct methods were the visual analog scale, time tradeoff, and standard gamble. Recognized indirect methods were validated descriptive systems (e.g., EQ-5D, SF-36, HUI) valued using published, preference-based tariffs.

We excluded studies that were unavailable in English, those that recruited an unrepresentative subtype of an included disease (for example, heart failure with preserved ejection fraction or severely disabling stroke), and those that did not report measures of central tendency and dispersion for utility. Because cost-utility models may consider common comorbidities, we also included studies that reported utility for CVD in people experiencing type 2 diabetes mellitus and chronic kidney disease.

We searched MEDLINE and Embase. Appendix 1 outlines the search strategy, which combined search terms for utility with those for cardiovascular disease. We used the specificity-maximizing MEDLINE filter validated by Arber et al.^26^ to identify studies reporting utility and an Embase translation provided by the authors (personal communication, Julie Glanville, March 11, 2021; see Appendix 1). We took the search terms for cardiovascular disease from NICE CG181^22^ full guideline (see Appendix 1). Two reviewers screened electronically de-duplicated search results for retrieval, resolving conflicts by consensus. One reviewer assessed retrieved studies for eligibility. We extracted and tabulated summary information from all studies meeting our eligibility criteria.

### Full Data Extraction and Critical Appraisal (UK EQ-5D Only)

We extracted data from included studies that satisfied NICE’s requirements for utility evidence, namely, those that

1. used the EQ-5D to measure utility in the UK population or international population including the United Kingdom (we included the 3- and 5-level versions but categorized estimates based on the 5-level version as only partially applicable) and
2. valued health state descriptions according to the standard UK tariff.

We assessed the quality of the studies included in the analysis. We did not find published tools for this purpose, so we developed a bespoke quality appraisal tool using relevant guidance.^14,27–29^ Appendix 2 lists the criteria that we included in our quality appraisal tool, which comprised 1 set of criteria for applicability and another for risk of bias. We reached an overall judgment for each domain (directly applicable/partially applicable/not applicable; low/potentially serious/serious risk of bias) according to how likely the utility estimate would be to differ if unmet criteria for that domain were met (detail in Appendix 2). We excluded studies adjudged “not applicable” from further analysis. We reported common reasons for partial applicability and those for (potentially) serious risk of bias.

### Transforming Utilities to Apply to Common Model States (UK EQ-5D Only)

We transformed raw utilities from UK studies using the EQ-5D to HSUVs for the CG181 model. Appendix 4 shows the raw utility values and our transformations to suit modeled health states (as well as the baseline utility multipliers that we later calculated). The CG181 model had yearly cycles and separate states for the first year with a disease and all years after. For acute events, the first year represented the year of the event, whereas for chronic diseases it represented the year of diagnosis.

When studies provided estimates of utility at a single time point, we assigned the estimate to the health state for the first year if it was measured within 1 y of the cardiovascular event or diagnosis and the state for later years otherwise. We pooled baseline estimates across arms of randomized control trials (RCTs) that either

1. reported estimates of baseline utility only or
2. had longitudinal data but fewer than 30 participants in the control arm (we required a sample of 30 or more people to calculate standard errors using the central limit theorem^30^).

For other longitudinal studies, we used the area under the curve (AUC) approach with linear interpolation between reported time points to calculate either first-year utility, utility after the first year, or both. In RCTs, we used the arm best representing the untreated population unless we thought that arms equally represented standard of care, in which case we pooled them. For first-year states, we calculated AUC between 0 and 12 months. If estimates for 0 or 12 months were not available, we assumed that these were equal to the closest time point provided. For later-year states, we calculated AUC across all available time points that were 1 y or more after the event.

### Calculating Baseline Utility Multipliers from Transformed Utilities (UK EQ-5D Only)

Following ISPOR recommendations,^19^ we calculated baseline utility multipliers from the transformed HSUV estimates. To do this, we divided each HSUV estimate by an estimate of utility in a baseline population with the same age and sex characteristics (and comorbidities if appropriate) but without CVD. We used estimates of utility for non-CVD controls when studies provided them. Otherwise, we generated values using an age- and sex-adjusted model fitted to data from the Health Survey for England.^31^ To fit the model, we pooled responses from the 2003, 2006, and 2011 surveys, because these asked respondents whether a doctor had given them a diagnosis of CVD. Appendix 3 provides details of this model of baseline utility. We adjusted baseline utility for age and sex because they are commonly reported for clinical populations and often sufficiently capture variability in the determinants of health utility. For these reasons, many cost-utility models, including that underpinning CG181, stratify by these variables.

To characterize the uncertainty in each baseline utility multiplier estimate, we repeated the calculation described above for 10,000 samples of the corresponding mean utility. We took the samples of mean utility from scaled beta distributions fitted using the mean and standard error of each raw distribution (see Appendix 4). We scaled the beta distributions to be bound between −0.59 and 1 to reflect the possible range of EQ-5D index values.^13^ For estimates calculated by pooling or computing AUC, we sampled the mean of each required estimate from its raw distribution before transforming these into a single HSUV sample.

### Choosing Baseline Utility Multipliers for a Cost-Utility Model

We chose a set of candidate sources of utility evidence for our model from directly applicable studies with low risk of bias. If we could not find satisfactory UK sources for a health state, we sought candidates with a low risk of bias from studies in the wider review using EQ-5D in comparable European populations.

To choose the final set of baseline utility multipliers from the candidate sources, we prioritized the face validity of the estimated baseline utility multipliers relative to each other. We sourced multiple utilities from single studies when possible (especially for first and later years experiencing the same disease) to preserve their relationship in the study. Among studies that provided a plausible overall ordering, we preferred those that were likely to ascertain a representative proportion of the population with the disease (e.g., case series rather than interventional trials,^32^ studies with larger sample size). We consulted clinicians on the face validity of the final ordering of baseline utility multipliers.

### Applying the Baseline Utility Multipliers in a Probabilistic Cost-Utility Model

Modelers should apply the baseline utility multipliers in cost-utility models probabilistically to account for uncertainty in the effects of CVDs on utility. In each run of the probabilistic analysis, they can sample an HSUV for each health state by

1. sampling a value for the utility of the baseline population (with the same mean age and proportion of men but without CVD) using the model reported in Appendix 3,
2. sampling baseline utility multiplier values for each health state using the means and standard errors that we have provided in Appendix 4, and
3. multiplying the sampled baseline utility by the sampled baseline utility adjustments.

Modelers should use baseline utility for the baseline populations relevant to their decision problem, which may differ from ours. This may involve developing their own models to predict baseline utility that capture the causal determinants of health utility that decision makers consider.

## Results

### All Studies

**Figure 1:**
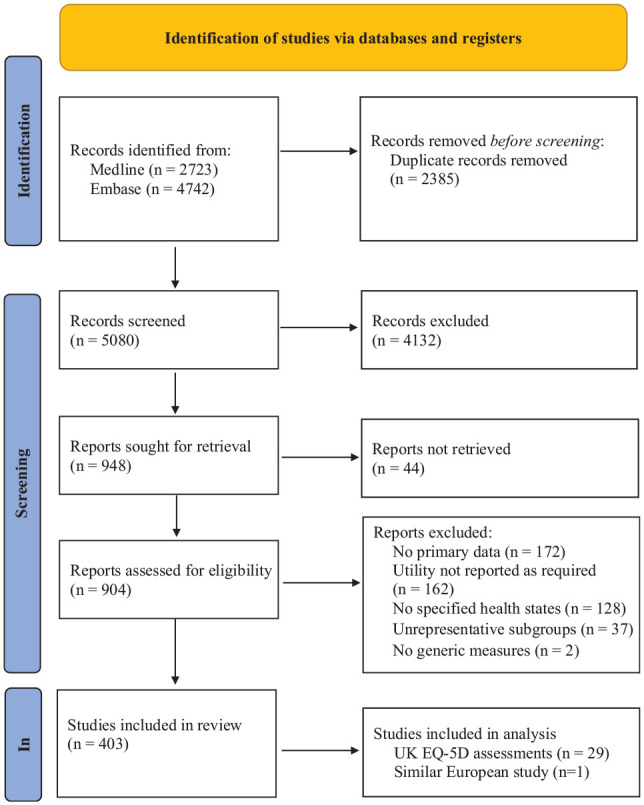
PRISMA flowchart.

Figure 1 illustrates the systematic review process, by which we identified 403 studies. Appendix 5 provides reference information, and Appendix 6 catalogs the following characteristics for the studies that we included: country, number of participants, utility instrument, and type(s) of CVD. Of the 403 studies included, 349 used an indirect method to elicit utility, and 54 used direct methods (50 used EQ-VAS, 3 used time tradeoff, and 1 used standard gamble). The descriptive systems used in the indirect methods were EQ-5D-3L (*n* = 181), SF-36 (*n* = 116), EQ-5D-5L (*n* = 36), 12-item Short Form Health Survey SF-12 (*n* = 5), HUI version 2 (*n* = 3), and HUI version 3 (*n* = 2). Six studies used other generic instruments.

We identified studies assessing populations experiencing the 7 CVDs including heart failure (*n* = 111), MI (*n* = 69), post-MI (*n* = 35), PAD (*n* = 45), stable angina (*n* = 34), stroke (*n* = 172), poststroke (*n* = 76), TIA (*n* = 14), post-TIA (*n* = 7), and unstable angina (*n* = 25). Forty-three studies assessed UK populations, 263 were based in other Organisation for Economic Cooperation and Development (OECD) countries and 97 in non-OECD countries. Twenty-two studies assessed populations with comorbid diabetes and 4 of those with chronic kidney disease.

### Identifying Studies Relevant to the NICE Reference Case

Table 1 summarizes the characteristics of 29 included studies reporting a utility value generated from the EQ-5D and collected (at least partly) in a UK setting.^17^ Two studies assessed populations with comorbid type 2 diabetes mellitus, and none assessed those with comorbid chronic kidney disease. Twenty-seven of the 29 studies analyzed reported raw utility associated with the diseases assessed, whereas 2 studies reported additive decrements from regression models.

**Table 1:** Characteristics of UK Studies Using the EQ-5D

Reference	Design	Number of Participants	Health State(s)	Time Point(s) Reported	Setting (Date)	Inclusion	Exclusion	Applicability and Risk of Bias (RoB)^a^
Agus et al.^33^ (2016)	RCT	345	Stable angina, post– stable angina	Year 1 area under the curve, 12 mo	2 Chest pain clinics in 1 Northern Irish Trust (not reported)	Symptoms of recent stable chest pain, no CVD or unstable angina	Heart/renal disease, body mass index >35, unable to use treadmill/receive imaging	Partially applicable (A1) Low RoB
Ali et al.^34^ (2017)	Case series	4,946	Stroke	3 mo	Registries and trials from 36 countries (not reported)	Complete modified Rankin scale and EQ-5D-3L at 3 mo	Acute registers with <100 records, not requiring standard diagnostic criteria	Partially applicable (A7) Potentially serious RoB (B6)
Alva et al.^35^ (2014)	RCT	352	MI, post-MI, stroke, heart failure with comorbid diabetes	Single	GPs in catchment areas of 23 hospitals (1997–2007)	Diabetes, ages 25–65 y, fasting plasma glucose >6 mmol/L recorded twice	Contraindications, past chronic illness, alternative indication	Directly applicable Low RoB
Ankolekar et al.^36^ (2014)	RCT	1,572	Stroke	Single	18 countries from 7 global regions. including United Kingdom (not reported)	Stroke within 48 h, high blood pressure, limb weakness	Treatment unsuitable, complicating diseases	Partially applicable (A7) Low RoB
Babber et al.^37^ (2020)	RCT	42	PAD	Single	Vascular clinic at a London hospital, 2014–2015	Nondiabetic people with IC of the legs and no tissue loss	ABPI ≥0.90, unable to follow protocol, implanted device, leg injury	Partially applicable (A1,6) Potentially serious RoB (B3)
Briggs et al.^38^ (2017)	RCT	16,480	MI, stroke, and heart failure with comorbid type 2 diabetes mellitus	Single	788 sites worldwide, including in United Kingdom (not reported)	T2DM, glycated hemoglobin: 6.5%–12.0%, history/risk of CVD	Incretin-based therapy, renal disease, creatinine level >6.0 mg/dL	Partially applicable (A7) Low RoB
Ezeofor et al.^39^ (2021)	RCT	19	PAD	0 and 3 mo from randomization	2 Hospitals in Greater Manchester, 2017–2019	Revascularized critical limb ischemia, grade 0–2 wound	Vascular or skin diseases, deep vein thrombosis, current or upcoming treatment	Partially applicable (A7,6) Potentially serious RoB (B1)
Ford et al.^40^ (2018)	RCT	151	Stable angina	0 and 2 mo from referral	2 cardiac centers covering West Scotland, 2016–2017	Coronary angiography to investigate angina	Reason for angiography noncoronary, unable to give informed consent	Partially applicable (A6) Low RoB
Forster et al.^41^ (2015)	Cluster RCT	800	Stroke, poststroke	0, 6, and 12 mo	Cluster-randomized stroke care coordinators (not reported)	Stroke within 6 wk, awaiting care coordinator	Care home residence, requires palliative care	Directly applicable, potentially serious RoB (B6)
Gallagher et al.^42^ (2019)	Cross-sectional	152	Heart failure	Single	2 London cardiology clinics, May 2015–2017	People with heart failure attending outpatient cardiology clinics	Not reported	Directly applicable Low RoB
Green et al.^43^ (2018)	RCT	30	PAD	Single	Tertiary vascular surgical unit (not reported)	PAD, ABPI <0.9, unilateral calf claudication, best medical care	Warfarin therapy, cancer, had unilateral thigh IC or bilateral IC	Partially applicable (A1) Potentially serious RoB (B1)
Hurdus et al.^44^ (2020)	RCT	2,612	MI, post-MI	0, 6, and 12 mo	48 NHS hospitals in England between (not reported)	Adults hospitalized with all types of acute MI	Terminal illness or other factors preventing follow-up	Directly applicable, Low RoB
Jenkinson et al.^45^ (2013)	Validation	151	Stroke	Single	19 diverse GPs, London and North West England (not reported)	Stroke survivors identified using Read codes	Severe illness or mental incapacity unrelated to stroke	Directly applicable, potentially serious RoB (B1)
Lewis et al.^46^ (2014)	RCT	2,382	MI	0 mo	10 countries including United Kingdom (not reported)	Adults, acute MI occurring 12 h to 10 d, heart failure	Other life-threatening/heart diseases, contraindications	Partially applicable (A7) Potentially serious RoB (B3)
Logan et al.^47^ (2014)	RCT	568	Poststroke	Single	GPs, outpatient and community care across GB, 2009–2011	Adults who had experienced a stroke > 6 wk previously	Unable to follow protocol; completing therapy or rehabilitation	Partially applicable (A1) Low RoB
Luengo-Fernandez et al.^48^ (2013)	Case series	1,188	Stroke, poststroke TIA, post-TIA, non-CV	1, 6, 12, 24, and 60 mo	9 Oxfordshire GPs, April 2002 – (not reported)	Suspected stroke or TIA	Temporary registration	Directly applicable Low RoB
McCreanor et al.^49^ (2021)	RCT	200	Stable angina	0 and 6 wk from randomization	4 trusts and 1 cardiac center, South England, 2014–2017	Age <85 y, angina or equivalent symptoms, suitable for PCI	ACS, hypertension, CABG, contraindications, life expectancy <2 y	Partially applicable (A6) Low RoB
Mejía et al.^50^ (2014)	RCT	260	Heart failure	Single	GPs, acute and specialist care, 2 regions, 2006–2008	Record of heart failure from hospital discharge or GP register	Cognitive disability, care home residency, life-threatening diseases	Directly applicable Low RoB
Monahan et al.^51^ (2017)	Validation	304	Heart failure	6 mo from recruitment	28 GPs in central England, May 2011– August 2013	Age >55 y, recent symptoms suggestive of heart failure	Previous ACS, alternative diagnosis, symptoms requiring management	Partially applicable (A1) Low RoB
Munyombwe et al.^52^ (2020)	Case series	9,332	MI, post-MI	0, 6, and 12 mo	77 hospitals in England, November 2011–June 2015	Adults hospitalized with MI	Terminal illness; “follow-up unsuitable”	Directly applicable Low RoB
Nam et al.^53^ (2015)	RCT	174	MI	Single	Six UK hospitals (not reported)	Recent non-ST elevation MI, risk of coronary artery disease	Past cardiac condition/CABG, treatment unsuitable, life expectancy <1 y	Partially applicable (A1) Low RoB
Phan et al.^54^ (2019)	Case series	1,914	Stroke, poststroke	1 and 5 y	4 incidence studies, Europe and Australasia, 1996–2013	All people experience a first stroke	Not adhering to reporting standards for stroke incidence studies	Partially applicable (A7) Low RoB
Pockett et al.^55^ (2018)	Case series	1,350	MI, UA, post-MI, post-UA	1, 6, and 12 mo	3 UK hospitals, January 2021–May 2021	Adults discharged within 1 mo following admission for MI/UA	Recent revascularization; type 1 diabetes	Directly applicable Low RoB
Roffe et al.^56^ (2018)	RCT	2,668	Stroke, poststroke	0, 3, 6, and 12 mo	136 UK hospitals with acute stroke wards (not reported)	Within 24 ho of admission and 48 h of stroke onset	Clear indications or contraindications, other life-threatening diseases	Directly applicable, potentially serious RoB (B6)
IST-3 Collaborative Group^57^ (2013)	RCT	1,179	Poststroke	18 mo from incidence	Multiple OECD countries including the United Kingdom, 2000–2011	Treatment promising but unproven, feasible to start <6 h	Previous imaging, structural brain lesions reminiscent of stroke	Partially applicable (A7) Low RoB
Shawo et al.^58^ (2020)	RCT	573	Stroke	Published area under the curve	Nineteen NHS study centers (not reported)	Adults receiving early supported discharge after stroke	Able to participate in rehabilitation focusing on activities of daily living	Partially applicable (A1,6) Potentially serious RoB (B5)
Squire et al.^59^ (2017)	Cross-sectional	191	Post–heart failure	Single	Seven centers in England, January 2015–May 2015	Adults diagnosed with chronic heart failure ≥12 mo previously	Unable to understand English, clinical trial participation or heart failure treatment	Partially applicable (A6) Low RoB
Walker et al.^60^ (2021)	RCT	1,202	Stable angina, post–stable angina	0, 6, 12, 24, 36 mo	6 UK hospitals November 2012–March 2015	Age ≥30 y, suspected stable angina suitable for revascularization	Clinically unstable, previous ACS or revascularization	Directly applicable Low RoB
Wallace et al.^61^ (2020)	RCT	28	Stroke	Single	Spasticity clinics, national neurologic center (not reported)	>1 mo since stroke, finger/wrist spasticity, potential benefit	Contraindications, upper-limb pain or disability, other neurologic impairment	Partially applicable (A1) Low RoB

ABPI, ankle brachial pressure index; ACS, acute coronary syndromes; CABG, coronary artery bypass graft; CVD, cardiovascular disease; GP, general practice; IC, intermittent claudication; MI, myocardial infarction; NHS, National Health Service; OECD, Organisation for Economic Cooperation and Development; PAD, peripheral arterial disease; PCI, percutaneous coronary intervention; RCT, randomized controlled trial; T2DM, type 2 diabetes mellitus; UA, unstable angina.

a Partial applicability criteria, see Appendix 7: A1, potentially unrepresentative; A6, EQ-5D-5L; A7, international health state descriptions.

b RoB criteria, see Appendix 7: B1, sample selection bias; B3, inappropriate handling of missing data; B5, mapping used; B6, partial proxy response.

Appendix 7 presents detailed study characteristics, including our assessments of applicability and risk of bias. We judged estimates from 14 studies to be directly applicable to a health state in the CG181 model and those from 15 to be partially applicable. The reasons for partial applicability were as follows. Eight had potentially unrepresentative populations (e.g., defined by eligibility for a treatment,^33,47,57,58^, by diagnosis of a condition subtype,^62^ or by symptoms rather than diagnoses^51^ such as spasticity after stroke^61^ or intermittent claudication for PAD^37,43^). Six used the EQ-5D-5L.^37,39,40,49,58,59^ Seven were international studies with some non-UK participants (although all used the UK tariff).^34,36,38,39,46,54,57^

We assessed risk of bias to be low for 20 studies and potentially serious in 9: 5 because of selection bias (e.g., due to low response rate,^37,39,43^ loss to follow-up,^46^ or recruiting volunteers only^45^), 3 because of partial proxy responses,^41,34,56^ and 1 because EQ-5D was mapped from another outcome measure.^58^

**Figure 2:**
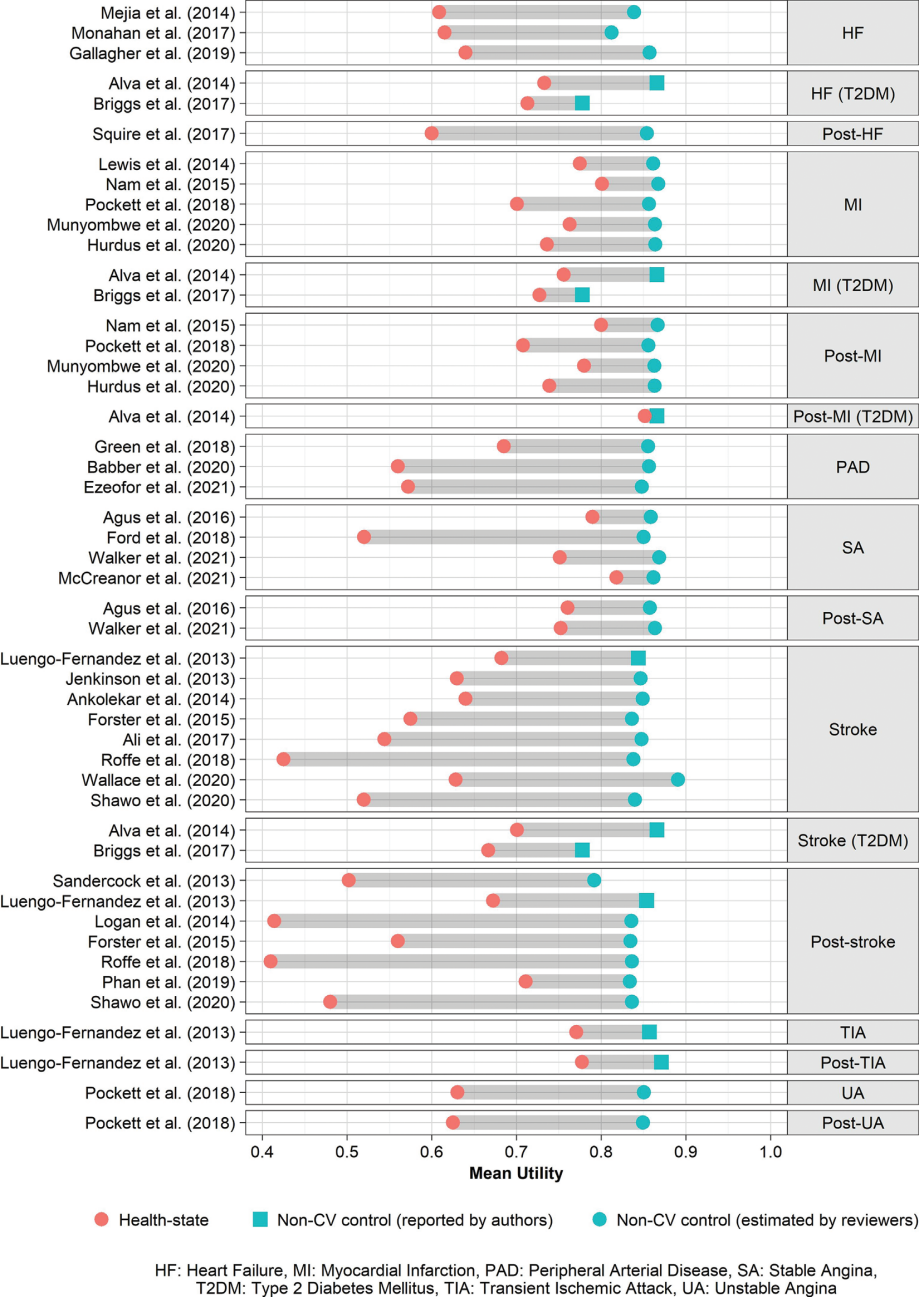
Mean utility for cardiovascular diseases and predicted baselines, UK EQ-5D estimates.

**Figure 3:**
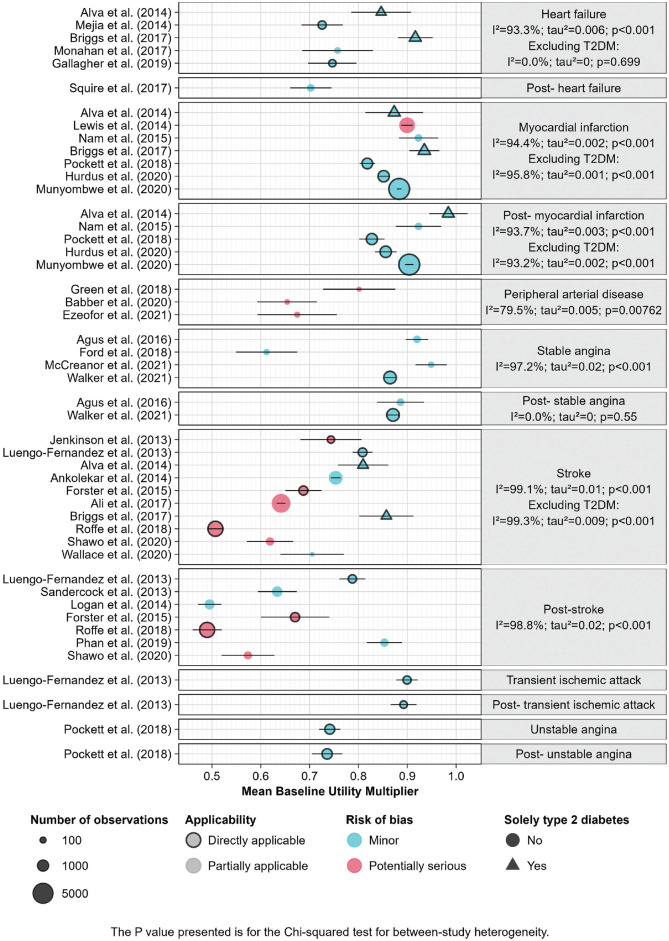
Mean baseline utility multipliers for cardiovascular diseases, UK EQ-5D estimates.

Figure 2 shows the HSUV for each study included in the analysis, compared with an age- and sex-matched baseline utility. Figure 3 shows the estimates of mean baseline utility multipliers for each health state, heterogeneity within health states, and relevant study characteristics. Except for the 2 estimates for post–stable angina (after the first year), health states that had multiple estimates showed substantial heterogeneity (*I* ^2^ ≥ 79.4%).

The baseline utility multiplier for the first year of stroke was smaller than that for later years in each study reporting both.^41,48,56,58^ First-year and later-year baseline utility multipliers were similar for MI, with the exception of 1 comprehensive study^52^ that estimated a smaller effect on utility for later years. There was no strong evidence for a difference between the baseline utility multipliers for the first and later years experiencing stable angina. We did not find any studies reporting estimates of utility after the first year experiencing heart failure or PAD and so assumed that the HSUV for these conditions was the same in later years.

The baseline utility multiplier estimates for heart failure and stroke among people with type 2 diabetes mellitus were smaller than those for people with no diabetes. It was not clear, however, whether comorbidities modified the effect of MI on utility, either in the first or later years.^35^

Studies that we classified as at higher risk of bias appeared to estimate larger baseline utility multipliers than those we classified as low risk. However, this satisfied only a conventional definition of between-stratum differences (*P* < 0.05 by partitioned heterogeneity test) within the stroke health state.

### Recommended Multipliers

Our preferred source for stroke baseline utility multipliers (first year 0.81 ± standard error 0.010, then 0.79 ± 0.014) and TIA (0.90 ± 0.011, then 0.89 ± 0.013) is a single case series recruiting participants and healthy population controls from primary care practices across one English county.^48^

We also prefer a single case series of people recently admitted to 3 British hospitals for acute coronary syndromes^55^ to calculate baseline utility multipliers for unstable angina (0.74 ± 0.011, then 0.74 ± 0.016) and MI (0.82 ± 0.008, then 0.83 ± 0.013). A case series ascertaining a high proportion of MIs in England over a 5-y period^52^ is also available (0.88 ± 0.002, then 0.90 ± 0.004). However, relying on the second study would compromise the face validity of the set of multipliers, as it implies that MI has less impact on utility than stable angina. Therefore, we prioritized the consistency between states provided by the first study^55^ and explored the impact of preferring the second^52^ in sensitivity analysis.

In the absence of case series, we recommend basing the effect of heart failure (both first year and after, 0.73 ± 0.021) on evidence from an RCT.^50^ This trial had broad eligibility criteria and recruited from various services across 2 sites in England. Similarly, we prefer evidence from an RCT conducted across several British hospitals^60^ to inform the baseline utility multiplier for stable angina (0.86 ± 0.006, then 0.87 ± 0.007). We did not identify any satisfactory UK estimates for PAD. Having reviewed available data from other countries, we selected an estimate (0.76 ± 0.018) derived from a Dutch population,^63^ as 1) we judged it to be relatively comparable to the UK setting and 2) the authors provide valuations according to the British tariff. We provide the same calculations and characteristics for this study as for UK studies in Appendix 4 (2) and Appendix 7 (2).

**Figure 4:**
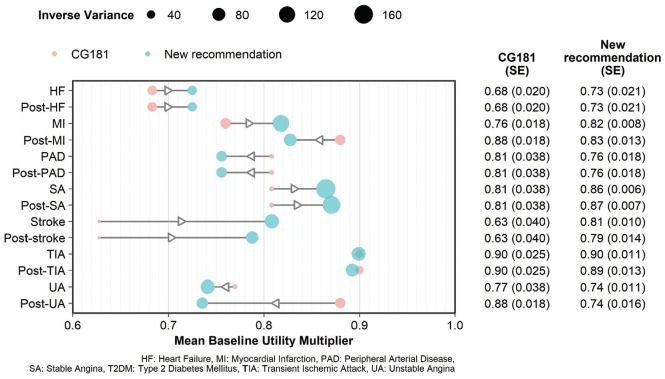
Recommended baseline utility multipliers compared with those NICE CG181 uses.

Figure 4 reports the baseline utility multipliers we recommend compared with those used in CG181. The largest differences are for stroke, which was the most severe state in CG181 but had a smaller effect and severity ranking in our recommendations. CG181 weighted utility estimates for mild, moderate, and severe strokes from a 2003 meta-analysis^64^ according to evidence from a UK RCT.^65^ However, that RCT excluded people with mild strokes, and severe cases predominated. In contrast, our preferred study of a comprehensively ascertained sample of people experiencing stroke in UK primary care reports that mild strokes are most common and severe ones rare.^48^ Weighting CG181’s HSUVs according to this case mix produces an HSUV estimate similar to our chosen source (0.77). For both MI and UA, the differences between first-year and later-year estimates are noticeably greater in the CG181 HSUVs than in our preferred values.

## Discussion

### Summary of Findings

We identified a great number and variety of utility estimates for CVD published since 2013. Although most studies used EQ-5D, 3 direct methods and indirect methods using other descriptive systems were also represented. Although most assessed UK or other OECD populations, many other countries were represented. We restricted our analysis to UK studies based on the EQ-5D and calculated baseline utility multipliers that were relative to age- and sex-matched controls. However, conspicuous heterogeneity in baseline utility multipliers remained the norm across types of CVD.

We can deduce that the observed heterogeneity most likely derives from differences in case mix, resulting from varying eligibility criteria. Case series emerged as the study type most likely to produce a representative sample of its intended population. However, we could not always determine the ways in which cohorts vary based on reported study characteristics. Most notably, baseline utility multipliers from Munyombwe et al.^52^ and Pockett et al.^55^ were considerably different, although both were case series enrolling participants hospitalized for MI.

Our recommendation of a study that produced a more severe baseline utility multiplier for unstable angina than for MI may benefit from further explanation.^55^ As well as wanting to preserve the relationship found within that study, we thought that there was a clinical rationale for the ordering. Although the initial effect of an MI may be substantial, rapid revascularization and other interventions may on average be more likely to limit ongoing angina symptoms than interventions for unstable angina.

### Comparison with Other Literature

Betts et al.^66^ updated Smith et al.’s^25^ systematic review of utility estimates for angina, MI, and stroke to 2018 and also included PAD. The authors summarized the distribution of estimates found, stratified by instrument, and analyzed trends over time. Another systematic review (2021) of HSUVs for heart failure^67^ also summarized the distribution of estimates and examined heterogeneity in population definitions, derivation methods, and statistics used for reporting. An international review^68^ (2021) of cost–utility analyses using published HSUVs for CVD between 1977 and 2016 stratified these by instrument and assessed whether models defined populations in a different way to the primary source. Lastly, a systematic review and meta-analysis (2022) of HSUVs for stroke critically appraised evidence, pooled estimates measured using each instrument, and explored effects of respondent characteristics on pooled estimates using stratification and meta-regression.^69^

Our quantitative analysis differed from those of previous reviews in 4 main ways. First, we focused on studies meeting a particular reference case. In particular, we excluded studies assessing subgroups that are not necessarily typical of a wider disease (e.g., heart failure with preserved ejection fraction). Second, we used AUC to combine utility estimates at different time points, whereas other reviews do not bring together longitudinal data. Third, we calculated baseline utility multipliers for use in economic models. Fourth, we did not meta-analyze estimates.

### Strengths and Limitations

We calculated baseline utility multipliers to suit a model with a 1-y cycle length and account for the effect of CVD on age- and sex-specific estimates of general population utility multiplicatively. This is a common way to apply HSUVs in decision models, and we hope that modelers will find the baseline utility multipliers provided useful. Presenting baseline utility multipliers will also discourage the use of absolute HSUVs as if they were multipliers (e.g., this is the case in the original CG181 model). Doing so will overstate the effects of health states on utility. The best way to represent the effect of chronic diseases such as heart failure, PAD, and stable angina on utility over time remains unclear, especially if (as we found) published evidence is cross-sectional or does not report time from diagnosis.

The model we updated contains health states with different preconceived levels of severity. For one health state (MI), we found that it was not possible to choose the study with the most comprehensive ascertainment (Munyombwe et al.^52^) without sacrificing face validity in the ordering of HSUVs between states. To ensure that we chose a consistent set of sources, we took a holistic view of evidence across states.

### Implications

The considerable unreported heterogeneity we found emphasizes the importance of 1) using systematic reviews to identify utility evidence for cost–utility models and 2) converting estimates identified to baseline utility multipliers to provide a range of comparable values. Modelers who choose HSUVs using rapid searches for plausible values are very likely to rely on values that differ from others they might have found for reasons over and above sampling error. This is illustrated by the comparison between our recommended baseline utility multipliers and those used in the original CG181 model. Future research could investigate the range of potential baseline estimates that could be used for cost-utility models and explore the impact of using updated baselines, reflecting trends and changes in the health status of the general population.

One question arising after reviewing the evidence is whether to synthesize available estimates or choose individual sources. Meta-analysis offers a way of synthesizing multiple credible estimates of utility for a population, given these have been derived using the same instrument. The prevailing advice is that analysts should consider quantitative synthesis of HSUVs only if they constrain their evidence base to homogeneous settings.^70^ Our review met these criteria, including only studies from UK populations measured using EQ-5D-3L valued using the same UK tariff. Nevertheless, we observed obvious heterogeneity in the values that we found, which we suspect to be due to differences in participant selection. We therefore decided not to meta-analyze the results. Unreported heterogeneity also meant that meta-regression to explore the effect of reported characteristics on baseline utility multipliers would be inappropriate.^70^

The factors that modelers consider in choosing individual utility sources should be informed by their particular decision context. Our critical appraisal tool assesses studies based on not only their risk of bias but also their applicability to the NICE reference case. We urge reviewers working in other jurisdictions to adapt the applicability criteria to suit local guidelines. Future research is also needed to refine the tool, for example, to explore whether specific study types such as case series should be preferred. Even so, such a tool should be used only to provide a list of candidate sources from which to choose utility evidence. Modelers should still judge candidates based on factors such as population ascertainment and, in multistate models, face validity of HSUV severity orderings. It is also important to explore the effect of prioritizing each of these factors using sensitivity analyses.

Lastly, modelers must decide whether to model changes in utility over time since a cardiovascular event or diagnosis. Markov models often simulate immediate utility associated with the incidence of a disease in one cycle and a separate utility in later cycles. This is a natural approach for acute diseases such as acute coronary events and stroke but may not be appropriate for chronic diseases.

## Conclusions

A previous cost-utility model of preventative treatment for CVD^22^ identified HSUV evidence using a systematic review. We updated this review, cataloged international CVD utility estimates, and calculated UK-relevant baseline utility multipliers for the 7 health states modeled. We identified many studies assessing utility for CVD in a variety of countries (mostly OECD) and using a variety of methods (mostly EQ-5D). For each condition, there was considerable heterogeneity in the baseline utility multipliers that we derived from UK studies using the EQ-5D. Formal assessment of applicability or risk of bias only partially explained this heterogeneity.

Primary studies do not always report sources of heterogeneity, such as recruitment factors leading to case-mix differences. Therefore, a systematic review and critical appraisal of utility values may not be enough to ensure that modelers choose the most appropriate set of estimates. To select a set of baseline utility multipliers, we also needed to consider the face validity of suitable estimates relative to other available estimates for the same disease as well as those for related diseases. We advise that future modelers generating economic evidence relating to CVD use our recommended baseline utility multipliers or follow similar principles to estimate evidence fitting their requirements.

## Supplemental Material

sj-docx-1-mdm-10.1177_0272989X231214782 – Supplemental material for International Systematic Review of Utility Values Associated with Cardiovascular Disease and Reflections on Selecting Evidence for a UK Decision-Analytic Model Click here for additional data file. Supplemental material, sj-docx-1-mdm-10.1177_0272989X231214782 for International Systematic Review of Utility Values Associated with Cardiovascular Disease and Reflections on Selecting Evidence for a UK Decision-Analytic Model by Rob Hainsworth, Alexander J. Thompson, Bruce Guthrie, Katherine Payne and Gabriel Rogers in Medical Decision Making
