# Supplementary material for: International Systematic Review of Utility Values Associated with Cardiovascular Disease and Reflections on Selecting Evidence for a UK Decision-Analytic Model
Source: Med Decis Making. 2024 Jan 4;44(2):217–34. doi: 10.1177/0272989X231214782 (PMC10865747; doi:10.1177/0272989X231214782)
Supplement: sj-docx-1-mdm-10.1177_0272989X231214782 – Supplemental material for International Systematic Review of Utility Values Associated with Cardiovascular Disease and Reflections on Selecting Evidence for a UK Decision-Analytic Model [file sj-docx-1-mdm-10.1177_0272989X231214782.docx]

Appendix 1- Search strategy

We searched Medline and Embase between 01/01/2013 and the date of the search (22/04/2021).

**Medline search filter to identify studies reporting health state utility values**

1. Quality-Adjusted Life Years/

2. (quality adjusted or adjusted life year$).ti,ab,kf.

3. (qaly$ or qald$ or qale$ or qtime$).ti,ab,kf.

4. (illness state$1 or health state$1).ti,ab,kf.

5. (hui or hui1 or hui2 or hui3).ti,ab,kf.

6. (multiattribute$ or multi attribute$).ti,ab,kf.

7. (utility adj3 (score$1 or valu$ or health$ or cost$ or measur$ or disease$ or mean or gain or gains or index$)).ti,ab,kf.

8. utilities.ti,ab,kf.

9. (eq5d or eq5d or eq-5 or eq5 or euro qual or euroqual or euro qual5d or euroqual5d or euro qol or euroqol or euro qol5d or euroqol5d or euro quol or euroquol or euro quol5d or euroquol5d or eur qol or eurqol or eur qol5d or eur qol5d or eur?qul or eur?qul5d or euro$ quality of life or European qol).ti,ab,kf.

10. (euro$ adj3 (5 d or 5d or 5 dimension$ or 5dimension$ or 5 domain$ or 5domain$)).ti,ab,kf.

11. (sf36$ or sf 36$ or sf thirtysix or sf thirty six).ti,ab,kf.

12. (time trade off$1 or time tradeoff$1 or tto or timetradeoff$1).ti,ab,kf.

13. or/1-12

**Source:** Arber M, Garcia S, Veale T, Edwards M, Shawo A, Glanville JM. Performance of Ovid Medline search filters to identify health state utility studies. Int J Technol Assess Health Care 2017;33(4):472-480

**Embase search filter to identify studies reporting utility values**

1. Quality-Adjusted Life Year/

2. (quality adjusted or adjusted life year$).ti,ab,kw.

3. (qaly$ or qald$ or qale$ or qtime$).ti,ab,kw.

4. (illness state$1 or health state$1).ti,ab,kw.

5. (hui or hui1 or hui2 or hui3).ti,ab,kw.

6. (multiattribute$ or multi attribute$).ti,ab,kw.

7. (utility adj3 (score$1 or valu$ or health$ or cost$ or measur$ or disease$ or mean or gain or gains or index$)).ti,ab,kw.

8. utilities.ti,ab,kw.

9. (eq5d or eq5d or eq-5 or eq5 or euro qual or euroqual or euro qual5d or euroqual5d or euro qol or euroqol or euro qol5d or euroqol5d or euro quol or euroquol or euro quol5d or euroquol5d or eur qol or eurqol or eur qol5d or eur qol5d or eur?qul or eur?qul5d or euro$ quality of life or european qol).ti,ab,kw.

10. (euro$ adj3 (5 d or 5d or 5 dimension$ or 5dimension$ or 5 domain$ or 5domain$)).ti,ab,kw.

11. (sf36$ or sf 36$ or sf thirtysix or sf thirty six).ti,ab,kw.

12. (time trade off$1 or time tradeoff$1 or tto or timetradeoff$1).ti,ab,kw.

13. quality of life/ and ((quality of life or qol) adj (score$1 or measure$1)).ti,ab,kw.

14. quality of life/ and ec.fs.

15. quality of life/ and (health adj3 status).ti,ab,kw.

16. quality of life or qol).ti,ab,kw. and Cost-Benefit Analysis/

17. ((qol or utility or quality of life).ti,kw. or *quality of life/) and ((qol or utility$ or quality of life) adj2 (increas$ or decrease$ or improv$ or declin$ or reduc$ or high$ or low$ or effect or effects or worse or score or scores or change$1 or impact$1 or impacted or deteriorat$)).ab.

18. Cost-Benefit Analysis/ and (cost-effectiveness ratio$ and (perspective$ or life expectanc$)).ti,ab,kw.

19. *quality of life/ and (quality of life or qol).ti.

20. quality of life/ and ((quality of life or qol) adj3 (improv$ or chang$)).ti,ab,kw.

21. quality of life/ and health-related quality of life.ti,ab,kw.

22. models,economic/

23. (utility loss$ or disutilit$ or short form$ or shortform$ or SF-12 or SF12).ti,ab,kw.

24. or/1-23

|  |
| --- |

**Source:** Personal communication Julie M Glanville, 11 March 2021

**Medline search filter for relevant health states and diseases.** Source**: NICE CG181** Lipid modification: Cardiovascular risk assessment and the modification of blood lipids for the primary and secondary prevention of cardiovascular disease **(2014)**, **Appendix C**

1. cardiovascular diseases/

2. heart diseases/

3. myocardial ischemia/

4. exp angina pectoris/

5. coronary disease/

6. coronary artery disease/

7. exp coronary stenosis/

8. myocardial infarction/

9. exp heart failure/

10. arrhythmias, cardiac/ or atrial fibrillation/

11. vascular diseases/

12. hypertension/

13. atherosclerosis/

14. peripheral arterial disease/

15. peripheral vascular diseases/

16. cerebrovascular disorders/

17. exp stroke/

18. exp brain ischemia/

19. ((cardiovascular or cardio-vascular or cardio vascular) adj3 (event$ or disease$)).ti,ab.

20. ((coronary or peripheral vascular or heart or peripheral arter$) adj3 (disease$ or event$)).ti,ab.

21. (mi or myocardial infarct$).ti,ab.

22. (cvd or chd or cad or pad or cva or hypertension).ti.

23. (atheroscleros$ or arterioscleros$).ti,ab.

24. (cerebrovascular accident$ or stroke$).ti,ab.

25. (acs or angina or acute coronary syndrome$).ti,ab.

26. (af or atrial fibrillation).ti,ab.

27. ((chronic or congestive) adj2 heart failure).ti,ab.

28. or/1-27

**Embase search filter for relevant health states and diseases.** Source**: NICE CG181** Lipid modification: Cardiovascular risk assessment and the modification of blood lipids for the primary and secondary prevention of cardiovascular disease **(2014)**, **Appendix C**

1. *cardiovascular disease/

2. *coronary artery disease/

3. *vascular disease/

4. *coronary artery atherosclerosis/

5. *peripheral vascular disease/

6. *peripheral occlusive artery disease/

7. *arteriosclerosis/

8. *ischemic heart disease/

9. exp *stroke/ or *stroke patient/

10. *coronary artery obstruction/

11. *hypertension/

12. *heart disease/

13. *heart arrhythmia/

14. *heart fibrillation/ or *heart atrium fibrillation/

15. *heart failure/ or exp *congestive heart failure/

16. *acute coronary syndrome/ or exp *angina pectoris/ or *heart infarction/

17. *cerebrovascular disease/

18. *cerebrovascular accident/

19. exp *brain ischemia/

20. *brain infarction/

21. *atherosclerosis/

22. exp *cardiovascular risk/

23. ((cardiovascular or cardio-vascular or cardio vascular) adj3 (event$ or disease$)).ti,ab.

24. ((coronary or peripheral vascular or heart or peripheral arter$) adj3 (disease$ or event$)).ti,ab.

25. (MI or myocardial infarct$).ti,ab.

26. (CVD or CHD or CAD or PAD or CVA or hypertension).ti.

27. (atheroscleros$ or arterioscleros$).ti,ab.

28. (cerebrovascular accident$ or stroke$).ti,ab.

29. (ACS or angina or acute coronary syndrome$).ti,ab.

30. (AF or atrial fibrillation).ti,ab.

31. ((chronic or congestive) adj2 heart failure).ti,ab.

32. or/1-32

Appendix 2- Critical appraisal instrument

| **Criterion** | **Score** | **Notes on interpretation** |
| --- | --- | --- |
| [SECTION A: APPLICABILITY[1]](file:///C:\\Users\\mbmhtrh2\\Dropbox\\primary%20prevention\\cvd_util_review_data_extraction_22sep.xlsm#RANGE!A61) | | |
| A1.   Are health-states for which HSUV(s) are presented representative of modelled states? | Yes |  |
|  | Partially | All participants are within the defined health-state(s), but may not represent the full spectrum of relevant event(s) |
|  | No | This is grounds for exclusion |
| A2.   Are timepoints at which HSUV(s) are measured representative of modelled states? | Yes | Over 12mo following event (for event state) and/or >12mo (for post-event state) |
|  | Partially | Covers some of first 12mo only |
|  | No | This is grounds for exclusion |
| A3.   Are HSUVs presented for more than 1 state of interest? | Yes |  |
|  | No |  |
| A4.   Are HSUVs presented for controls without the event(s) of interest? | Yes |  |
|  | No |  |
| A5.   Are data presented with mean and an appropriate measure of dispersion? | Yes | Mean + SE/SD/CI |
|  | No | Medians, ranges, no measure of dispersion |
| INDIRECT VALUATION | | |
| A6.   Is EQ5D-3L used? | Yes |  |
|  | Via mapping |  |
|  | No |  |
| A7. Are health-state descriptions provided by a UK population? | Yes |  |
|  | Partially | International population including UK |
|  | No |  |
| A8.   Does valuation reflect UK societal preferences? | Yes | Use of appropriate UK tariff |
|  | No |  |
| DIRECT VALUATION | | |
| A9.   Does valuation use a choice-based method? | Yes | Ideally (per old NICE methods), TTO with 'full health' as the upper anchor ('to retain methodological consistency with the methods used to value the EQ5D') |
|  | No |  |
| A10. Does valuation reflect UK societal preferences? | Yes | Participants representative of UK society |
|  | No |  |
| **Overall judgement on applicability** | Directly applicable | No major concerns |
|  | Partially applicable | Fails to meet 1 or more of the applicability criteria, and this could change the conclusions |
|  | Not applicable | This is grounds for exclusion |
| **SECTION B: RISK OF BIAS (RoB** | | |
| B1.   Could the selection of patients have introduced bias? | Low RoB | A study should ideally enrol all consecutive, or a random sample of, eligible participants. Eligibility criteria should not exclude subgroups of relevant people (e.g. those with milder or more severe events). |
|  | High RoB |  |
| B2.   Were all enrolled participants included in the analysis? | Low RoB | All enrolled participants included in the analysis |
|  | High RoB | Some or a subgroup of participants are inappropriately excluded from the analysis |
| B3.   Were participants with missing data handled appropriately? | Low RoB | [No missing data (most likely in cross-sectional analyses) or appropriate handling of missing data (e.g. multiple imputation for longitudinal studies)[2]](file:///C:\\Users\\mbmhtrh2\\Dropbox\\primary%20prevention\\cvd_util_review_data_extraction_22sep.xlsm#RANGE!A62) |
|  | Moderate RoB | Imputation undertaken, but subject to limitations |
|  | High RoB | Complete-case analysis only |
| INDIRECT VALUATION | | |
| B4.  Can HSUVs be extracted independently of investigators' focus, where necessary? | Low RoB | E.g. in intervention studies, can we obtain HSUVs that do not reflect effects of experimental treatment(s)? |
|  | High RoB | If data cannot be disentangled, this may be grounds for exclusion |
| B5.  Was mapping between instruments undertaken appropriately? | Low RoB | [No mapping or mapping appropriately undertaken[3]](file:///C:\\Users\\mbmhtrh2\\Dropbox\\primary%20prevention\\cvd_util_review_data_extraction_22sep.xlsm#RANGE!A63) |
|  | Moderate RoB | Mapping subject to limitations (e.g. with summary scores rather than IPD) |
|  | High RoB | Unvalidated or inappropriate mapping approach |
| B6.   Are HSUVs measured directly by patients experiencing the event(s) of interest? | Yes |  |
|  | No – carers |  |
|  | No – HCPs |  |
| DIRECT VALUATION | | |
| B7.  Were there clearly described, appropriate methods for generating the health-state descriptions that participants valued? | Low RoB | [Descriptions based on literature and/or qualitative interviews with people with experiential insight (including patients)[4]](file:///C:\\Users\\mbmhtrh2\\Dropbox\\primary%20prevention\\cvd_util_review_data_extraction_22sep.xlsm#RANGE!A64) |
|  | High RoB | Not described or predominantly based on authors’ own views |
| B8.  Did preference elicitation use an established protocol? | Low RoB | [E.g. Measurement and Valuation of Health (MVH), the Paris protocol and the EuroQol Valuation Technology (EQ-VT) protocols for TTO[5]](file:///C:\\Users\\mbmhtrh2\\Dropbox\\primary%20prevention\\cvd_util_review_data_extraction_22sep.xlsm#RANGE!A65) |
|  | High RoB | Unreported or ad hoc methods |
| B9.  Was the elicitation exercise piloted? | Low RoB | Yes |
|  | High RoB | No |
| **Overall judgement** | Low RoB | The study is at low RoB against all criteria, or fails to meet 1 or more criteria but this is unlikely to change conclusions. |
| **on risk of bias** | Potentially  serious RoB | The study fails to meet 1 or more criteria and this could change conclusions. |
|  |  |  |
|  | Serious RoB | The study fails to meet 1 or more criteria and this is likely to change conclusions. |
|  | | |
|  |  |  |

[[1]](file:///C:\\Users\\mbmhtrh2\\Dropbox\\primary%20prevention\\cvd_util_review_data_extraction_22sep.xlsm#RANGE!A2)  Judged against (a) the decision-problem addressed by our model (primary prevention of cardiovascular disease, including angina, MI, stroke, TIA, heart failure and PAD), and (b) the NICE reference case for estimating cost effectiveness <https://www.nice.org.uk/process/pmg9/chapter/the-reference-case#measuring-and-valuing-health-effects>

[[2]](file:///C:\Users\mbmhtrh2\Dropbox\primary%20prevention\cvd_util_review_data_extraction_22sep.xlsm#RANGE!C39) See <https://doi.org/10.1007/s11136-014-0837-y>

[[3]](file:///C:\Users\mbmhtrh2\Dropbox\primary%20prevention\cvd_util_review_data_extraction_22sep.xlsm#RANGE!C45) See <https://doi.org/10.1016/j.jval.2016.11.006>

[[4]](file:///C:\Users\mbmhtrh2\Dropbox\primary%20prevention\cvd_util_review_data_extraction_22sep.xlsm#RANGE!C49)  See <https://doi.org/10.1016/j.jval.2020.12.017>

[[5]](file:///C:\Users\mbmhtrh2\Dropbox\primary%20prevention\cvd_util_review_data_extraction_22sep.xlsm#RANGE!C51) See https://doi.org/10.1007/s40273-016-0404-1

Appendix 3- Model to predict utility in people aged 16–89 with no history of cardiovascular disease from Health Survey for England data

As an example, I will show how to calculate the utility of population with no previous cardiovascular disease, a mean age of 70 and 45% men. The value for the Male parameter will be 0.45, and the value for Age will be 70. You can enter these into the following formula to calculate their mean utility as 0.8404.

$1.0800+\left( Male\times0.2198 \right)+\left( Age\times-0.01805 \right)+ {(Age}^{2}\times9.591 \times{10}^{-4})+ {(Age}^{3}\times-2.566 \times{10}^{-5})+ {(Age}^{4}\times3.112\times{10}^{-7}) + {(Age}^{5}\times-1.409\times{10}^{-9}) +(Male\times Age\times-0.02696)+ {(Male\times Age}^{2}\times1.278\times{10}^{-3}) + {(Male\times Age}^{3}\times-2.851\times{10}^{-5}) + {(Male\times Age}^{4}\times3.067\times{10}^{-7})+ {(Male\times Age}^{5}\times-1.268\times{10}^{-9})$

$$1.0800+\left( 0.45\times0.2198 \right)+\left( 70\times-0.01805 \right)+ {(70}^{2}\times9.591 \times{10}^{-4})+ {(70}^{3}\times-2.566 \times{10}^{-5})+ {(70}^{4}\times3.112 \times{10}^{-7}) + {(70}^{5}\times-1.409\times{10}^{-9}) +(0.45\times70\times-0.02696)+ {(0.45\times70}^{2}\times1.278\times{10}^{-3}) + {(0.45\times70}^{3}\times-2.851\times{10}^{-5}) + {(0.45\times70}^{4}\times3.067\times{10}^{-7})+ {(0.45\times70}^{5}\times-1.268\times{10}^{-9})=0.8404$$

| Parameter | EQ5D-3L (95% confidence interval) |
| --- | --- |
| Constant | 1.0800 (0.8332, 1.3268) |
| Male | 0.2198 (−0.1610, 0.6006) |
| Age | −0.01805 (−0.0496, 0.0135) |
| Age^2^ | 9.591×10^−4^ (−5.37×10^−4^, 2.46×10^−3^) |
| Age^3^ | −2.566×10^−5^ (−5.89×10^−5^, 7.60×10^−6^) |
| Age^4^ | 3.112×10^−7^ (−3.81×10^−8^, 6.60×10^−7^) |
| Age^5^ | −1.409×10^−9^ (−2.81×10^−9^, −1.29×10^−11^) |
| Male × Age | −0.02696 (−0.0759, 0.0220) |
| Male × Age^2^ | 1.278×10^−3^ (−1.06×10^−3^, 3.61×10^−3^) |
| Male × Age^3^ | −2.851×10^−5^ (−8.07×10^−5^, 2.37×10^−5^) |
| Male × Age^4^ | 3.067×10^−7^ (−2.45×10^−7^, 8.59×10^−7^) |
| Male × Age^5^ | −1.268×10^−9^ (−3.49×10^−9^, 9.55×10^−10^) |
| Superscript numbers denote power terms e.g. Age^2^ means age squared. | |

Appendix 4 - Calculation tables of annual baseline utility adjustments for EQ5D-based (1) UK studies and (2) studies in similar populations

| **UK studies** | | | | | | | | | |
| --- | --- | --- | --- | --- | --- | --- | --- | --- | --- |
| **Study, disease \| Arm (mean age, % men)** | | **Month** | **Raw utility** | **Number of participants** | **Modelled distribution**** | **Transformed utility (standard error)** | | **Predicted baseline utility** | **Baseline utility adjustment (standard error)** |
| Agus et al. (2016), stable angina (59.4, 38%) | | 0 | 0.79 (s.e.= 0.010) | 188 | x~Beta(2522.63, 382.77) | Direct= 0.79 (0.010) | | 0.86 | 0.92 (0.012) |
| Agus et al. (2016), post-stable angina (59.9, 38%) | | 12 | 0.76 (s.d.= 0.290) | 188* | x~Beta(616.20, 109.22) | Direct= 0.76 (0.021) | | 0.86 | 0.89 (0.025) |
| Ali et al. (2017), stroke (72.5, 55%) | Modified Rankin Scale=0 | 0 | 0.90 (s.d.= 0.170) | 529 | x~Beta(2562.19, 171.50) | Pooled= 0.54 (0.004) | | 0.85 | 0.64 (0.004) |
|  | Modified Rankin Scale =1 | 0 | 0.82 (s.d.= 0.190) | 866 | x~Beta(5415.30, 689.36) |  |  |  |  |
|  | Modified Rankin Scale =2 | 0 | 0.70 (s.d.= 0.210) | 633 | x~Beta(4522.61, 1048.52) |  |  |  |  |
|  | Modified Rankin Scale =3 | 0 | 0.53 (s.d.= 0.260) | 669 | x~Beta(3685.86, 1541.24) |  |  |  |  |
|  | Modified Rankin Scale =4 | 0 | 0.20 (s.d.= 0.310) | 825 | x~Beta(2715.77, 2736.30) |  |  |  |  |
|  | Modified Rankin Scale =5 | 0 | -0.15 (s.d.= 0.230) | 336 | x~Beta(903.08, 2339.05) |  |  |  |  |
| Alva et al. (2014), MI | Wooldridge fixed effects | 0 | Additive decrement= 0.11 (s.e. = 0.026) | 364 | Sample directly from Normal(0.11, 0.026) | No transformation | | 0.87 | 0.87 (0.030) |
| Alva et al. (2014), post-MI | Wooldridge fixed effects | 0 | Additive decrement= 0.01 (s.e. = 0.017) | 364* | Sample directly from Normal(0.01, 0.017) | No transformation | | 0.87 | 0.98 (0.020) |
| Alva et al. (2014), stroke | Wooldridge fixed effects | 0 | Additive decrement= 0.17 (s.e. = 0.023) | 226 | Sample directly from Normal(0.17, 0.023) | No transformation | | 0.87 | 0.81 (0.027) |
| Alva et al. (2014), heart failure | Wooldridge fixed effects | 0 | Additive decrement= 0.13 (s.e. = 0.027) | 181 | Sample directly from Normal(0.13, 0.027) | No transformation | | 0.87 | 0.85 (0.031) |
| Ankolekar et al. (2014), stroke (69.2, 60%) | | 0 | 0.64 (s.d.= 0.171) | 1571 | x~Beta(18493.75, 5395.26) | Direct= 0.64 (0.004) | | 0.85 | 0.75 (0.005) |
| Babber et al. (2020), PAD | Active (66.5, 70%) | 0 | 0.56 (s.d.= 0.160) | 21 | x~Beta(300.82, 114.70) | Pooled= 0.56 (0.027) | | 0.86 | 0.65 (0.032) |
|  | Placebo (68.5, 68%) | 0 | 0.56 (s.d.= 0.190) | 22 | x~Beta(223.30, 85.14) |  |  |  |  |
| Briggs et al. (2017), MI | | 0 | Additive decrement= 0.05 (s.e. = 0.012) | 415 | Sample directly from Normal(0.05, 0.012) | No transformation | | 0.78 | 0.93 (0.015) |
| Briggs et al. (2017), stroke | | 0 | Additive decrement= 0.11 (s.e. = 0.022) | 208 | Sample directly from Normal(0.11, 0.022) | No transformation | | 0.78 | 0.86 (0.029) |
| Briggs et al. (2017), heart failure | | 0 | Additive decrement= 0.07 (s.e. = 0.014) | 373 | Sample directly from Normal(0.07, 0.014) | No transformation | | 0.78 | 0.92 (0.018) |
| Ezeofor et al. (2021), PAD | Men int. (71.2, 100%) | 0 | 0.53 (s.d.= 0.315) | 22 | x~Beta(81.83, 34.20) | Pooled= 0.57 (0.036) | | 0.85 | 0.67 (0.042) |
|  | Women int. (71.2, 0%) | 0 | 0.40 (s.d.= 0.321) | 12 | x~Beta(42.72, 26.01) |  |  |  |  |
|  | Men cntrl. (73.0, 100%) | 0 | 0.70 (s.d.= 0.228) | 22 | x~Beta(133.15, 31.12) |  |  |  |  |
|  | Women cntrl. (73.0, 0%) | 0 | 0.62 (s.d.= 0.182) | 5 | x~Beta(52.08, 16.15) |  |  |  |  |
| Ford et al. (2018), stable angina (61.0, 26%) | | 0 | 0.58 (s.d.= 0.300) | 75 | x~Beta(301.90, 108.00) | AUC= 0.52 (0.027) | | 0.85 | 0.61 (0.032) |
|  |  | 6 | 0.50 (s.d.= 0.400) | 75 | x~Beta(175.29, 80.11) |  |  |  |  |
|  |  | 12 | 0.50 (s.d.= 0.400)* | 75* | x~Beta(177.64, 81.19) |  |  |  |  |
| Forster et al. (2015), stroke (72.5, 55%) | | 0 | 0.58 (s.e.= 0.025)* | 399* | x~Beta(580.32, 207.61) | AUC= 0.58 (0.016) | | 0.84 | 0.69 (0.019) |
|  |  | 6 | 0.58 (s.e.= 0.025) | 399* | x~Beta(580.32, 207.61) |  |  |  |  |
|  |  | 12 | 0.56 (s.e.= 0.030) | 399* | x~Beta(407.72, 155.46) |  |  |  |  |
| Forster et al. (2015), post-stroke (73.5, 55%) | | 12 | 0.56 (s.e.= 0.030) | 399* | x~Beta(407.72, 155.46) | Direct= 0.56 (0.030) | | 0.86 | 0.67 (0.036) |
| Gallagher et al. (2019), heart failure (68.3, 73%) | | 0 | 0.64 (s.d.= 0.270) | 152 | x~Beta(716.29, 208.97) | Direct= 0.64 (0.022) | | 0.86 | 0.75 (0.025) |
| Green et al. (2018), PAD | Active (67.5, 60%) | 0 | 0.72 (s.d.= 0.150) | 15 | x~Beta(201.37, 42.91) | Pooled= 0.69 (0.032) | | 0.85 | 0.80 (0.037) |
|  | Placebo (64.3, 60%) | 0 | 0.65 (s.d.= 0.200) | 15 | x~Beta(126.48, 35.46) |  |  |  |  |
| Green et al. (2018), post-PAD | Placebo (68.5, 60%) | 12 | 0.64 (s.d.= 0.170) | 13 | x~Beta(153.93, 44.91) | Direct= 0.64 (0.048) | | 0.85 | 0.75 (0.056) |
| Hurdus et al. (2020), MI (63.6, 75%) | Cardio. rehab | 0 | 0.77 (s.d.= 0.264) | 1681 | x~Beta(6548.00, 1126.64) | Unused | | N/a | N/a |
|  |  | 1 | 0.77 (s.d.= 0.232) | 2259 | x~Beta(11168.16, 1854.55) |  |  |  |  |
|  |  | 6 | 0.82 (s.d.= 0.236) | 1862 | x~Beta(7515.92, 950.78) |  |  |  |  |
|  |  | 12 | 0.83 (s.d.= 0.225) | 1725 | x~Beta(7301.82, 860.24) |  |  |  |  |
|  | No cardio. rehab | 0 | 0.75 (s.d.= 0.277) | 874 | x~Beta(3193.47, 582.79) | AUC= 0.74 (0.006) | | 0.86 | 0.85 (0.007) |
|  |  | 1 | 0.73 (s.d.= 0.278) | 874 | x~Beta(3371.77, 693.74) |  |  |  |  |
|  |  | 6 | 0.74 (s.d.= 0.290) | 885 | x~Beta(3075.06, 607.62) |  |  |  |  |
|  |  | 12 | 0.74 (s.d.= 0.294) | 887 | x~Beta(2984.83, 584.43) |  |  |  |  |
| Hurdus et al. (2020), post-MI (64.6, 75%) | No cardio. rehab | 12 | 0.74 (s.d.= 0.294) | 887 | x~Beta(2984.83, 584.43) | Direct= 0.74 (0.010) | | 0.86 | 0.86 (0.011) |
| Jenkinson et al. (2013), stroke (70.0, 58%) | | 0 | 0.63 (s.d.= 0.330) | 145 | x~Beta(462.27, 139.74) | Direct= 0.63 (0.027) | | 0.85 | 0.74 (0.032) |
| Lewis et al. (2014), MI | Subsequent event (68.6, 69%) | 0 | 0.70 (s.d.= 0.290) | 597 | x~Beta(2236.26, 518.45) | Pooled= 0.77 (0.005) | | 0.86 | 0.90 (0.006) |
|  | No subsequent event (63.1, 76%) | 0 | 0.80 (s.d.= 0.230) | 1785 | x~Beta(8226.28, 1180.24) |  |  |  |  |
| Logan et al. (2014), Post-stroke (71.7, 42%) | Int (71.7, 42%) | 24.5 (median) | 0.41 (s.d.= 0.240) | 281 | x~Beta(1821.49, 1076.17) | AUC= 0.41 (0.010) | | 0.84 | 0.50 (0.012) |
|  | Cntrl (71.5, 47%) | 21.3 (median) | 0.42 (s.d.= 0.242) | 280 | x~Beta(1782.90, 1019.81) |  |  |  |  |
| Luengo-Fernandez et al. (2013), stroke (75.0, 49%) | | 0 | 0.64 (s.d.= 0.330) | 444 | x~Beta(1401.39, 408.83) | AUC= 0.68 (0.009) | | N/a, see stroke control row | 0.81 (0.010) |
|  |  | 1 | 0.64 (s.d.= 0.330) | 445 | x~Beta(1404.55, 409.76) |  |  |  |  |
|  |  | 6 | 0.70 (s.d.= 0.290) | 339 | x~Beta(1269.48, 294.32) |  |  |  |  |
|  |  | 12 | 0.70 (s.d.= 0.270) | 418 | x~Beta(1806.16, 418.74) |  |  |  |  |
| Luengo-Fernandez et al. (2013), post-stroke | | 12 | 0.70 (s.d.= 0.270) | 418 | x~Beta(1806.16, 418.74) | AUC= 0.67 (0.012) | | N/a, see post-stroke control row | 0.79 (0.014) |
|  |  | 24 | 0.66 (s.d.= 0.290) | 263 | x~Beta(1048.14, 284.18) |  |  |  |  |
|  |  | 60 | 0.68 (s.d.= 0.310) | 269 | x~Beta(911.27, 228.89) |  |  |  |  |
| Luengo-Fernandez et al. (2013) ), non-CVD control for stroke | | 0 | 0.83 (s.d.= 0.230) | 380 | x~Beta(1552.60, 185.35) | AUC= 0.84 | | N/a | N/a |
|  |  | 1 | 0.83 (s.d.= 0.230) | 381 | x~Beta(1556.68, 185.84) |  |  |  |  |
|  |  | 6 | 0.85 (s.d.= 0.230) | 306 | x~Beta(1134.11, 117.81) |  |  |  |  |
|  |  | 12 | 0.85 (s.d.= 0.230) | 368 | x~Beta(1364.08, 141.70) |  |  |  |  |
| Luengo-Fernandez et al. (2013), non-CVD control for post-stroke | | 12 | 0.85 (s.d.= 0.230) | 368 | x~Beta(1364.08, 141.70) | AUC= 0.85 | | N/a | N/a |
|  |  | 24 | 0.85 (s.d.= 0.220) | 235 | x~Beta(951.80, 98.87) |  |  |  |  |
|  |  | 60 | 0.86 (s.d.= 0.220) | 241 | x~Beta(923.66, 88.94) |  |  |  |  |
| Luengo-Fernandez et al. (2013), TIA (75.0, 49%) | | 0 | 0.78 (s.d.= 0.250) | 313 | x~Beta(1304.02, 208.80) | AUC= 0.77 (0.009) | | N/a, see TIA control row | 0.90 (0.011) |
|  |  | 1 | 0.78 (s.d.= 0.250) | 314 | x~Beta(1308.19, 209.46) |  |  |  |  |
|  |  | 6 | 0.76 (s.d.= 0.270) | 244 | x~Beta(923.05, 163.61) |  |  |  |  |
|  |  | 12 | 0.78 (s.d.= 0.260) | 305 | x~Beta(1174.74, 188.10) |  |  |  |  |
| Luengo-Fernandez et al. (2013), post-TIA | | 12 | 0.78 (s.d.= 0.260) | 305 | x~Beta(1174.74, 188.10) | AUC= 0.78 (0.011) | | N/a, see post-TIA control row | 0.89 (0.013) |
|  |  | 24 | 0.76 (s.d.= 0.260) | 173 | x~Beta(705.57, 125.06) |  |  |  |  |
|  |  | 60 | 0.80 (s.d.= 0.220) | 210 | x~Beta(1057.02, 151.65) |  |  |  |  |
| Luengo-Fernandez et al. (2013) ), non-CVD control for TIA | | 0 | 0.85 (s.d.= 0.220) | 270 | x~Beta(1093.69, 113.61) | AUC= 0.86 | | N/a | N/a |
|  |  | 1 | 0.85 (s.d.= 0.220) | 271 | x~Beta(1097.75, 114.03) |  |  |  |  |
|  |  | 6 | 0.86 (s.d.= 0.210) | 222 | x~Beta(933.81, 89.91) |  |  |  |  |
|  |  | 12 | 0.86 (s.d.= 0.200) | 260 | x~Beta(1206.02, 116.12) |  |  |  |  |
| Luengo-Fernandez et al. (2013), non-CVD control for post-TIA | | 12 | 0.86 (s.d.= 0.200) | 260 | x~Beta(1206.02, 116.12) | AUC= 0.87 | | N/a | N/a |
|  |  | 24 | 0.86 (s.d.= 0.200) | 146 | x~Beta(676.83, 65.17) |  |  |  |  |
|  |  | 60 | 0.89 (s.d.= 0.160) | 185 | x~Beta(1097.33, 81.34) |  |  |  |  |
| McCreanor et al. (2021), SA | Control (66.1, 76%) | 0 | 0.79 (s.d.= 0.220) | 89 | x~Beta(463.16, 70.28) | AUC= 0.82 (0.020) | | 0.86 | 0.95 (0.016) |
|  |  | 1.5 | 0.82 (s.d.= 0.200) | 89 | x~Beta(501.47, 63.84) |  |  |  |  |
|  |  | 12 | 0.82 (s.d.= 0.200) | 89 | x~Beta(501.47, 63.84) |  |  |  |  |
| Mejia et al. (2014), heart failure (70.6, 45%) | | 0 | 0.61 (s.e.= 0.018) | 260 | x~Beta(1099.59, 357.89) | Direct= 0.61 (0.018) | | 0.84 | 0.73 (0.021) |
| Monahan et al. (2017), heart failure (77.4, 50%) | | 0 | 0.62 (s.d.= 0.305) | 104 | x~Beta(393.83, 125.41) | Direct= 0.62 (0.030) | | 0.81 | 0.76 (0.037) |
| Munyombwe et al. (2020), MI (64.6, 75%) | | 0 | 0.72 (s.d.= 0.290) | 9263 | x~Beta(33404.53, 7118.17) | AUC= 0.76 (0.002) | | 0.86 | 0.88 (0.002) |
|  |  | 1 | 0.74 (s.d.= 0.260) | 6585 | x~Beta(28274.37, 5510.75) |  |  |  |  |
|  |  | 6 | 0.77 (s.d.= 0.260) | 5499 | x~Beta(21836.76, 3682.15) |  |  |  |  |
|  |  | 12 | 0.78 (s.d.= 0.260) | 5042 | x~Beta(19433.22, 3111.58) |  |  |  |  |
| Munyombwe et al. (2020), post-MI (65.1, 75%) | | 12 | 0.78 (s.d.= 0.260) | 5042 | x~Beta(19433.22, 3111.58) | Direct= 0.78 (0.004) | | 0.86 | 0.90 (0.004) |
| Nam et al. (2015), MI (61.1, 73%) | | 0 | 0.80 (s.e.= 0.018) | 174 | x~Beta(761.01, 108.56) | Direct= 0.80 (0.018) | | 0.87 | 0.92 (0.021) |
| Nam et al. (2015), post-MI (61.6, 73%) | | 12 | 0.80 (s.d.= 0.270) | 174* | x~Beta(581.08, 83.37) | Direct= 0.80 (0.021) | | 0.87 | 0.92 (0.024) |
| Phan et al. (2019), post-stroke | Men (70.1, 100%) | 12 | 0.75 (s.d.= 0.280) | 384 | x~Beta(1386.76, 257.95) | AUC= 0.76 | Pooled= 0.71 (0.015) | 0.83 | 0.85 (0.018) |
|  | Men (72.7, 100%) | 60 | 0.67 (s.d.= 0.900) | 328 | x~Beta(133.15, 34.76) |  |  |  |  |
|  | Women (67.9, 0%) | 12 | 0.76 (s.d.= 0.260) | 139 | x~Beta(566.73, 100.45) | AUC= 0.67 |  |  |  |
|  | Women (69.7, 0%) | 60 | 0.66 (s.d.= 0.310) | 130 | x~Beta(452.95, 122.81) |  |  |  |  |
| Pockett et al. (2018), unstable angina (69.2, 64%) | | 1 | 0.62 (s.d.= 0.320) | 535 | x~Beta(1829.39, 566.71) | AUC= 0.63 (0.009) | | 0.85 | 0.74 (0.011) |
|  |  | 6 | 0.64 (s.d.= 0.372) | 552 | x~Beta(1375.76, 405.69) |  |  |  |  |
|  |  | 12 | 0.63 (s.d.= 0.339) | 635 | x~Beta(1930.87, 593.99) |  |  |  |  |
| Pockett et al. (2018), post-unstable angina (70.1, 64%) | | 12 | 0.63 (s.d.= 0.339) | 635 | x~Beta(1930.87, 593.99) | Direct= 0.63 (0.013) | | 0.85 | 0.74 (0.016) |
| Pockett et al. (2018), MI (67.5, 70%) | | 1 | 0.69 (s.d.= 0.290) | 702 | x~Beta(2675.55, 645.97) | AUC= 0.70 (0.007) | | 0.86 | 0.82 (0.008) |
|  |  | 6 | 0.70 (s.d.= 0.309) | 733 | x~Beta(2409.79, 554.10) |  |  |  |  |
|  |  | 12 | 0.71 (s.d.= 0.322) | 817 | x~Beta(2446.14, 548.60) |  |  |  |  |
| Pockett et al. (2018), post-MI (68.4, 70%) | | 12 | 0.71 (s.d.= 0.322) | 817 | x~Beta(2446.14, 548.60) | Direct= 0.71 (0.011) | | 0.86 | 0.83 (0.013) |
| Roffe et al. (2018), stroke (72.5, 55%) | | 0 | 0.43 (s.e.= 0.010)* | 2407 | x~Beta(3600.35, 2004.10) | AUC= 0.43 (0.006) | | 0.84 | 0.51 (0.008) |
|  |  | 3 | 0.43 (s.e.= 0.010) | 2407 | x~Beta(3600.35, 2004.10) |  |  |  |  |
|  |  | 6 | 0.43 (s.e.= 0.013) | 2328 | x~Beta(2304.00, 1282.50) |  |  |  |  |
|  |  | 12 | 0.41 (s.e.= 0.013) | 2081 | x~Beta(2292.60, 1347.24) |  |  |  |  |
| Roffe et al. (2018), post-stroke (73.0, 55%) | | 12 | 0.41 (s.e.= 0.013) | 2081 | x~Beta(2292.60, 1347.24) | Direct= 0.41 (0.013) | | 0.84 | 0.49 (0.015) |
| Sandercock (2013), post-stroke (80.0, 49%) | | 18 | 0.50 (s.e.= 0.016) | 667 | x~Beta(1465.27, 665.79) | Direct= 0.50 (0.016) | | 0.79 | 0.63 (0.020) |
| Shawo et al. (2020), stroke | Intervention (72.5, 61%) | 1-12 (AUC) | 0.58 (s.e.= 0.020) | 235 | x~Beta(871.18, 311.66) | Unused | | N/a | N/a |
|  | Control (72.5, 58%) | 1-12 (AUC) | 0.52 (s.e.= 0.020) | 259 | x~Beta(896.52, 386.29) | Direct= 0.52 (0.021) | | 0.84 | 0.62 (0.025) |
|  | Intervention (73.5, 61%) | 12-24 (AUC) | 0.53 (s.e.= 0.023) | 235* | x~Beta(705.96, 295.20) | Unused | | N/a | N/a |
|  | Control (73.5, 58%) | 12-24 (AUC) | 0.48 (s.e.= 0.023) | 259* | x~Beta(713.16, 345.29) | Direct= 0.48 (0.023) | | 0.84 | 0.57 (0.027) |
| Squire et al. (2017), Post-heart failure (70.0, 73%) | | 0 | 0.60 (s.d.= 0.250) | 185 | x~Beta(1058.19, 354.50) | Direct= 0.60 (0.018) | | 0.85 | 0.70 (0.021) |
| Wallace et al. (2020), stroke | Treatment (50.0, 64%) | >1 | 0.62 (s.d.= 0.140) | 14 | x~Beta(250.20, 78.32) | Pooled= 0.63 (0.029) | | 0.89 | 0.71 (0.033) |
|  | Placebo (48.0, 71%) | >1 | 0.64 (s.d.= 0.170) | 14 | x~Beta(166.40, 49.07) |  |  |  |  |
| Walker et al. (2021), stable angina | Cardiovascular magnetic resonance (56.3, 53%) | 0 | 0.75 (s.d.= 0.235) | 481 | x~Beta(2456.12, 450.10) | AUC= 0.77 | Pooled= 0.75 (0.005) | 0.87 | 0.86 (0.006) |
|  |  | 6 | 0.77 (s.d.= 0.275) | 481* | x~Beta(1716.77, 293.30) |  |  |  |  |
|  |  | 12 | 0.77 (s.d.= 0.372) | 481* | x~Beta(923.11, 152.34) |  |  |  |  |
|  | Myocardial perfusion scintigraphy (56.3, 53%) | 0 | 0.74 (s.d.= 0.234) | 481 | x~Beta(2554.52, 500.17) | AUC= 0.75 |  |  |  |
|  |  | 6 | 0.76 (s.d.= 0.295) | 481* | x~Beta(1541.88, 277.79) |  |  |  |  |
|  |  | 12 | 0.75 (s.d.= 0.311) | 481* | x~Beta(1395.36, 255.16) |  |  |  |  |
|  | NICE (56.3, 53%) | 0 | 0.72 (s.d.= 0.266) | 240 | x~Beta(1032.78, 222.11) | AUC= 0.72 |  |  |  |
|  |  | 6 | 0.73 (s.d.= 0.283) | 240* | x~Beta(892.07, 183.03) |  |  |  |  |
|  |  | 12 | 0.71 (s.d.= 0.336) | 240* | x~Beta(652.71, 142.57) |  |  |  |  |
| Walker et al. (2021), post- stable angina | Cardiovascular magnetic resonance (56.3, 53%) | 12 | 0.77 (s.d.= 0.372) | 481 | x~Beta(923.11, 152.34) | AUC= 0.77 | Pooled= 0.75 (0.006) | 0.86 | 0.87 (0.007) |
|  |  | 24 | 0.78 (s.d.= 0.318) | 481* | x~Beta(1243.19, 200.86) |  |  |  |  |
|  |  | 36 | 0.76 (s.d.= 0.340) | 481* | x~Beta(1139.41, 198.69) |  |  |  |  |
|  | Myocardial perfusion scintigraphy (56.3, 53%) | 12 | 0.75 (s.d.= 0.311) | 481 | x~Beta(1395.36, 255.16) | AUC= 0.75 |  |  |  |
|  |  | 24 | 0.76 (s.d.= 0.348) | 481* | x~Beta(1103.19, 198.13) |  |  |  |  |
|  |  | 36 | 0.74 (s.d.= 0.303) | 481* | x~Beta(1522.27, 298.56) |  |  |  |  |
|  | NICE (56.3, 53%) | 12 | 0.71 (s.d.= 0.336) | 240 | x~Beta(652.71, 142.57) | AUC= 0.71 |  |  |  |
|  |  | 24 | 0.71 (s.d.= 0.350) | 240* | x~Beta(607.52, 136.27) |  |  |  |  |
|  |  | 36 | 0.71 (s.d.= 0.299) | 240* | x~Beta(825.66, 180.73) |  |  |  |  |
| *Unavailable, assumed equal to nearest time-point. **Sample from 1-((1-x)*1.594), with x as given. AUC: Area under the curve (utility against time). We used the lowest n across timepoints to calculate s.e. for AUC. | | | | | | | | | |

| **Studies in similar populations (when required)** | | | | | | | |
| --- | --- | --- | --- | --- | --- | --- | --- |
| **Study, disease \| Arm (mean age, % men)** | **Month** | **Raw utility** | **Number of participants** | **Modelled distribution**** | **Transformed utility (standard error)** | **Baseline utility (predicted unless stated)** | **Baseline utility adjustment (standard error)** |
| Vaidya et al. (2018), PAD (66.5, 66%) | 0 | 0.65 (s.d.= 0.22) | 204 | x~Beta(1434.98, 406.70) | Direct= 0.65 (0.015) | 0.76 | 0.76 (0.018) |

Appendix 5- (1) Reference information and (2) summary characteristics of studies reporting cardiovascular health state utility values

| **Citation** | **Reference** | **Link** |
| --- | --- | --- |
| [Abdin et al. (2015)](https://dx.doi.org/10.1007/s11136-014-0859-5) | Abdin, Edimansyah, Subramaniam, Mythily, Vaingankar, Janhavi Ajit, Luo, Nan. Population norms for the EQ-5D index scores using Singapore preference weights. Quality of life research : an international journal of quality of life aspects of treatment, care and rehabilitation 2015;24(6):1545-53. | https://dx.doi.org/10.1007/s11136-014-0859-5 |
| [Abdul et al. (2020)](https://dx.doi.org/10.1186/s12877-020-1453-z) | Abdul Aziz, Aznida Firzah, Mohd Nordin, Nor Azlin, Muhd Nur, Amrizal, Sulong, Saperi. The integrated care pathway for managing post stroke patients (iCaPPSÂ©) in public primary care Healthcentres in Malaysia: impact on quality adjusted life years (QALYs) and cost effectiveness analysis. BMC geriatrics 2020;20(1):70. | https://dx.doi.org/10.1186/s12877-020-1453-z |
| [Adey-Wakeling et al. (2016)](https://dx.doi.org/10.1097/PHM.0000000000000496) | Adey-Wakeling, Zoe, Liu, Enwu, Crotty, Maria, Leyden, James, Kleinig, Timothy, Anderson, Craig S. Hemiplegic Shoulder Pain Reduces Quality of Life After Acute Stroke: A Prospective Population-Based Study. American journal of physical medicine & rehabilitation 2016;95(10):758-63. | https://dx.doi.org/10.1097/PHM.0000000000000496 |
| [Adibe et al. (2013)](https://dx.doi.org/10.1016/j.vhri.2013.06.007) | Adibe, Maxwell O & Ukwe, Chinwe V. The Impact of Pharmaceutical Care Intervention on the Quality of Life of Nigerian Patients Receiving Treatment for Type 2 Diabetes. Value in health regional issues 2013;2(2):240-47. | https://dx.doi.org/10.1016/j.vhri.2013.06.007 |
| [Agren et al. (2013)](https://dx.doi.org/10.1111/j.1365-2702.2012.04246.x) | Agren, Susanna, S Evangelista, Lorraine, Davidson, Thomas. Cost-effectiveness of a nurse-led education and psychosocial programme for patients with chronic heart failure and their partners. Journal of clinical nursing 2013;22(15-16):2347-53. | https://dx.doi.org/10.1111/j.1365-2702.2012.04246.x |
| [Aguirre-Acevedo et al. (2020)](http://dx.doi.org/10.1016/j.rccar.2019.04.003) | Aguirre-Acevedo, Daniel Camilo, Vera-Giraldo, Claudia Y, Lugo-Agudelo, Luz Helena, Ortiz-Rangel, Sergio D, Rodriguez-Guevara, Camila, Vargas-Montoya, Diana M, et al. Validation of the Minnesota Living with Heart Failure questionnaire in patients with heart failure in Colombia. Revista Colombiana de Cardiologia 2020;27(6):564-72. | http://dx.doi.org/10.1016/j.rccar.2019.04.003 |
| [Agus et al. (2016)](https://dx.doi.org/10.1136/heartjnl-2015-308247) | Agus, A M, McKavanagh, P, Lusk, L, Verghis, R M, Walls, G M, Ball, P A, et al. The cost-effectiveness of cardiac computed tomography for patients with stable chest pain. Heart (British Cardiac Society) 2016;102(5):356-62. | https://dx.doi.org/10.1136/heartjnl-2015-308247 |
| [Ahimastos et al. (2013)](https://dx.doi.org/10.1001/jama.2012.216237) | Ahimastos, Anna A, Walker, Philip J, Askew, Christopher, Leicht, Anthony, Pappas, Elise, Blombery, Peter, et al. Effect of ramipril on walking times and quality of life among patients with peripheral artery disease and intermittent claudication: a randomized controlled trial. JAMA 2013;309(5):453-60. | https://dx.doi.org/10.1001/jama.2012.216237 |
| [Alberca et al. (2019)](http://dx.doi.org/10.1016/j.jstrokecerebrovasdis.2018.09.046) | Alberca, Sandra Bartolome, Ramirez-Moreno, Jose M, Munoz-Vega, Pedro, Peral-Pacheco Jose M. Health-Related Quality of Life and Fatigue After Transient Ischemic Attack and Minor Stroke. Journal of Stroke and Cerebrovascular Diseases 2019;28(2):276-84. | http://dx.doi.org/10.1016/j.jstrokecerebrovasdis.2018.09.046 |
| [Albuquerque de et al. (2020)](https://dx.doi.org/10.1186/s12955-020-01508-8) | Albuquerque de Almeida, Fernando, Al, Maiwenn J, Koymans, Ron, Riistama, Jarno, Pauws, Steffen. Impact of hospitalisation on health-related quality of life in patients with chronic heart failure. Health and quality of life outcomes 2020;18(1):262. | https://dx.doi.org/10.1186/s12955-020-01508-8 |
| [Ali et al. (2017)](https://dx.doi.org/10.1177/2396987316683780) | Ali, Myzoon, MacIsaac, Rachael, Quinn, Terence J, Bath, Philip M, Veenstra, David L, Xu, Yaping, et al. Dependency and health utilities in stroke: Data to inform cost-effectiveness analyses. European stroke journal 2017;2(1):70-76. | https://dx.doi.org/10.1177/2396987316683780 |
| [Alva et al. (2014)](https://dx.doi.org/10.1002/hec.2930) | Alva, Maria, Gray, Alastair, Mihaylova, Borislava. The effect of diabetes complications on health-related quality of life: the importance of longitudinal data to address patient heterogeneity. Health economics 2014;23(4):487-500. | https://dx.doi.org/10.1002/hec.2930 |
| [Alvarez-Sabin et al. (2016)](https://dx.doi.org/10.3390/ijms17030390) | Alvarez-Sabin, Jose, Santamarina, Estevo, Maisterra, Olga, Jacas, Carlos, Molina, Carlos. Long-Term Treatment with Citicoline Prevents Cognitive Decline and Predicts a Better Quality of Life after a First Ischemic Stroke. International journal of molecular sciences 2016;17(3):390. | https://dx.doi.org/10.3390/ijms17030390 |
| [Ambrosy et al. (2016)](https://dx.doi.org/10.1002/ejhf.420) | Ambrosy, Andrew P, Hernandez, Adrian F, Armstrong, Paul W, Butler, Javed, Dunning, Allison, Ezekowitz, Justin A, et al. The clinical course of health status and association with outcomes in patients hospitalized for heart failure: insights from ASCEND-HF. European journal of heart failure 2016;18(3):306-13. | https://dx.doi.org/10.1002/ejhf.420 |
| [Ambrosy et al. (2017)](https://dx.doi.org/10.1016/j.ahj.2016.12.017) | Ambrosy, Andrew P, Cerbin, Lukasz P, DeVore, Adam D, Greene, Stephen J, Kraus, William E, O'Connor, Christopher M, et al. Aerobic exercise training and general health status in ambulatory heart failure patients with a reduced ejection fraction-Findings from the Heart Failure and A Controlled Trial Investigating Outcomes of Exercise Training (HF-ACTION)trial. American heart journal 2017;186():130-38. | https://dx.doi.org/10.1016/j.ahj.2016.12.017 |
| [Amer et al. (2013)](https://dx.doi.org/10.1111/j.1447-0594.2012.00928.x) | Amer, Moatassem S, Alsadany, Mohamad A, Tolba, Mohammad F. Quality of life in elderly diabetic patients with peripheral arterial disease. Geriatrics & gerontology international 2013;13(2):443-50. | https://dx.doi.org/10.1111/j.1447-0594.2012.00928.x |
| [Andayani et al. (2020)](http://dx.doi.org/10.29090/psa.2020.03.019.0040) | Andayani, Tri Murti, Kristina, Susi Ari, Endarti, Dwi, Wahyuni, Septiana Tri. Health utility score in type 2 diabetes mellitus. Pharmaceutical Sciences Asia 2020;47(3):246-52. | http://dx.doi.org/10.29090/psa.2020.03.019.0040 |
| [Andrew et al. (2016)](https://dx.doi.org/10.1007/s11136-016-1234-5) | Andrew, N E, Kilkenny, M F, Lannin, N A. Is health-related quality of life between 90 and 180 days following stroke associated with long-term unmet needs?. Quality of life research : an international journal of quality of life aspects of treatment, care and rehabilitation 2016;25(8):2053-62. | https://dx.doi.org/10.1007/s11136-016-1234-5 |
| [Ankolekar et al. (2014)](https://dx.doi.org/10.1016/j.jstrokecerebrovasdis.2014.04.022) | Ankolekar, Sandeep, Renton, Cheryl, Sare, Gillian, Ellender, Sharon, Sprigg, Nikola, Wardlaw, Joanna M, Bath, Philip M W. Relationship between poststroke cognition, baseline factors, and functional outcome: data from 'efficacy of nitric oxide in stroke' trial. Journal of stroke and cerebrovascular diseases : the official journal of National Stroke Association 2014;23(7):1821-29. | https://dx.doi.org/10.1016/j.jstrokecerebrovasdis.2014.04.022 |
| [Anon. (2013)](http://dx.doi.org/10.1016/j.jchf.2012.08.002) | Anon. Cost-effectiveness of N-terminal pro-b-type natriuretic-guided therapy in elderly heart failure patients. Results from TIME-CHF (Trial of intensified versus standard medical therapy in elderly patients with congestive heart failure). JACC: Heart Failure 2013;1(1):64-71. | http://dx.doi.org/10.1016/j.jchf.2012.08.002 |
| [Appau et al. (2019)](https://dx.doi.org/10.1177/0269215519834064) | Appau, Adriana, Lencucha, Raphael, Finch, Lois. Further validation of the Preference-Based Stroke Index three months after stroke. Clinical rehabilitation 2019;33(7):1214-20. | https://dx.doi.org/10.1177/0269215519834064 |
| [Aprile et al. (2015)](http://ovidsp.ovid.com/ovidweb.cgi?T=JS&PAGE=reference&D=med12&NEWS=N&AN=25739508) | Aprile, I, Briani, C, Pazzaglia, C, Cecchi, F, Negrini, S, Padua, L. Pain in stroke patients: characteristics and impact on the rehabilitation treatment. A multicenter cross-sectional study. European journal of physical and rehabilitation medicine 2015;51(6):725-36. | http://ovidsp.ovid.com/ovidweb.cgi?T=JS&PAGE=reference&D=med12&NEWS=N&AN=25739508 |
| [Arrospide et al. (2019)](https://dx.doi.org/10.1186/s12955-019-1134-9) | Arrospide, Arantzazu, Machon, Monica, Ramos-Goni, Juan M, Ibarrondo, Oliver. Inequalities in health-related quality of life according to age, gender, educational level, social class, body mass index and chronic diseases using the Spanish value set for Euroquol 5D-5L questionnaire. Health and quality of life outcomes 2019;17(1):69. | https://dx.doi.org/10.1186/s12955-019-1134-9 |
| [Arwert et al. (2016)](https://dx.doi.org/10.1016/j.apmr.2015.09.018) | Arwert, Henk J, Keizer, Saskia, Kromme, Cornelis H, Vliet Vlieland, Thea P. Validity of the Michigan Hand Outcomes Questionnaire in Patients With Stroke. Archives of physical medicine and rehabilitation 2016;97(2):238-44. | https://dx.doi.org/10.1016/j.apmr.2015.09.018 |
| [Arwert et al. (2017)](https://dx.doi.org/10.1007/s10926-016-9651-4) | Arwert, H J, Schults, M, Meesters, J J L, Wolterbeek, R, Boiten, J. Return to Work 2-5 Years After Stroke: A Cross Sectional Study in a Hospital-Based Population. Journal of occupational rehabilitation 2017;27(2):239-46. | https://dx.doi.org/10.1007/s10926-016-9651-4 |
| [Ayis et al. (2015)](https://dx.doi.org/10.1136/bmjopen-2014-007101) | Ayis, Salma, Wellwood, Ian, Rudd, Anthony G, McKevitt, Christopher, Parkin, David. Variations in Health-Related Quality of Life (HRQoL) and survival 1 year after stroke: five European population-based registers. BMJ open 2015;5(6):e007101. | https://dx.doi.org/10.1136/bmjopen-2014-007101 |
| [Azmi et al. (2015)](https://dx.doi.org/10.1016/j.vhri.2015.03.015) | Azmi, Soraya, Goh, Adrian, Fong, Alan. Quality of life among Patients with Acute Coronary Syndrome in Malaysia. Value in health regional issues 2015;6():80-83. | https://dx.doi.org/10.1016/j.vhri.2015.03.015 |
| [Babber et al. (2020)](https://dx.doi.org/10.1002/bjs.11398) | Babber, A, Ravikumar, R, Onida, S, Lane, T R A. Effect of footplate neuromuscular electrical stimulation on functional and quality-of-life parameters in patients with peripheral artery disease: pilot, and subsequent randomized clinical trial. The British journal of surgery 2020;107(4):355-63. | https://dx.doi.org/10.1002/bjs.11398 |
| [Barclay & (2014)](https://dx.doi.org/10.1016/j.jclinepi.2013.12.003) | Barclay, Ruth. Response shift recalibration and reprioritization in health-related quality of life was identified prospectively in older men with and without stroke. Journal of clinical epidemiology 2014;67(5):500-07. | https://dx.doi.org/10.1016/j.jclinepi.2013.12.003 |
| [Bath et al. (2017)](https://dx.doi.org/10.1371/journal.pone.0164608) | Bath, Philip M, Scutt, Polly, Blackburn, Daniel J, Ankolekar, Sandeep, Krishnan, Kailash, Ballard, Clive, et al. Intensive versus Guideline Blood Pressure and Lipid Lowering in Patients with Previous Stroke: Main Results from the Pilot 'Prevention of Decline in Cognition after Stroke Trial' (PODCAST) Randomised Controlled Trial. PloS one 2017;12(1):e0164608. | https://dx.doi.org/10.1371/journal.pone.0164608 |
| [Beinotti et al. (2013)](https://dx.doi.org/10.1310/tsr2003-226) | Beinotti, Fernanda, Christofoletti, Gustavo, Correia, Nilzete. Effects of horseback riding therapy on quality of life in patients post stroke. Topics in stroke rehabilitation 2013;20(3):226-32. | https://dx.doi.org/10.1310/tsr2003-226 |
| [Benda et al. (2015)](https://dx.doi.org/10.1371/journal.pone.0141256) | Benda, Nathalie M M, Seeger, Joost P H, Stevens, Guus G C F, Hijmans-Kersten, Bregina T P, van Dijk, Arie P J, Bellersen, Louise, et al. Effects of High-Intensity Interval Training versus Continuous Training on Physical Fitness, Cardiovascular Function and Quality of Life in Heart Failure Patients. PloS one 2015;10(10):e0141256. | https://dx.doi.org/10.1371/journal.pone.0141256 |
| [Berg et al. (2015)](http://dx.doi.org/10.1016/j.jval.2015.02.003) | Berg, Jenny, Lindgren, Peter, Mejhert, Marit, Edner, Magnus, Dahlstrom, Ulf. Determinants of Utility Based on the EuroQol Five-Dimensional Questionnaire in Patients with Chronic Heart Failure and Their Change Over Time: Results from the Swedish Heart Failure Registry. Value in Health 2015;18(4):439-48. | http://dx.doi.org/10.1016/j.jval.2015.02.003 |
| [Berg et al. (2017)](https://dx.doi.org/10.1016/j.jpsychores.2017.01.003) | Berg, Selina Kikkenborg, Rasmussen, Trine Bernholdt, Thrysoee, Lars, Lauberg, Astrid, Borregaard, Britt, Christensen, Anne Vinggaard, et al. DenHeart: Differences in physical and mental health across cardiac diagnoses at hospital discharge. Journal of psychosomatic research 2017;94():1-9. | https://dx.doi.org/10.1016/j.jpsychores.2017.01.003 |
| [Blum & (2014)](https://dx.doi.org/10.1016/j.cardfail.2014.04.016) | Blum, Kay. The effect of a randomized trial of home telemonitoring on medical costs, 30-day readmissions, mortality, and health-related quality of life in a cohort of community-dwelling heart failure patients. Journal of cardiac failure 2014;20(7):513-21. | https://dx.doi.org/10.1016/j.cardfail.2014.04.016 |
| [Boczor et al. (2019)](http://dx.doi.org/10.1186/s12889-019-7623-2) | Boczor, Sigrid, Eisele, Marion, Blozik, Eva, Scherer, Martin, Daubmann Sigrid. Quality of life assessment in patients with heart failure: validity of the German version of the generic EQ-5D-5LTM. BMC public health 2019;19(1):1464. | http://dx.doi.org/10.1186/s12889-019-7623-2 |
| [Bohmer et al. (2014)](https://dx.doi.org/10.3109/14017431.2014.923581) | Bohmer, Ellen, Kristiansen, Ivar S, Arnesen, Harald. Health-related quality of life after myocardial infarction, does choice of method make a difference?. Scandinavian cardiovascular journal : SCJ 2014;48(4):216-22. | https://dx.doi.org/10.3109/14017431.2014.923581 |
| [Bosma et al. (2014)](https://dx.doi.org/10.1177/0284185113496560) | Bosma, Jan, Dijksman, Lea M, Lam, Kayan, Wisselink, Willem, van Swijndregt, Alexander D Montauban. The costs and effects of contrast-enhanced magnetic resonance angiography and digital substraction angiography on quality of life in patients with peripheral arterial disease. Acta radiologica (Stockholm, Sweden : 1987) 2014;55(3):279-86. | https://dx.doi.org/10.1177/0284185113496560 |
| [Briggs et al. (2017)](https://dx.doi.org/10.1016/j.diabres.2016.12.019) | Briggs, Andrew H, Bhatt, Deepak L, Scirica, Benjamin M, Raz, Itamar, Johnston, Karissa M, Szabo, Shelagh M, et al. Health-related quality-of-life implications of cardiovascular events in individuals with type 2 diabetes mellitus: A subanalysis from the Saxagliptin Assessment of Vascular Outcomes Recorded in Patients with Diabetes Mellitus (SAVOR)-TIMI 53 trial. Diabetes research and clinical practice 2017;130():24-33. | https://dx.doi.org/10.1016/j.diabres.2016.12.019 |
| [Burton et al. (2014)](https://dx.doi.org/10.1186/1472-6963-14-63) | Burton, Christopher R, Fargher, Emily, Plumpton, Catrin, Roberts, Gwerfyl W, Owen, Heledd. Investigating preferences for support with life after stroke: a discrete choice experiment. BMC health services research 2014;14():63. | https://dx.doi.org/10.1186/1472-6963-14-63 |
| [Bushnell et al. (2014)](https://dx.doi.org/10.1212/WNL.0000000000000208) | Bushnell, Cheryl D, Reeves, Mathew J, Zhao, Xin, Pan, Wenqin, Prvu-Bettger, Janet, Zimmer, Louise, Olson, Daiwai. Sex differences in quality of life after ischemic stroke. Neurology 2014;82(11):922-31. | https://dx.doi.org/10.1212/WNL.0000000000000208 |
| [Cadilhac et al. (2017)](https://dx.doi.org/10.1161/STROKEAHA.116.015714) | Cadilhac, Dominique A, Andrew, Nadine E, Lannin, Natasha A, Middleton, Sandy, Levi, Christopher R, Dewey, Helen M, et al. Quality of Acute Care and Long-Term Quality of Life and Survival: The Australian Stroke Clinical Registry. Stroke 2017;48(4):1026-32. | https://dx.doi.org/10.1161/STROKEAHA.116.015714 |
| [Campbell et al. (2018)](http://dx.doi.org/10.1002/ejhf.1240) | Campbell, Ross T, Jhund, Pardeep S, Wright, Ann, McMurray, John J V, Petrie, Mark C, Gardner, Roy S, et al. Which patients with heart failure should receive specialist palliative care? European Journal of Heart Failure 2018;20(9):1338-47. | http://dx.doi.org/10.1002/ejhf.1240 |
| [Campo et al. (2020)](https://dx.doi.org/10.1136/heartjnl-2019-316349) | Campo, Gianluca, Tonet, Elisabetta, Chiaranda, Giorgio, Sella, Gianluigi, Maietti, Elisa, Bugani, Giulia, et al. Exercise intervention improves quality of life in older adults after myocardial infarction: randomised clinical trial. Heart (British Cardiac Society) 2020;106(21):1658-64. | https://dx.doi.org/10.1136/heartjnl-2019-316349 |
| [Cano-Manas et al. (2020)](http://dx.doi.org/10.1155/2020/5480315) | Cano-Manas, Maria Jose, Rodriguez Hernandez, Javier, Collado-Vazquez, Susana, Cano-De-La-Cuerda, Roberto, Munoz Villena Susana, ORCID: http://orcid.org/0000-0002-3957-0057 AO - Cano-De-La-Cuerda, Roberto. Effects of Video-Game Based Therapy on Balance, Postural Control, Functionality, and Quality of Life of Patients with Subacute Stroke: A Randomized Controlled Trial. Journal of Healthcare Engineering 2020;2020():5480315. | http://dx.doi.org/10.1155/2020/5480315 |
| [Chandrasekaran et al. (2020)](http://dx.doi.org/10.1016/j.jacc.2020.01.050) | Chandrasekaran, Ambalam M, Singh, Kalpana, Singh, Kavita, Devarajan, Raji, Kondal, Dimple, Soni, Divya, et al. Yoga-Based Cardiac Rehabilitation After Acute Myocardial Infarction: A Randomized Trial. Journal of the American College of Cardiology 2020;75(13):1551-61. | http://dx.doi.org/10.1016/j.jacc.2020.01.050 |
| [Chandrasekhar et al. (2018)](http://dx.doi.org/10.1016/j.cegh.2018.05.001) | Chandrasekhar, Dilip, Pradeep, Anuja, Geoji, Asha Susan, George, Athira Elezebath, Athira, V. Impact of intensified pharmaceutical care on health related quality of life in patients with stroke in a tertiary care hospital. Clinical Epidemiology and Global Health 2018;6(4):198-202. | http://dx.doi.org/10.1016/j.cegh.2018.05.001 |
| [Chang et al. (2016)](https://dx.doi.org/10.1007/s00415-016-8119-y) | Chang, Won Hyuk, Sohn, Min Kyun, Lee, Jongmin, Kim, Deog Young, Lee, Sam-Gyu, Shin, Yong-Il, et al. Predictors of functional level and quality of life at 6 months after a first-ever stroke: the KOSCO study. Journal of neurology 2016;263(6):1166-77. | https://dx.doi.org/10.1007/s00415-016-8119-y |
| [Chavanon et al. (2017)](https://dx.doi.org/10.1007/s00392-017-1101-6) | Chavanon, Mira-Lynn, Inkrot, Simone, Zelenak, Christine, Tahirovic, Elvis, Stanojevic, Dragana, Apostolovic, Svetlana, et al. Regional differences in health-related quality of life in elderly heart failure patients: results from the CIBIS-ELD trial. Clinical research in cardiology : official journal of the German Cardiac Society 2017;106(8):645-55. | https://dx.doi.org/10.1007/s00392-017-1101-6 |
| [Chen et al. (2015)](https://dx.doi.org/10.1016/j.jstrokecerebrovasdis.2015.02.002) | Chen, Chen-Ling, Chang, Ken-Jie, Wu, Pei-Ying, Chi, Chun-Han, Chang, Shin-Tsu. Comparison of the Effects between Isokinetic and Isotonic Strength Training in Subacute Stroke Patients. Journal of stroke and cerebrovascular diseases : the official journal of National Stroke Association 2015;24(6):1317-23. | https://dx.doi.org/10.1016/j.jstrokecerebrovasdis.2015.02.002 |
| [Chen et al. (2016)](https://dx.doi.org/10.1007/s11136-015-1196-z) | Chen, Poyu, Lin, Keh-Chung, Liing, Rong-Jiuan, Wu, Ching-Yi, Chen, Chia-Ling. Validity, responsiveness, and minimal clinically important difference of EQ-5D-5L in stroke patients undergoing rehabilitation. Quality of life research : an international journal of quality of life aspects of treatment, care and rehabilitation 2016;25(6):1585-96. | https://dx.doi.org/10.1007/s11136-015-1196-z |
| [Chen et al. (2019)](https://dx.doi.org/10.1097/MD.0000000000015130) | Chen, Qi, Cao, Chunni, Gong, Li. Health related quality of life in stroke patients and risk factors associated with patients for return to work. Medicine 2019;98(16):e15130. | https://dx.doi.org/10.1097/MD.0000000000015130 |
| [Chen et al. (2020)](https://dx.doi.org/10.1161/STROKEAHA.119.027639) | Chen, Xiaoying, Wang, Xia, Delcourt, Candice, Li, Jingwei, Arima, Hisatomi, Hackett, Maree L, et al. Ethnicity and Other Determinants of Quality of Functional Outcome in Acute Ischemic Stroke: The ENCHANTED Trial. Stroke 2020;51(2):588-93. | https://dx.doi.org/10.1161/STROKEAHA.119.027639 |
| [Cheung et al. (2019)](https://dx.doi.org/10.1007/s11136-019-02254-1) | Cheung, Yin Bun, Tan, Hui Xing, Luo, Nan, Wee, Hwee Lin. Mapping the Shah-modified Barthel Index to the Health Utility Index Mark III by the Mean Rank Method. Quality of life research : an international journal of quality of life aspects of treatment, care and rehabilitation 2019;28(12):3177-85. | https://dx.doi.org/10.1007/s11136-019-02254-1 |
| [Chimatiro et al. (2018)](https://dx.doi.org/10.4314/mmj.v30i3.4) | Chimatiro, George Lameck & Rhoda, Anthea J. Stroke patients' outcomes and satisfaction with care at discharge from four referral hospitals in Malawi: A cross-sectional descriptive study in limited resource. Malawi medical journal : the journal of Medical Association of Malawi 2018;30(3):152-58. | https://dx.doi.org/10.4314/mmj.v30i3.4 |
| [Cohen et al. (2018)](https://dx.doi.org/10.1016/j.apmr.2017.12.007) | Cohen, Joshua W, Ivanova, Tanya D, Brouwer, Brenda, Miller, Kimberly J, Bryant, Dianne. Do Performance Measures of Strength, Balance, and Mobility Predict Quality of Life and Community Reintegration After Stroke?. Archives of physical medicine and rehabilitation 2018;99(4):713-19. | https://dx.doi.org/10.1016/j.apmr.2017.12.007 |
| [Comin-Colet et al. (2016)](https://dx.doi.org/10.1016/j.rec.2015.07.030) | Comin-Colet, Josep, Anguita, Manuel, Formiga, Francesc, Almenar, Luis, Crespo-Leiro, Maria G, Manzano, Luis, et al. Health-related Quality of Life of Patients With Chronic Systolic Heart Failure in Spain: Results of the VIDA-IC Study. Revista espanola de cardiologia (English ed.) 2016;69(3):256-71. | https://dx.doi.org/10.1016/j.rec.2015.07.030 |
| [Cui et al. (2013)](http://ovidsp.ovid.com/ovidweb.cgi?T=JS&PAGE=reference&D=med10&NEWS=N&AN=24359716) | Cui, Yang, Doupe, Malcolm, Katz, Alan, Nyhof, Paul. Economic evaluation of Manitoba Health Lines in the management of congestive heart failure. Healthcare policy = Politiques de sante 2013;9(2):36-50. | http://ovidsp.ovid.com/ovidweb.cgi?T=JS&PAGE=reference&D=med10&NEWS=N&AN=24359716 |
| [Dagner et al. (2019)](https://dx.doi.org/10.1177/1474515118783936) | Dagner, Viveka & Clausson, Eva K. Prescribed physical activity maintenance following exercise based cardiac rehabilitation: factors predicting low physical activity. European journal of cardiovascular nursing : journal of the Working Group on Cardiovascular Nursing of the European Society of Cardiology 2019;18(1):21-27. | https://dx.doi.org/10.1177/1474515118783936 |
| [D'Aniello et al. (2014)](https://dx.doi.org/10.1016/j.jns.2014.01.005) | D'Aniello, Guido Edoardo, Scarpina, Federica, Mauro, Alessandro, Mori, Ileana, Castelnuovo, Gianluca, Bigoni, Matteo, Baudo, Silvia. Characteristics of anxiety and psychological well-being in chronic post-stroke patients. Journal of the neurological sciences 2014;338(1-2):191-96. | https://dx.doi.org/10.1016/j.jns.2014.01.005 |
| [Dankner et al. (2016)](http://ovidsp.ovid.com/ovidweb.cgi?T=JS&PAGE=reference&D=med13&NEWS=N&AN=27548022) | Dankner, Rachel, Bachner, Yaacov G, Ginsberg, Gary, Ziv, Arnona, Ben David, Hadar, Litmanovitch-Goldstein, Dalit, et al. Correlates of well-being among caregivers of long-term community-dwelling stroke survivors. International journal of rehabilitation research. Internationale Zeitschrift fur Rehabilitationsforschung. Revue internationale de recherches de readaptation 2016;39(4):326-30. | http://ovidsp.ovid.com/ovidweb.cgi?T=JS&PAGE=reference&D=med13&NEWS=N&AN=27548022 |
| [Davalos et al. (2017)](https://dx.doi.org/10.1016/S1474-4422(17)30047-9) | Davalos, Antoni, Cobo, Erik, Molina, Carlos A, Chamorro, Angel, de Miquel, M Angeles, Roman, Luis San, et al. Safety and efficacy of thrombectomy in acute ischaemic stroke (REVASCAT): 1-year follow-up of a randomised open-label trial. The Lancet. Neurology 2017;16(5):369-76. | https://dx.doi.org/10.1016/S1474-4422(17)30047-9 |
| [Davies et al. (2015)](https://dx.doi.org/10.1186/s12955-015-0266-9) | Davies, Evan W, Matza, Louis S, Worth, Gavin, Feeny, David H, Kostelec, Jacqueline, Soroka, Steven, et al. Health state utilities associated with major clinical events in the context of secondary hyperparathyroidism and chronic kidney disease requiring dialysis. Health and quality of life outcomes 2015;13():90. | https://dx.doi.org/10.1186/s12955-015-0266-9 |
| [De et al. (2017)](https://dx.doi.org/10.1080/09638288.2016.1200676) | De Wit, Liesbet, Theuns, Peter, Dejaeger, Eddy, Devos, Stefanie, Gantenbein, Andreas R, Kerckhofs, Eric, et al. Long-term impact of stroke on patients' health-related quality of life. Disability and rehabilitation 2017;39(14):1435-40. | https://dx.doi.org/10.1080/09638288.2016.1200676 |
| [De et al. (2018)](https://dx.doi.org/10.1371/journal.pone.0199770) | De Luca, Leonardo, Temporelli, Pier Luigi, Lucci, Donata, Colivicchi, Furio, Calabro, Paolo, Riccio, Carmine, et al. Characteristics, treatment and quality of life of stable coronary artery disease patients with or without angina: Insights from the START study. PloS one 2018;13(7):e0199770. | https://dx.doi.org/10.1371/journal.pone.0199770 |
| [de et al. (2021)](https://dx.doi.org/10.23736/S1973-9087.21.06335-8) | de Graaf, Joris A, Visser-Meily, Johanna M, Schepers, Vera P, Baars, Annette, Kappelle, L Jaap, Passier, Patricia E, et al. Comparison between EQ-5D-5L and PROMIS-10 to evaluate health-related quality of life 3 months after stroke: a cross-sectional multicenter study. European journal of physical and rehabilitation medicine 2021;():n. pag.. | https://dx.doi.org/10.23736/S1973-9087.21.06335-8 |
| [Dewilde et al. (2019)](https://dx.doi.org/10.1186/s12955-018-1069-6) | Dewilde, Sarah, Annemans, Lieven, Lloyd, Andrew, Peeters, Andre, Hemelsoet, Dimitri, Vandermeeren, Yves, et al. The combined impact of dependency on caregivers, disability, and coping strategy on quality of life after ischemic stroke. Health and quality of life outcomes 2019;17(1):31. | https://dx.doi.org/10.1186/s12955-018-1069-6 |
| [Diekmann et al. (2019)](https://dx.doi.org/10.1080/03007995.2019.1646000) | Diekmann, Sandra, Horster, Laura, Evers, Silvia, Hiligsmann, Mickael, Gelbrich, Gotz, Groschel, Klaus, et al. Economic evaluation of prolonged and enhanced ECG Holter monitoring in acute ischemic stroke patients. Current medical research and opinion 2019;35(11):1859-66. | https://dx.doi.org/10.1080/03007995.2019.1646000 |
| [Dijkland et al. (2018)](http://dx.doi.org/10.1161/STROKEAHA.117.020194) | Dijkland, Simone A, Voormolen, Daphne C, Polinder, Suzanne, Haagsma, Juanita A, Nieboer, Daan, Lingsma, Hester F, et al. Utility-weighted modifed rankin scale as primary outcome in stroke trials a simulation study. Stroke 2018;49(4):965-71. | http://dx.doi.org/10.1161/STROKEAHA.117.020194 |
| [Ding et al. (2019)](https://dx.doi.org/10.1161/JAHA.118.010988) | Ding, Qinglan, Funk, Marjorie, Spatz, Erica S, Whittemore, Robin, Lin, Haiqun, Lipska, Kasia J, et al. Association of Diabetes Mellitus With Health Status Outcomes in Young Women and Men After Acute Myocardial Infarction: Results From the VIRGO Study. Journal of the American Heart Association 2019;8(17):e010988. | https://dx.doi.org/10.1161/JAHA.118.010988 |
| [do Nascimento et al. (2015)](http://dx.doi.org/10.1590/1677-5449.20140043) | do Nascimento Sales, Ana Tereza, Silva, Ana Gabriela Camara Batista, Ribeiro, Cibele Teresinha Dias, Fregonezi, Guilherme Augusto de Freitas, Dourado-Junior, Mario Emilio Teixeira, Sousa, Andre Gustavo Pires. Identification of peripheral arterial disease in diabetic patients and its association with quality of life, physical activity and body composition. Jornal Vascular Brasileiro 2015;14(1):46-54. | http://dx.doi.org/10.1590/1677-5449.20140043 |
| [Dohl et al. (2020)](https://dx.doi.org/10.1186/s12913-020-05158-w) | Dohl, Oystein, Halsteinli, Vidar, Askim, Torunn, Gunnes, Mari, Ihle-Hansen, Hege, Indredavik, Bent, et al. Factors contributing to post-stroke health care utilization and costs, secondary results from the life after stroke (LAST) study. BMC health services research 2020;20(1):288. | https://dx.doi.org/10.1186/s12913-020-05158-w |
| [Dong et al. (2017)](https://dx.doi.org/10.1007/s40520-016-0614-6) | Dong, Aishu, Chen, Sisi, Zhu, Lianlian, Shi, Lingmin, Cai, Yueli, Zeng, Jingni. The reliability and validity of Chinese version of SF36 v2 in aging patients with chronic heart failure. Aging clinical and experimental research 2017;29(4):685-93. | https://dx.doi.org/10.1007/s40520-016-0614-6 |
| [Dreyer et al. (2016)](https://dx.doi.org/10.1177/2048872615568967) | Dreyer, Rachel P, Smolderen, Kim G, Strait, Kelly M, Beltrame, John F, Lichtman, Judith H, Lorenze, Nancy P, et al. Gender differences in pre-event health status of young patients with acute myocardial infarction: A VIRGO study analysis. European heart journal. Acute cardiovascular care 2016;5(1):43-54. | https://dx.doi.org/10.1177/2048872615568967 |
| [Dreyer et al. (2019)](https://dx.doi.org/10.1177/2048872618803726) | Dreyer, Rachel P, Zheng, Xin, Xu, Xiao, Liu, Shuling, Li, Jing, Ding, Qinglan, et al. Sex differences in health outcomes at one year following acute myocardial infarction: A report from the China Patient-Centered Evaluative Assessment of Cardiac Events prospective acute myocardial infarction study. European heart journal. Acute cardiovascular care 2019;8(3):273-82. | https://dx.doi.org/10.1177/2048872618803726 |
| [Dundar et al. (2014)](https://dx.doi.org/10.1310/tsr2106-453) | Dundar, U, Toktas, H, Solak, O, Ulasli, A M. A comparative study of conventional physiotherapy versus robotic training combined with physiotherapy in patients with stroke. Topics in stroke rehabilitation 2014;21(6):453-61. | https://dx.doi.org/10.1310/tsr2106-453 |
| [Dzubur et al. (2016)](https://dx.doi.org/10.5455/medarh.2016.70.419-424) | Dzubur, Alen, Mekic, Mevludin, Pesto, Senad. Echocardiographic Parameters as Life Quality Predictors in Patients After Myocardial Infarction Treated with Different Methods. Medical archives (Sarajevo, Bosnia and Herzegovina) 2016;70(6):419-24. | https://dx.doi.org/10.5455/medarh.2016.70.419-424 |
| [Edelmann et al. (2015)](https://dx.doi.org/10.1002/ejhf.203) | Edelmann, Frank, Holzendorf, Volker, Wachter, Rolf, Nolte, Kathleen, Schmidt, Albrecht G, Kraigher-Krainer, Elisabeth, et al. Galectin-3 in patients with heart failure with preserved ejection fraction: results from the Aldo-DHF trial. European journal of heart failure 2015;17(2):214-23. | https://dx.doi.org/10.1002/ejhf.203 |
| [Em et al. (2015)](http://dx.doi.org/10.5152/tftrd.2015.80090) | Em, Serda, Bozkurt, Mehtap, Karakoc, Mehmet, Caglayan, Mehmet, Akdeniz, Dicle, Oktayoglu, Pelin, Nas, Kemal. Determining quality of life and associated factors in patients with stroke. Turkiye Fiziksel Tip ve Rehabilitasyon Dergisi 2015;61(2):148-54. | http://dx.doi.org/10.5152/tftrd.2015.80090 |
| [Eriksson et al. (2013)](https://dx.doi.org/10.1111/j.1471-6712.2012.01032.x) | Eriksson, Monica, Asplund, Kenneth, Hochwalder, Jacek. Changes in hope and health-related quality of life in couples following acute myocardial infarction: a quantitative longitudinal study. Scandinavian journal of caring sciences 2013;27(2):295-302. | https://dx.doi.org/10.1111/j.1471-6712.2012.01032.x |
| [Erta & (2018)](http://dx.doi.org/10.5543/tkda.2017.66724) | Erta, Fatih Sinan. Long-term follow-up of antithrombotic management patterns in acute coronary syndrome patients. Turk Kardiyoloji Dernegi Arsivi 2018;46(3):175-83. | http://dx.doi.org/10.5543/tkda.2017.66724 |
| [Ezeofor et al. (2021)](https://dx.doi.org/10.1371/journal.pone.0244851) | Ezeofor, Victory 'Segun, Bray, Nathan, Bryning, Lucy, Hashmi, Farina, Hoel, Henrik, Parker, Daniel. Economic model to examine the cost-effectiveness of FlowOx home therapy compared to standard care in patients with peripheral artery disease. PloS one 2021;16(1):e0244851. | https://dx.doi.org/10.1371/journal.pone.0244851 |
| [Fakhry et al. (2015)](https://dx.doi.org/10.1001/jama.2015.14851) | Fakhry, Farzin, Spronk, Sandra, van der Laan, Lijckle, Wever, Jan J, Teijink, Joep A W, Hoffmann, Wolter H, et al. Endovascular Revascularization and Supervised Exercise for Peripheral Artery Disease and Intermittent Claudication: A Randomized Clinical Trial. JAMA 2015;314(18):1936-44. | https://dx.doi.org/10.1001/jama.2015.14851 |
| [Farkas et al. (2020)](https://dx.doi.org/10.1024/0301-1526/a000845) | Farkas, Katalin & Kolossvary, Endre. Simple assessment of quality of life and lower limb functional capacity during cilostazol treatment - results of the SHort-tERm cIlostazol eFFicacy and quality of life (SHERIFF) study. VASA. Zeitschrift fur Gefasskrankheiten 2020;49(3):235-42. | https://dx.doi.org/10.1024/0301-1526/a000845 |
| [Farndon et al. (2018)](https://dx.doi.org/10.1186/s13047-018-0269-y) | Farndon, Lisa, Stephenson, John, Binns-Hall, Oliver, Knight, Kayleigh. The PodPAD project: a podiatry-led integrated pathway for people with peripheral arterial disease in the UK - a pilot study. Journal of foot and ankle research 2018;11():26. | https://dx.doi.org/10.1186/s13047-018-0269-y |
| [Fattirolli et al. (2015)](https://dx.doi.org/10.1007/s11739-015-1203-y) | Fattirolli, Francesco, Marchionni, Niccolo, Hofer, Stefan, Giannuzzi, Pantaleo, Angelino, Elisabetta, Fioretti, Paolo, Miani, Daniela. The Italian MacNew heart disease health-related quality of life questionnaire: a validation study. Internal and emergency medicine 2015;10(3):359-68. | https://dx.doi.org/10.1007/s11739-015-1203-y |
| [Faulkner et al. (2015)](https://dx.doi.org/10.1177/0269215514555729) | Faulkner, James, McGonigal, Gerard, Woolley, Brandon, Stoner, Lee, Wong, Laikin. A randomized controlled trial to assess the psychosocial effects of early exercise engagement in patients diagnosed with transient ischaemic attack and mild, non-disabling stroke. Clinical rehabilitation 2015;29(8):783-94. | https://dx.doi.org/10.1177/0269215514555729 |
| [Fokkenrood et al. (2015)](https://dx.doi.org/10.1016/j.ejvs.2014.11.002) | Fokkenrood, H J P, Lauret, G J, Verhofstad, N, Bendermacher, B L W, Scheltinga, M R M. The effect of supervised exercise therapy on physical activity and ambulatory activities in patients with intermittent claudication. European journal of vascular and endovascular surgery : the official journal of the European Society for Vascular Surgery 2015;49(2):184-91. | https://dx.doi.org/10.1016/j.ejvs.2014.11.002 |
| [Ford et al. (2018)](https://dx.doi.org/10.1016/j.jacc.2018.09.006) | Ford, Thomas J, Stanley, Bethany, Good, Richard, Rocchiccioli, Paul, McEntegart, Margaret, Watkins, Stuart, et al. Stratified Medical Therapy Using Invasive Coronary Function Testing in Angina: The CorMicA Trial. Journal of the American College of Cardiology 2018;72(23 Pt A):2841-55. | https://dx.doi.org/10.1016/j.jacc.2018.09.006 |
| [Forsberg & (2013)](https://dx.doi.org/10.3138/ptc.2011-54) | Forsberg, Anette. Validity and Reliability of the Swedish Version of the Activities-specific Balance Confidence Scale in People with Chronic Stroke. Physiotherapy Canada. Physiotherapie Canada 2013;65(2):141-47. | https://dx.doi.org/10.3138/ptc.2011-54 |
| [Forster et al. (2015)](https://dx.doi.org/10.1161/STROKEAHA.115.008585) | Forster, Anne, Young, John, Chapman, Katie, Nixon, Jane, Patel, Anita, Holloway, Ivana, et al. Cluster Randomized Controlled Trial: Clinical and Cost-Effectiveness of a System of Longer-Term Stroke Care. Stroke 2015;46(8):2212-19. | https://dx.doi.org/10.1161/STROKEAHA.115.008585 |
| [Fransson et al. (2014)](https://dx.doi.org/10.1111/jocn.12492) | Fransson, Eleonor I, Arenhall, Eva, Steinke, Elaine E, Fridlund, Bengt. Perceptions of intimate relationships in partners before and after a patient's myocardial infarction. Journal of clinical nursing 2014;23(15-16):2196-2004. | https://dx.doi.org/10.1111/jocn.12492 |
| [Fu et al. (2015)](https://dx.doi.org/10.1536/ihj.15-012) | Fu, Tieh-Cheng, Chou, Szu-Ling, Chen, Tai-Tzung, Wang, Chao-Hung, Chang, Hen-Hong. Central and Peripheral Hemodynamic Adaptations During Cardiopulmonary Exercise Test in Heart Failure Patients With Exercise Periodic Breathing. International heart journal 2015;56(4):432-38. | https://dx.doi.org/10.1536/ihj.15-012 |
| [Fu et al. (2016)](https://dx.doi.org/10.1186/s12906-016-1306-7) | Fu, Tieh-Cheng, Lin, Yi-Chung, Chang, Ching-Mao, Chou, Wei-Ling, Yuan, Pei-Hsun, Liu, Min-Hui, et al. Validation of a new simple scale to measure symptoms in heart failure from traditional Chinese medicine view: a cross-sectional questionnaire study. BMC complementary and alternative medicine 2016;16():342. | https://dx.doi.org/10.1186/s12906-016-1306-7 |
| [Gallagher et al. (2019)](https://dx.doi.org/10.1002/ehf2.12363) | Gallagher, Angela M & Lucas, Rebecca. Assessing health-related quality of life in heart failure patients attending an outpatient clinic: a pragmatic approach. ESC heart failure 2019;6(1):3-9. | https://dx.doi.org/10.1002/ehf2.12363 |
| [Gaziano et al. (2016)](https://dx.doi.org/10.1001/jamacardio.2016.1747) | Gaziano, Thomas A, Fonarow, Gregg C, Claggett, Brian, Chan, Wing W, Deschaseaux-Voinet, Celine, Turner, Stuart J, et al. Cost-effectiveness Analysis of Sacubitril/Valsartan vs Enalapril in Patients With Heart Failure and Reduced Ejection Fraction. JAMA cardiology 2016;1(6):666-72. | https://dx.doi.org/10.1001/jamacardio.2016.1747 |
| [Gencer et al. (2016)](https://dx.doi.org/10.1136/openhrt-2016-000419) | Gencer, Baris, Rodondi, Nicolas, Auer, Reto, Nanchen, David, Raber, Lorenz, Klingenberg, Roland, et al. Health utility indexes in patients with acute coronary syndromes. Open heart 2016;3(1):e000419. | https://dx.doi.org/10.1136/openhrt-2016-000419 |
| [Ghanbari-Firoozabadi et al. (2014)](http://ovidsp.ovid.com/ovidweb.cgi?T=JS&PAGE=reference&D=med11&NEWS=N&AN=24653758) | Ghanbari-Firoozabadi, M, Rahimianfar, A A, Reza Vafaii Nasab, M, Namayandeh, S M, Emami, M, Boostani, F, Sherafat, A. A study of the effect of cardiac rehabilitation on heart failure patients' life quality. Journal of medicine and life 2014;7(1):51-54. | http://ovidsp.ovid.com/ovidweb.cgi?T=JS&PAGE=reference&D=med11&NEWS=N&AN=24653758 |
| [Gijsberts et al. (2015)](https://dx.doi.org/10.1136/openhrt-2014-000231) | Gijsberts, Crystel M, Agostoni, Pierfrancesco, Hoefer, Imo E, Asselbergs, Folkert W, Pasterkamp, Gerard, Nathoe, Hendrik, et al. Gender differences in health-related quality of life in patients undergoing coronary angiography. Open heart 2015;2(1):e000231. | https://dx.doi.org/10.1136/openhrt-2014-000231 |
| [Gillard et al. (2015)](https://dx.doi.org/10.1186/s12955-015-0340-3) | Gillard, Patrick J, Sucharew, Heidi, Kleindorfer, Dawn, Belagaje, Samir, Varon, Sepideh, Alwell, Kathleen, et al. The negative impact of spasticity on the health-related quality of life of stroke survivors: a longitudinal cohort study. Health and quality of life outcomes 2015;13():159. | https://dx.doi.org/10.1186/s12955-015-0340-3 |
| [Gingele et al. (2019)](http://dx.doi.org/10.1007/s12471-019-01323-x) | Gingele, A J, Brunner-La Rocca, H P, van Empel, V, van der Weg, K, Gorgels, A, Knackstedt, C, et al. Effects of tailored telemonitoring on functional status and health-related quality of life in patients with heart failure. Netherlands Heart Journal 2019;27(11):565-74. | http://dx.doi.org/10.1007/s12471-019-01323-x |
| [Golicki et al. (2015a)](https://dx.doi.org/10.1007/s11136-014-0834-1) | Golicki, Dominik, Niewada, Maciej, Buczek, Julia, Karlinska, Anna, Kobayashi, Adam, Janssen, M F. Validity of EQ-5D-5L in stroke. Quality of life research : an international journal of quality of life aspects of treatment, care and rehabilitation 2015;24(4):845-50. | https://dx.doi.org/10.1007/s11136-014-0834-1 |
| [Golicki et al. (2015b)](https://dx.doi.org/10.1007/s11136-014-0834-1) | Golicki, Dominik, Niewada, Maciej, Karlinska, Anna, Buczek, Julia, Kobayashi, Adam, Janssen, M F. Comparing responsiveness of the EQ-5D-5L, EQ-5D-3L and EQ VAS in stroke patients. Quality of life research : an international journal of quality of life aspects of treatment, care and rehabilitation 2015;24(6):1555-63. | https://dx.doi.org/10.1007/s11136-014-0873-7 |
| [Gonzalez-Guerrero et al. (2018)](http://dx.doi.org/10.1007/s41999-018-0074-y) | Gonzalez-Guerrero, Jose L, Alonso-Fernandez, Teresa, Hernandez-Mocholi, Miguel A, Gusi, Narcis, Ribera-Casado, Jose M. Cost-effectiveness of a follow-up program for older patients with heart failure: a randomized controlled trial. European Geriatric Medicine 2018;9(4):523-32. | http://dx.doi.org/10.1007/s41999-018-0074-y |
| [Gordon et al. (2013)](https://dx.doi.org/10.1161/STROKEAHA.111.000642) | Gordon, Carron D & Wilks, Rainford. Effect of aerobic exercise (walking) training on functional status and health-related quality of life in chronic stroke survivors: a randomized controlled trial. Stroke 2013;44(4):1179-81. | https://dx.doi.org/10.1161/STROKEAHA.111.000642 |
| [Grady et al. (2014)](https://dx.doi.org/10.1016/j.healun.2013.10.017) | Grady, Kathleen L, Naftel, David, Stevenson, Lynne, Dew, Mary Amanda, Weidner, Gerdi, Pagani, Francis D, et al. Overall quality of life improves to similar levels after mechanical circulatory support regardless of severity of heart failure before implantation. The Journal of heart and lung transplantation : the official publication of the International Society for Heart Transplantation 2014;33(4):412-21. | https://dx.doi.org/10.1016/j.healun.2013.10.017 |
| [Graessel et al. (2014)](https://dx.doi.org/10.1097/MRR.0000000000000060) | Graessel, Elmar & Schmidt, Ralf. Stroke patients after neurological inpatient rehabilitation: a prospective study to determine whether functional status or health-related quality of life predict living at home 2.5 years after discharge. International journal of rehabilitation research. Internationale Zeitschrift fur Rehabilitationsforschung. Revue internationale de recherches de readaptation 2014;37(3):212-19. | https://dx.doi.org/10.1097/MRR.0000000000000060 |
| [Green et al. (2018)](https://dx.doi.org/10.1177/1708538118773618) | Green, Jordan Luke, Harwood, Amy Elizabeth, Smith, George Edward, Das, Tushar, Raza, Ali, Cayton, Thomas, et al. Extracorporeal shockwave therapy for intermittent claudication: Medium-term outcomes from a double-blind randomised placebo-controlled pilot trial. Vascular 2018;26(5):531-39. | https://dx.doi.org/10.1177/1708538118773618 |
| [Groeneveld et al. (2019a)](https://dx.doi.org/10.1016/j.rehab.2018.05.1321) | Groeneveld, I F, Goossens, P H, van Meijeren-Pont, W, Arwert, H J, Meesters, J J L, Rambaran Mishre, A D, et al. Value-Based Stroke Rehabilitation: Feasibility and Results of Patient-Reported Outcome Measures in the First Year After Stroke. Journal of stroke and cerebrovascular diseases : the official journal of National Stroke Association 2019;28(2):499-512. | https://dx.doi.org/10.1016/j.jstrokecerebrovasdis.2018.10.033 |
| [Groeneveld et al. (2019b)](https://dx.doi.org/10.1016/j.rehab.2018.05.1321) | Groeneveld, Iris F, Goossens, Paulien H, van Braak, Inke, van der Pas, Stephanie, Meesters, Jorit J L, Rambaran Mishre, Radha D, et al. Patients' outcome expectations and their fulfilment in multidisciplinary stroke rehabilitation. Annals of physical and rehabilitation medicine 2019;62(1):21-27. | https://dx.doi.org/10.1016/j.rehab.2018.05.1321 |
| [Gu et al. (2020)](https://dx.doi.org/10.1007/s11136-020-02524-3) | Gu, Shuyan, Wang, Xiaoyong, Shi, Lizheng, Sun, Qiuying, Hu, Xiaoqian, Gu, Yuxuan, Sun, Xueshan. Health-related quality of life of type 2 diabetes patients hospitalized for a diabetes-related complication. Quality of life research : an international journal of quality of life aspects of treatment, care and rehabilitation 2020;29(10):2695-7004. | https://dx.doi.org/10.1007/s11136-020-02524-3 |
| [Guder et al. (2015)](https://dx.doi.org/10.1002/ejhf.252) | Guder, Gulmisal, Stork, Stefan, Gelbrich, Goetz, Brenner, Susanne, Deubner, Nikolas, Morbach, Caroline, et al. Nurse-coordinated collaborative disease management improves the quality of guideline-recommended heart failure therapy, patient-reported outcomes, and left ventricular remodelling. European journal of heart failure 2015;17(4):442-52. | https://dx.doi.org/10.1002/ejhf.252 |
| [Guidon & (2013)](https://dx.doi.org/10.3109/09638288.2012.694963) | Guidon, Marie. One-year effect of a supervised exercise programme on functional capacity and quality of life in peripheral arterial disease. Disability and rehabilitation 2013;35(5):397-404. | https://dx.doi.org/10.3109/09638288.2012.694963 |
| [Guo et al. (2017)](http://dx.doi.org/10.1080/02687038.2016.1261269) | Guo, Yiting Emily, Togher, Leanne, Power, Emma, Heard, Rob, Luo, Nan, Koh, Gerald C H, Yap Emma. Sensitivity to change and responsiveness of the Stroke and Aphasia Quality-of-Life Scale (SAQOL) in a Singapore stroke population. Aphasiology 2017;31(4):427-46. | http://dx.doi.org/10.1080/02687038.2016.1261269 |
| [Hamo et al. (2015)](https://dx.doi.org/10.1161/CIRCHEARTFAILURE.114.001838) | Hamo, Carine E, Heitner, John F, Pfeffer, Marc A, Kim, Hae-Young, Kenwood, Christopher T, Assmann, Susan F, et al. Baseline distribution of participants with depression and impaired quality of life in the Treatment of Preserved Cardiac Function Heart Failure with an Aldosterone Antagonist Trial. Circulation. Heart failure 2015;8(2):268-77. | https://dx.doi.org/10.1161/CIRCHEARTFAILURE.114.001838 |
| [Han et al. (2019)](https://dx.doi.org/10.1136/bmjopen-2018-028673) | Han, Junhee, Lee, Hae In, Shin, Yong-Il, Son, Ju Hyun, Kim, Soo-Yeon, Kim, Deog Young, et al. Factors influencing return to work after stroke: the Korean Stroke Cohort for Functioning and Rehabilitation (KOSCO) Study. BMJ open 2019;9(7):e028673. | https://dx.doi.org/10.1136/bmjopen-2018-028673 |
| [Hansson et al. (2013)](http://dx.doi.org/10.1111/j.1471-6712.2012.01041.x) | Hansson, Eva Ekvall, Beckman, Anders, Wihlborg, Anna, Persson, Sylvia, Troein, Margareta, Wihlborg, Anna. Satisfaction with rehabilitation in relation to self-perceived quality of life and function among patients with stroke - a 12 month follow-up. Scandinavian Journal of Caring Sciences 2013;27(2):373-79. | http://dx.doi.org/10.1111/j.1471-6712.2012.01041.x |
| [Hansson et al. (2014)](http://dx.doi.org/10.1177/1474515114567035) | Hansson, Elisabeth, Ekman, Inger, Wolf, Axel, Dudas, Kerstin, Olsson, Lars-Eric, Swedberg, Karl, et al. Person-centred care for patients with chronic heart failure - A cost-utility analysis. European Journal of Cardiovascular Nursing 2014;15(4):276-84. | http://dx.doi.org/10.1177/1474515114567035 |
| [Harno et al. (2014)](https://dx.doi.org/10.1212/WNL.0000000000000818) | Harno, Hanna, Haapaniemi, Elena, Putaala, Jukka, Haanpaa, Maija, Makela, Jyrki P, Kalso, Eija. Central poststroke pain in young ischemic stroke survivors in the Helsinki Young Stroke Registry. Neurology 2014;83(13):1147-54. | https://dx.doi.org/10.1212/WNL.0000000000000818 |
| [Hayes et al. (2016)](https://dx.doi.org/10.1016/j.jval.2015.10.010) | Hayes, Alison, Arima, Hisatomi, Woodward, Mark, Chalmers, John, Poulter, Neil, Hamet, Pavel. Changes in Quality of Life Associated with Complications of Diabetes: Results from the ADVANCE Study. Value in health : the journal of the International Society for Pharmacoeconomics and Outcomes Research 2016;19(1):36-41. | https://dx.doi.org/10.1016/j.jval.2015.10.010 |
| [Hays et al. (2014)](https://dx.doi.org/10.1007/s11136-013-0503-9) | Hays, Ron D, Reeve, Bryce B, Smith, Ashley Wilder. Associations of cancer and other chronic medical conditions with SF-6D preference-based scores in Medicare beneficiaries. Quality of life research : an international journal of quality of life aspects of treatment, care and rehabilitation 2014;23(2):385-91. | https://dx.doi.org/10.1007/s11136-013-0503-9 |
| [Henriksson et al. (2014)](https://dx.doi.org/10.1136/openhrt-2014-000051) | Henriksson, Catrin, Larsson, Margareta, Herlitz, Johan, Karlsson, Jan-Erik, Wernroth, Lisa. Influence of health-related quality of life on time from symptom onset to hospital arrival and the risk of readmission in patients with myocardial infarction. Open heart 2014;1(1):e000051. | https://dx.doi.org/10.1136/openhrt-2014-000051 |
| [Herman et al. (2018)](https://dx.doi.org/10.1016/j.jdiacomp.2018.05.007) | Herman, William H, Braffett, Barbara H, Kuo, Shihchen, Lee, Joyce M, Brandle, Michael, Jacobson, Alan M, Prosser, Lisa A. What are the clinical, quality-of-life, and cost consequences of 30years of excellent vs. poor glycemic control in type 1 diabetes?. Journal of diabetes and its complications 2018;32(10):911-15. | https://dx.doi.org/10.1016/j.jdiacomp.2018.05.007 |
| [Hickey et al. (2013)](https://dx.doi.org/10.1111/j.1365-2702.2012.04307.x) | Hickey, Kathleen T, Reiffel, James, Sciacca, Robert R, Whang, William, Biviano, Angelo, Baumeister, Maurita, et al. Correlating perceived arrhythmia symptoms and quality of life in an older population with heart failure: a prospective, single centre, urban clinic study. Journal of clinical nursing 2013;22(3-4):434-44. | https://dx.doi.org/10.1111/j.1365-2702.2012.04307.x |
| [Hokstad et al. (2016)](https://dx.doi.org/10.2340/16501977-2051) | Hokstad, Anne, Indredavik, Bent, Bernhardt, Julie, Langhammer, Birgitta, Gunnes, Mari, Lundemo, Christine, Bovim, Martina Reiten. Upright activity within the first week after stroke is associated with better functional outcome and health-related quality of life: A Norwegian multi-site study. Journal of rehabilitation medicine 2016;48(3):280-86. | https://dx.doi.org/10.2340/16501977-2051 |
| [Hong et al. (2018)](https://dx.doi.org/10.1007/s40261-018-0659-8) | Hong, Sung-Hyun, Lee, Jae-Yeon, Park, Sun-Kyeong, Nam, Jin Hyun, Song, Hyun Jin, Park, Sun-Young. The Utility of 5 Hypothetical Health States in Heart Failure Using Time Trade-Off (TTO) and EQ-5D-5L in Korea. Clinical drug investigation 2018;38(8):727-36. | https://dx.doi.org/10.1007/s40261-018-0659-8 |
| [Hornslien et al. (2013)](https://dx.doi.org/10.1161/STROKEAHA.113.001022) | Hornslien, Astrid G, Sandset, Else C, Bath, Philip M, Wyller, Torgeir B, Berge, Eivind. Effects of candesartan in acute stroke on cognitive function and quality of life: results from the Scandinavian Candesartan Acute Stroke Trial. Stroke 2013;44(7):2022-24. | https://dx.doi.org/10.1161/STROKEAHA.113.001022 |
| [Hotter et al. (2018)](https://dx.doi.org/10.1177/2396987318771174) | Hotter, Benjamin, Padberg, Inken, Liebenau, Andrea, Knispel, Petra, Heel, Sabine, Steube, Diethard, et al. Identifying unmet needs in long-term stroke care using in-depth assessment and the Post-Stroke Checklist - The Managing Aftercare for Stroke (MAS-I) study. European stroke journal 2018;3(3):237-45. | https://dx.doi.org/10.1177/2396987318771174 |
| [Hsieh et al. (2018)](https://dx.doi.org/10.1016/j.apmr.2018.03.017) | Hsieh, Yu-Wei, Chang, Ku-Chou, Hung, Jen-Wen, Wu, Ching-Yi, Fu, Mu-Hui. Effects of Home-Based Versus Clinic-Based Rehabilitation Combining Mirror Therapy and Task-Specific Training for Patients With Stroke: A Randomized Crossover Trial. Archives of physical medicine and rehabilitation 2018;99(12):2399-4007. | https://dx.doi.org/10.1016/j.apmr.2018.03.017 |
| [Hsu et al. (2019)](https://dx.doi.org/10.4103/tcmj.tcmj_95_18) | Hsu, You-Chien, Chen, Guei-Chiuan, Chen, Pei-Ya. Postacute care model of stroke in one hospital. Ci ji yi xue za zhi = Tzu-chi medical journal 2019;31(4):260-65. | https://dx.doi.org/10.4103/tcmj.tcmj_95_18 |
| [Huber et al. (2016)](http://ovidsp.ovid.com/ovidweb.cgi?T=JS&PAGE=reference&D=med13&NEWS=N&AN=27318487) | Huber, Alexandra & Oldridge, Neil. International SF-36 reference values in patients with ischemic heart disease. Quality of life research : an international journal of quality of life aspects of treatment, care and rehabilitation 2016;25(11):2787-98. | http://ovidsp.ovid.com/ovidweb.cgi?T=JS&PAGE=reference&D=med13&NEWS=N&AN=27318487 |
| [Hurdus et al. (2020)](https://dx.doi.org/10.1136/heartjnl-2020-316920) | Hurdus, Ben, Munyombwe, Theresa, Dondo, Tatendashe Bernadette, Aktaa, Suleman, Oliver, Gerrard, Hall, Marlous, et al. Association of cardiac rehabilitation and health-related quality of life following acute myocardial infarction. Heart (British Cardiac Society) 2020;106(22):1726-31. | https://dx.doi.org/10.1136/heartjnl-2020-316920 |
| [Hutchinson et al. (2015)](https://dx.doi.org/10.1186/s12955-015-0260-2) | Hutchinson, Anastasia F, Graco, Marnie, Rasekaba, Tshepo Mokuedi, Parikh, Sumit, Berlowitz, David John. Relationship between health-related quality of life, comorbidities and acute health care utilisation, in adults with chronic conditions. Health and quality of life outcomes 2015;13():69. | https://dx.doi.org/10.1186/s12955-015-0260-2 |
| [Hwang et al. (2018)](http://dx.doi.org/10.1016/j.hlc.2018.11.010) | Hwang, Rita, Mandrusiak, Allison, Russell, Trevor, Morris, Norman R, Bruning, Jared, Korczyk, Dariusz, Peters Rita. Cost-Utility Analysis of Home-based Telerehabilitation Compared with Centre-based Rehabilitation in Patients with Heart Failure. Heart Lung and Circulation 2018;():n. pag.. | http://dx.doi.org/10.1016/j.hlc.2018.11.010 |
| [Im et al. (2020)](https://dx.doi.org/10.1016/j.jstrokecerebrovasdis.2020.105406) | Im, Hyo Won, Kim, Won-Seok, Kim, SeungYeun. Prevalence of Worsening Problems Using Post-Stroke Checklist and Associations with Quality of Life in Patients with Stroke. Journal of stroke and cerebrovascular diseases : the official journal of National Stroke Association 2020;29(12):105406. | https://dx.doi.org/10.1016/j.jstrokecerebrovasdis.2020.105406 |
| [Jackson et al. (2018)](https://dx.doi.org/10.2147/DDDT.S148949) | Jackson, James Ds, Cotton, Sarah E, Bruce Wirta, Sara, Proenca, Catia C, Zhang, Milun, Lahoz, Raquel. Burden of heart failure on patients from China: results from a cross-sectional survey. Drug design, development and therapy 2018;12():1659-68. | https://dx.doi.org/10.2147/DDDT.S148949 |
| [Janssen et al. (2013)](https://dx.doi.org/10.1007/s11136-012-0322-4) | Janssen, M F, Pickard, A Simon, Golicki, Dominik, Gudex, Claire, Niewada, Maciej, Scalone, Luciana, Swinburn, Paul. Measurement properties of the EQ-5D-5L compared to the EQ-5D-3L across eight patient groups: a multi-country study. Quality of life research : an international journal of quality of life aspects of treatment, care and rehabilitation 2013;22(7):1717-27. | https://dx.doi.org/10.1007/s11136-012-0322-4 |
| [Jelani et al. (2019)](http://dx.doi.org/10.1177/1358863X19872542) | Jelani, Qurat-Ul-Ain, Jhamnani, Sunny, Spatz, Erica S, Desai, Nihar R, Shah, Samit, Attaran, Robert, et al. Financial barriers in accessing medical care for peripheral artery disease are associated with delay of presentation and adverse health status outcomes in the United States. Vascular Medicine (United Kingdom) 2019;():n. pag.. | http://dx.doi.org/10.1177/1358863X19872542 |
| [Jelani et al. (2020)](https://dx.doi.org/10.1161/JAHA.119.014583) | Jelani, Qurat-Ul-Ain, Mena-Hurtado, Carlos, Burg, Matthew, Soufer, Robert, Gosch, Kensey, Jones, Philip G, et al. Relationship Between Depressive Symptoms and Health Status in Peripheral Artery Disease: Role of Sex Differences. Journal of the American Heart Association 2020;9(16):e014583. | https://dx.doi.org/10.1161/JAHA.119.014583 |
| [Jenkinson et al. (2013)](https://dx.doi.org/10.1161/STROKEAHA.113.001847) | Jenkinson, Crispin, Fitzpatrick, Ray, Crocker, Helen. The Stroke Impact Scale: validation in a UK setting and development of a SIS short form and SIS index. Stroke 2013;44(9):2532-35. | https://dx.doi.org/10.1161/STROKEAHA.113.001847 |
| [Jeon et al. (2017a)](https://dx.doi.org/10.5535/arm.2017.41.4.556) | Jeon, Hyunkyu, Sohn, Min Kyun, Jeon, Minsoo. Clinical Characteristics of Sleep-Disordered Breathing in Subacute Phase of Stroke. Annals of rehabilitation medicine 2017;41(4):556-63. | https://dx.doi.org/10.5535/arm.2017.41.4.556 |
| [Jeon et al. (2017b)](https://dx.doi.org/10.5535/arm.2017.41.4.556) | Jeon, Na Eun, Kwon, Kyoung Min, Kim, Yeo Hyung. The Factors Associated With Health-Related Quality of Life in Stroke Survivors Age 40 and Older. Annals of rehabilitation medicine 2017;41(5):743-52. | https://dx.doi.org/10.5535/arm.2017.41.5.743 |
| [Jia & (2016)](https://dx.doi.org/10.1007/s11136-016-1226-5) | Jia, Haomiao. Impact of nine chronic conditions for US adults aged 65 years and older: an application of a hybrid estimator of quality-adjusted life years throughout remainder of lifetime. Quality of life research : an international journal of quality of life aspects of treatment, care and rehabilitation 2016;25(8):1921-29. | https://dx.doi.org/10.1007/s11136-016-1226-5 |
| [Jia et al. (2018)](https://dx.doi.org/10.1097/MLR.0000000000000943) | Jia, Haomiao, Lubetkin, Erica I, Barile, John P, Horner-Johnson, Willi, DeMichele, Kimberly, Stark, Debra S, Zack, Matthew M. Quality-adjusted Life Years (QALY) for 15 Chronic Conditions and Combinations of Conditions Among US Adults Aged 65 and Older. Medical care 2018;56(8):740-46. | https://dx.doi.org/10.1097/MLR.0000000000000943 |
| [Jiao et al. (2017)](http://dx.doi.org/10.1186/s12955-017-0699-4) | Jiao, Fangfang, Wong, Carlos King Ho, Lam, Cindy Lo Kuen, Gangwani, Rita, Tan, Kathryn Choon Beng. Health-related quality of life and health preference of Chinese patients with diabetes mellitus managed in primary care and secondary care setting: Decrements associated with individual complication and number of complications. Health and Quality of Life Outcomes 2017;15(1):125. | http://dx.doi.org/10.1186/s12955-017-0699-4 |
| [Johnston et al. (2016)](https://dx.doi.org/10.1016/j.ahj.2016.05.005) | Johnston, Nina, Bodegard, Johan, Jerstrom, Susanna, Akesson, Johanna, Brorsson, Hilja, Alfredsson, Joakim, et al. Effects of interactive patient smartphone support app on drug adherence and lifestyle changes in myocardial infarction patients: A randomized study. American heart journal 2016;178():85-94. | https://dx.doi.org/10.1016/j.ahj.2016.05.005 |
| [Jones et al. (2016)](http://dx.doi.org/10.1136/bmjopen-2015-008900) | Jones, Fiona, Grant, Robert, Gage, Heather, Drummond, Avril, Bhalla, Ajay, Lennon, Sheila, et al. Feasibility study of an integrated stroke self-management programme: A cluster-randomised controlled trial. BMJ Open 2016;6(1):e008900. | http://dx.doi.org/10.1136/bmjopen-2015-008900 |
| [Jonsson et al. (2014)](https://dx.doi.org/10.1161/STROKEAHA.114.005164) | Jonsson, Ann-Cathrin, Delavaran, Hossein, Iwarsson, Susanne, Stahl, Agneta, Norrving, Bo. Functional status and patient-reported outcome 10 years after stroke: the Lund Stroke Register. Stroke 2014;45(6):1784-90. | https://dx.doi.org/10.1161/STROKEAHA.114.005164 |
| [Jorge et al. (2017)](https://dx.doi.org/10.5935/abc.20170123) | Jorge, Antonio Jose Lagoeiro, Rosa, Maria Luiza Garcia, Correia, Dayse Mary da Silva, Martins, Wolney de Andrade, Ceron, Diana Maria Martinez, Coelho, Leonardo Chaves Ferreira, et al. Evaluation of Quality of Life in Patients with and without Heart Failure in Primary Care. Arquivos brasileiros de cardiologia 2017;109(3):248-52. | https://dx.doi.org/10.5935/abc.20170123 |
| [Joundi et al. (2021)](https://dx.doi.org/10.1161/STROKEAHA.120.033872) | Joundi, Raed A, Rebchuk, Alexander D, Field, Thalia S, Smith, Eric E, Goyal, Mayank, Demchuk, Andrew M, et al. Health-Related Quality of Life Among Patients With Acute Ischemic Stroke and Large Vessel Occlusion in the ESCAPE Trial. Stroke 2021;():STROKEAHA120033872. | https://dx.doi.org/10.1161/STROKEAHA.120.033872 |
| [Jovanic et al. (2018)](https://dx.doi.org/10.3390/ijerph15081761) | Jovanic, Marija, Zdravkovic, Marija, Stanisavljevic, Dejana. Exploring the Importance of Health Literacy for the Quality of Life in Patients with Heart Failure. International journal of environmental research and public health 2018;15(8):n. pag.. | https://dx.doi.org/10.3390/ijerph15081761 |
| [Kang et al. (2018)](https://dx.doi.org/10.1177/1099800418772346) | Kang, Youjeong, Steele, Bonnie G, Burr, Robert L. Mortality in Advanced Chronic Obstructive Pulmonary Disease and Heart Failure Following Cardiopulmonary Rehabilitation. Biological research for nursing 2018;20(4):429-39. | https://dx.doi.org/10.1177/1099800418772346 |
| [Kang et al. (2019)](https://dx.doi.org/10.1186/s12911-019-1000-z) | Kang, Yi-No, Shen, Hsiu-Nien, Lin, Chia-Yun, Elwyn, Glyn, Huang, Szu-Chi, Wu, Tsung-Fu. Does a Mobile app improve patients' knowledge of stroke risk factors and health-related quality of life in patients with stroke? A randomized controlled trial. BMC medical informatics and decision making 2019;19(1):282. | https://dx.doi.org/10.1186/s12911-019-1000-z |
| [Karapolat et al. (2013)](https://dx.doi.org/10.1016/j.transproceed.2013.06.009) | Karapolat, H, Engin, C, Eroglu, M, Yagdi, T, Zoghi, M, Nalbantgil, S, et al. Efficacy of the cardiac rehabilitation program in patients with end-stage heart failure, heart transplant patients, and left ventricular assist device recipients. Transplantation proceedings 2013;45(9):3381-85. | https://dx.doi.org/10.1016/j.transproceed.2013.06.009 |
| [Karlstrom et al. (2016)](https://dx.doi.org/10.1186/s12872-016-0221-7) | Karlstrom, Patric, Johansson, Peter, Dahlstrom, Ulf, Boman, Kurt. Can BNP-guided therapy improve health-related quality of life, and do responders to BNP-guided heart failure treatment have improved health-related quality of life? Results from the UPSTEP study. BMC cardiovascular disorders 2016;16():39. | https://dx.doi.org/10.1186/s12872-016-0221-7 |
| [Katzan et al. (2017)](https://dx.doi.org/10.1161/JAHA.116.005356) | Katzan, Irene L, Thompson, Nicolas R, Lapin, Brittany. Added Value of Patient-Reported Outcome Measures in Stroke Clinical Practice. Journal of the American Heart Association 2017;6(7):n. pag.. | https://dx.doi.org/10.1161/JAHA.116.005356 |
| [Ketilsdottir et al. (2019)](https://dx.doi.org/10.1002/ehf2.12369) | Ketilsdottir, Audur & Ingadottir, Brynja. Self-reported health and quality of life outcomes of heart failure patients in the aftermath of a national economic crisis: a cross-sectional study. ESC heart failure 2019;6(1):111-21. | https://dx.doi.org/10.1002/ehf2.12369 |
| [Khattab et al. (2013)](https://dx.doi.org/10.1177/2047487312447751) | Khattab, Ahmed A, Knecht, Matthias, Meier, Bernhard, Windecker, Stephan, Schmid, Jean-Paul, Wilhelm, Matthias. Persistence of uncontrolled cardiovascular risk factors in patients treated with percutaneous interventions for stable coronary artery disease not receiving cardiac rehabilitation. European journal of preventive cardiology 2013;20(5):743-49. | https://dx.doi.org/10.1177/2047487312447751 |
| [Kiadaliri et al. (2014)](https://dx.doi.org/10.3390/ijerph110504939) | Kiadaliri, Aliasghar A, Gerdtham, Ulf-G, Eliasson, Bjorn, Gudbjornsdottir, Soffia, Svensson, Ann-Marie. Health utilities of type 2 diabetes-related complications: a cross-sectional study in Sweden. International journal of environmental research and public health 2014;11(5):4939-52. | https://dx.doi.org/10.3390/ijerph110504939 |
| [Kielbergerova et al. (2015)](https://dx.doi.org/10.1007/s12975-015-0418-6) | Kielbergerova, Lenka, Mayer, Otto Jr, Vanek, Jiri, Bruthans, Jan, Wohlfahrt, Peter. Quality of life predictors in chronic stable post-stroke patients and prognostic value of SF-36 score as a mortality surrogate. Translational stroke research 2015;6(5):375-83. | https://dx.doi.org/10.1007/s12975-015-0418-6 |
| [Kim & (2019)](https://dx.doi.org/10.1111/ggi.13797) | Kim, Yoonjung. Association between quality of life and sleep time among community-dwelling stroke survivors: Findings from a nationally representative survey. Geriatrics & gerontology international 2019;19(12):1226-30. | https://dx.doi.org/10.1111/ggi.13797 |
| [Kim et al. (2015)](https://dx.doi.org/10.1097/MRR.0000000000000103) | Kim, HoYoung & Kim, You Lim. Effects of therapeutic Tai Chi on balance, gait, and quality of life in chronic stroke patients. International journal of rehabilitation research. Internationale Zeitschrift fur Rehabilitationsforschung. Revue internationale de recherches de readaptation 2015;38(2):156-61. | https://dx.doi.org/10.1097/MRR.0000000000000103 |
| [Kim et al. (2016)](https://dx.doi.org/10.5535/arm.2016.40.6.1010) | Kim, Gowun, Min, David, Lee, Eun-Ok. Impact of Co-occurring Dysarthria and Aphasia on Functional Recovery in Post-stroke Patients. Annals of rehabilitation medicine 2016;40(6):1010-17. | https://dx.doi.org/10.5535/arm.2016.40.6.1010 |
| [Kim et al. (2020)](https://dx.doi.org/10.2196/15377) | Kim, Do Yeon, Kwon, Hee, Nam, Ki-Woong, Lee, Yongseok, Kwon, Hyung-Min. Remote Management of Poststroke Patients With a Smartphone-Based Management System Integrated in Clinical Care: Prospective, Nonrandomized, Interventional Study. Journal of medical Internet research 2020;22(2):e15377. | https://dx.doi.org/10.2196/15377 |
| [Kirk et al. (2014)](https://dx.doi.org/10.1177/0269215513502211) | Kirk, Hayden, Kersten, Paula, Crawford, Pamela, Keens, Angela, Ashburn, Ann. The cardiac model of rehabilitation for reducing cardiovascular risk factors post transient ischaemic attack and stroke: a randomized controlled trial. Clinical rehabilitation 2014;28(4):339-49. | https://dx.doi.org/10.1177/0269215513502211 |
| [Klassen et al. (2020)](https://dx.doi.org/10.1161/STROKEAHA.120.029245) | Klassen, Tara D, Dukelow, Sean P, Bayley, Mark T, Benavente, Oscar, Hill, Michael D, Krassioukov, Andrei, et al. Higher Doses Improve Walking Recovery During Stroke Inpatient Rehabilitation. Stroke 2020;51(9):2639-48. | https://dx.doi.org/10.1161/STROKEAHA.120.029245 |
| [Kocic et al. (2020)](http://dx.doi.org/10.3390/medicina56120666) | Kocic, Ilija D, Rancic, Natasa K, Kocic, Biljana N, Otasevic, Suzana A, Mandic, Milan N, Veljkovic Dejan R. Health-Related Quality of Life in Stroke Survivors in Relation to the Type of Inpatient Rehabilitation in Serbia: A Prospective Cohort Study. Medicina (Kaunas, Lithuania) 2020;56(12):n. pag.. | http://dx.doi.org/10.3390/medicina56120666 |
| [Kraai et al. (2013)](https://dx.doi.org/10.1093/eurjhf/hft071) | Kraai, Imke H, Vermeulen, Karin M, Luttik, Marie Louise A, Hoekstra, Tialda, Jaarsma, Tiny. Preferences of heart failure patients in daily clinical practice: quality of life or longevity?. European journal of heart failure 2013;15(10):1113-21. | https://dx.doi.org/10.1093/eurjhf/hft071 |
| [Krack et al. (2018)](https://dx.doi.org/10.1186/s12877-018-0827-y) | Krack, Gundula, Holle, Rolf, Kirchberger, Inge, Kuch, Bernhard, Amann, Ute. Determinants of adherence and effects on health-related quality of life after myocardial infarction: a prospective cohort study. BMC geriatrics 2018;18(1):136. | https://dx.doi.org/10.1186/s12877-018-0827-y |
| [Krishnan et al. (2020)](https://dx.doi.org/10.1016/j.ekir.2020.09.028) | Krishnan, Anoushka, Teixeira-Pinto, Armando, Lim, Wai H, Howard, Kirsten, Chapman, Jeremy R, Castells, Antoni, et al. Health-Related Quality of Life in People Across the Spectrum of CKD. Kidney international reports 2020;5(12):2264-74. | https://dx.doi.org/10.1016/j.ekir.2020.09.028 |
| [Kularatna et al. (2020)](https://dx.doi.org/10.1186/s12955-020-01368-2) | Kularatna, Sanjeewa, Senanayake, Sameera, Chen, Gang. Mapping the Minnesota living with heart failure questionnaire (MLHFQ) to EQ-5D-5L in patients with heart failure. Health and quality of life outcomes 2020;18(1):115. | https://dx.doi.org/10.1186/s12955-020-01368-2 |
| [Kuo et al. (2019)](https://dx.doi.org/10.1080/13607863.2017.1414148) | Kuo, Li-Min, Tsai, Wen-Che, Chiu, Ming-Jang, Tang, Li-Yu, Lee, Huey-Jane. Cognitive dysfunction predicts worse health-related quality of life for older stroke survivors: a nationwide population-based survey in Taiwan. Aging & mental health 2019;23(3):305-10. | https://dx.doi.org/10.1080/13607863.2017.1414148 |
| [Kuo et al. (2021)](https://dx.doi.org/10.1111/jdi.13520) | Kuo, Shihchen, Yang, Chun-Ting, Chen, Hsuan-Ying. Valuing health states of people with type 2 diabetes: Analyses of the nationwide representative linked databases. Journal of diabetes investigation 2021;():n. pag.. | https://dx.doi.org/10.1111/jdi.13520 |
| [Kweon et al. (2017)](https://dx.doi.org/10.5535/arm.2017.41.2.248) | Kweon, Sehi, Sohn, Min Kyun, Jeong, Jin Ok, Kim, Soojae, Jeon, Hyunkyu, Lee, Hyewon, et al. Quality of Life and Awareness of Cardiac Rehabilitation Program in People With Cardiovascular Diseases. Annals of rehabilitation medicine 2017;41(2):248-56. | https://dx.doi.org/10.5535/arm.2017.41.2.248 |
| [Kwon et al. (2018)](https://dx.doi.org/10.1371/journal.pone.0195713) | Kwon, SuYeon, Park, Ji-Hong, Kim, Won-Seok, Han, Kyungdo, Lee, Yookyung. Health-related quality of life and related factors in stroke survivors: Data from Korea National Health and Nutrition Examination Survey (KNHANES) 2008 to 2014. PloS one 2018;13(4):e0195713. | https://dx.doi.org/10.1371/journal.pone.0195713 |
| [Labberton et al. (2020)](https://dx.doi.org/10.1007/s11136-020-02516-3) | Labberton, Angela S, Augestad, Liv Ariane, Thommessen, Bente. The association of stroke severity with health-related quality of life in survivors of acute cerebrovascular disease and their informal caregivers during the first year post stroke: a survey study. Quality of life research : an international journal of quality of life aspects of treatment, care and rehabilitation 2020;29(10):2679-93. | https://dx.doi.org/10.1007/s11136-020-02516-3 |
| [Lai et al. (2017)](https://dx.doi.org/10.2147/PPA.S136041) | Lai, Chung-Liang, Tsai, Ming-Miau, Luo, Jia-Yuan, Liao, Wan-Chun, Hsu, Pi-Shan. Post-acute care for stroke - a retrospective cohort study in Taiwan. Patient preference and adherence 2017;11():1309-15. | https://dx.doi.org/10.2147/PPA.S136041 |
| [Lamberti et al. (2017)](https://dx.doi.org/10.23736/S1973-9087.16.04322-7) | Lamberti, Nicola, Straudi, Sofia, Malagoni, Anna Maria, Argiro, Matteo, Felisatti, Michele, Nardini, Eleonora, et al. Effects of low-intensity endurance and resistance training on mobility in chronic stroke survivors: a pilot randomized controlled study. European journal of physical and rehabilitation medicine 2017;53(2):228-39. | https://dx.doi.org/10.23736/S1973-9087.16.04322-7 |
| [Lans et al. (2018)](https://dx.doi.org/10.1002/ehf2.12230) | Lans, Charlotta, Cider, Asa, Nylander, Eva. Peripheral muscle training with resistance exercise bands in patients with chronic heart failure. Long-term effects on walking distance and quality of life; a pilot study. ESC heart failure 2018;5(2):241-48. | https://dx.doi.org/10.1002/ehf2.12230 |
| [Larsen et al. (2017)](https://dx.doi.org/10.1186/s12955-017-0760-3) | Larsen, Anne Sofie F, Reiersen, Anne Therese, Jacobsen, Morten B, Klow, Nils-Einar, Nordanstig, Joakim, Morgan, Mark. Validation of the Vascular quality of life questionnaire - 6 for clinical use in patients with lower limb peripheral arterial disease. Health and quality of life outcomes 2017;15(1):184. | https://dx.doi.org/10.1186/s12955-017-0760-3 |
| [Lawrie et al. (2018)](https://dx.doi.org/10.1186/s40814-018-0345-x) | Lawrie, Sophie, Dong, Yun, Steins, Dax, Xia, Zhidao, Esser, Patrick, Sun, Shanbin, et al. Evaluation of a smartwatch-based intervention providing feedback of daily activity within a research-naive stroke ward: a pilot randomised controlled trial. Pilot and feasibility studies 2018;4():157. | https://dx.doi.org/10.1186/s40814-018-0345-x |
| [Lawson et al. (2018)](https://dx.doi.org/10.1371/journal.pmed.1002540) | Lawson, Claire A, Solis-Trapala, Ivonne, Dahlstrom, Ulf, Mamas, Mamas, Jaarsma, Tiny, Kadam, Umesh T. Comorbidity health pathways in heart failure patients: A sequences-of-regressions analysis using cross-sectional data from 10,575 patients in the Swedish Heart Failure Registry. PLoS medicine 2018;15(3):e1002540. | https://dx.doi.org/10.1371/journal.pmed.1002540 |
| [Laxy et al. (2015)](https://dx.doi.org/10.1016/j.jval.2015.07.003) | Laxy, Michael, Hunger, Matthias, Stark, Renee, Meisinger, Christa, Kirchberger, Inge, Heier, Margit, von Scheidt, Wolfgang. The Burden of Diabetes Mellitus in Patients with Coronary Heart Disease: A Methodological Approach to Assess Quality-Adjusted Life-Years Based on Individual-Level Longitudinal Survey Data. Value in health : the journal of the International Society for Pharmacoeconomics and Outcomes Research 2015;18(8):969-76. | https://dx.doi.org/10.1016/j.jval.2015.07.003 |
| [Laxy et al. (2021)](https://dx.doi.org/10.1016/j.jval.2020.09.017) | Laxy, Michael, Becker, Jana, Kahm, Katharina, Holle, Rolf, Peters, Annette, Thorand, Barbara, Schwettmann, Lars. Utility Decrements Associated With Diabetes and Related Complications: Estimates From a Population-Based Study in Germany. Value in health : the journal of the International Society for Pharmacoeconomics and Outcomes Research 2021;24(2):274-80. | https://dx.doi.org/10.1016/j.jval.2020.09.017 |
| [Lee et al. (2018)](https://dx.doi.org/10.1097/PHM.0000000000000920) | Lee, So Young, Im, Sang Hee, Kim, Bo Ryun. The Effects of a Motorized Aquatic Treadmill Exercise Program on Muscle Strength, Cardiorespiratory Fitness, and Clinical Function in Subacute Stroke Patients: A Randomized Controlled Pilot Trial. American journal of physical medicine & rehabilitation 2018;97(8):533-40. | https://dx.doi.org/10.1097/PHM.0000000000000920 |
| [Lee et al. (2019)](https://dx.doi.org/10.1093/ehjci/jey099) | Lee, Seung-Pyo, Seo, Jae-Kyung, Hwang, In-Chang, Park, Jun-Bean, Park, Eun-Ah, Lee, Whal, et al. Coronary computed tomography angiography vs. myocardial single photon emission computed tomography in patients with intermediate risk chest pain: a randomized clinical trial for cost-effectiveness comparison based on real-world cost. European heart journal cardiovascular Imaging 2019;20(4):417-25. | https://dx.doi.org/10.1093/ehjci/jey099 |
| [Leow et al. (2013)](https://dx.doi.org/10.1371/journal.pone.0067138) | Leow, Melvin Khee-Shing, Griva, Konstadina, Choo, Robin, Wee, Hwee-Lin, Thumboo, Julian, Tai, E Shyong. Determinants of Health-Related Quality of Life (HRQoL) in the Multiethnic Singapore Population - A National Cohort Study. PloS one 2013;8(6):e67138. | https://dx.doi.org/10.1371/journal.pone.0067138 |
| [Lerdal et al. (2019)](https://dx.doi.org/10.1186/s41687-019-0142-1) | Lerdal, Anners, Hofoss, Dag, Gay, Caryl L. Perception of illness among patients with heart failure is related to their general health independently of their mood and functional capacity. Journal of patient-reported outcomes 2019;3(1):55. | https://dx.doi.org/10.1186/s41687-019-0142-1 |
| [Levytska et al. (2016)](http://ovidsp.ovid.com/ovidweb.cgi?T=JS&PAGE=reference&D=emed17&NEWS=N&AN=616515376) | Levytska, O R, Hromovyk, B P, Levytska, O E. Sociological measuring of quality of life of aged people who have had acute cerebrovascular accidents. Pharmacia 2016;63(4):15-20. | http://ovidsp.ovid.com/ovidweb.cgi?T=JS&PAGE=reference&D=emed17&NEWS=N&AN=616515376 |
| [Lewis et al. (2014)](https://dx.doi.org/10.1016/j.jchf.2013.12.003) | Lewis, Eldrin F, Li, Yanhong, Pfeffer, Marc A, Solomon, Scott D, Weinfurt, Kevin P, Velazquez, Eric J, et al. Impact of cardiovascular events on change in quality of life and utilities in patients after myocardial infarction: a VALIANT study (valsartan in acute myocardial infarction). JACC. Heart failure 2014;2(2):159-65. | https://dx.doi.org/10.1016/j.jchf.2013.12.003 |
| [Li et al. (2013)](https://dx.doi.org/10.1016/j.cardfail.2013.03.008) | Li, Yanhong, Neilson, Matthew P, Whellan, David J, Schulman, Kevin A, Levy, Wayne C. Associations between Seattle Heart Failure Model scores and health utilities: findings from HF-ACTION. Journal of cardiac failure 2013;19(5):311-16. | https://dx.doi.org/10.1016/j.cardfail.2013.03.008 |
| [Lindeman et al. (2018)](https://dx.doi.org/10.1097/SLA.0000000000002896) | Lindeman, Jan H N, Zwaginga, Jaap Jan, Kallenberg-Lantrua, Graziella, van Wissen, Rob C, Schepers, Abbey, van Bockel, Hajo J, Fibbe, Willem E. No Clinical Benefit of Intramuscular Delivery of Bone Marrow-derived Mononuclear Cells in Nonreconstructable Peripheral Arterial Disease: Results of a Phase-III Randomized-controlled Trial. Annals of surgery 2018;268(5):756-61. | https://dx.doi.org/10.1097/SLA.0000000000002896 |
| [Lindgren et al. (2017a)](https://dx.doi.org/10.1177/1179546817747528) | Lindgren, Hans Iv, Parsson, Hakan, Gottsater, Anders. Patients With Intermittent Claudication and Chronic Widespread Pain Improves in Health-Related Quality of Life After Invasive but Not After Noninvasive Treatment. Clinical Medicine Insights. Cardiology 2017;11():1179546817747528. | https://dx.doi.org/10.1177/1179546817747528 |
| [Lindgren et al. (2017b)](https://dx.doi.org/10.1177/1179546817747528) | Lindgren, H, Qvarfordt, P, Akesson, M, Bergman, S, Gottsater, A. Primary Stenting of the Superficial Femoral Artery in Intermittent Claudication Improves Health Related Quality of Life, ABI and Walking Distance: 12 Month Results of a Controlled Randomised Multicentre Trial. European journal of vascular and endovascular surgery : the official journal of the European Society for Vascular Surgery 2017;53(5):686-94. | https://dx.doi.org/10.1016/j.ejvs.2017.01.026 |
| [Liu et al. (2020)](http://dx.doi.org/10.1111/eci.13261) | Liu, Feng, Zhang, Han, Li, Yanming, Lu Feng. Hypocalcaemia predicts 12-month re-hospitalization in heart failure. European Journal of Clinical Investigation 2020;50(8):e13261. | http://dx.doi.org/10.1111/eci.13261 |
| [Logan et al. (2014)](https://dx.doi.org/10.3310/hta18290) | Logan, Philippa A, Darby, Janet, Gladman, John R F, Horne, Jane, Sach, Tracey H, Williams, Hywel C, et al. Rehabilitation aimed at improving outdoor mobility for people after stroke: a multicentre randomised controlled study (the Getting out of the House Study). Health technology assessment (Winchester, England) 2014;18(29):vii-113. | https://dx.doi.org/10.3310/hta18290 |
| [Lombardi et al. (2015)](https://dx.doi.org/10.1016/j.nut.2014.04.021) | Lombardi, Carlo, Carubelli, Valentina, Lazzarini, Valentina, Vizzardi, Enrico, Bordonali, Tania, Ciccarese, Camilla, et al. Effects of oral administration of orodispersible levo-carnosine on quality of life and exercise performance in patients with chronic heart failure. Nutrition (Burbank, Los Angeles County, Calif.) 2015;31(1):72-78. | https://dx.doi.org/10.1016/j.nut.2014.04.021 |
| [Lozano et al. (2013)](http://dx.doi.org/10.1016/j.angio.2013.01.012) | Lozano Sanchez, F S, March Garcia, J R, Carrasco Carrasco, E. Profile of patients with intermittent claudication in Spain. the VITAL Study. Angiologia 2013;65(4):131-40. | http://dx.doi.org/10.1016/j.angio.2013.01.012 |
| [Lozano et al. (2014a)](https://dx.doi.org/10.1111/ijcp.12499) | Lozano, F S, March, J R, Gonzalez-Porras, J R, Carrasco, E, Lobos, J M. Relative value of the Ankle-Brachial Index of intermittent claudication. International journal of clinical practice 2014;68(12):1478-82. | https://dx.doi.org/10.1111/ijcp.12499 |
| [Lozano et al. (2014b)](https://dx.doi.org/10.1111/ijcp.12499) | Lozano, Francisco S, Gonzalez-Porras, Jose R, March, Jose R, Carrasco, Eduardo, Lobos, Jose M. Differences between women and men with intermittent claudication: a cross-sectional study. Journal of women's health (2002) 2014;23(10):834-41. | https://dx.doi.org/10.1089/jwh.2013.4653 |
| [Lu et al. (2016)](https://dx.doi.org/10.2340/16501977-2069) | Lu, Wen-Shian, Huang, Sheau-Ling, Yang, Jeng-Feng, Chen, Mei-Hsiang, Hsieh, Ching-Lin. Convergent validity and responsiveness of the EQ-5D utility weights for stroke survivors. Journal of rehabilitation medicine 2016;48(4):346-51. | https://dx.doi.org/10.2340/16501977-2069 |
| [Luengo-Fernandez et al. (2013)](https://dx.doi.org/10.1212/WNL.0b013e3182a9f45f) | Luengo-Fernandez, Ramon, Gray, Alastair M, Bull, Linda, Welch, Sarah, Cuthbertson, Fiona, Rothwell, Peter M. Quality of life after TIA and stroke: ten-year results of the Oxford Vascular Study. Neurology 2013;81(18):1588-95. | https://dx.doi.org/10.1212/WNL.0b013e3182a9f45f |
| [Lunde (2013)](https://dx.doi.org/10.1007/s10198-012-0402-y) | Lunde, Lene. Can EQ-5D and 15D be used interchangeably in economic evaluations? Assessing quality of life in post-stroke patients. The European journal of health economics : HEPAC : health economics in prevention and care 2013;14(3):539-50. | https://dx.doi.org/10.1007/s10198-012-0402-y |
| [Luo et al. (2015)](https://dx.doi.org/10.1111/jgs.13796) | Luo, Juhua, Hendryx, Michael, Safford, Monika M, Wallace, Robert, Rossom, Rebecca, Eaton, Charles, Bassuk, Shari. Newly Developed Chronic Conditions and Changes in Health-Related Quality of Life in Postmenopausal Women. Journal of the American Geriatrics Society 2015;63(11):2349-57. | https://dx.doi.org/10.1111/jgs.13796 |
| [Lynch et al. (2020)](https://dx.doi.org/10.1080/09638288.2020.1852616) | Lynch, Elizabeth A, Labberton, Angela S, Kim, Joosup, Kilkenny, Monique F, Andrew, Nadine E, Lannin, Natasha A, et al. Out of sight, out of mind: long-term outcomes for people discharged home, to inpatient rehabilitation and to residential aged care after stroke. Disability and rehabilitation 2020;():1-7. | https://dx.doi.org/10.1080/09638288.2020.1852616 |
| [Magnuson et al. (2017)](https://dx.doi.org/10.1016/j.jacc.2017.05.063) | Magnuson, Elizabeth A, Li, Haiyan, Wang, Kaijun, Vilain, Katherine, Shafiq, Ali, Bonaca, Marc P, et al. Cost-Effectiveness of Long-Term Ticagrelor in Patients With Prior Myocardial Infarction: Results From the PEGASUS-TIMI 54 Trial. Journal of the American College of Cardiology 2017;70(5):527-38. | https://dx.doi.org/10.1016/j.jacc.2017.05.063 |
| [Magnuson et al. (2021)](http://dx.doi.org/10.1093/ehjqcco/qcab014) | Magnuson, Elizabeth A, Wang, Kaijun, Thomas, Merrill, Jones, Philip G, Cohen, David J, Arnold, Suzanne V, et al. Predicting the EQ-5D from the Kansas City Cardiomyopathy Questionnaire (KCCQ) in Patients with Heart Failure. European heart journal. Quality of care & clinical outcomes 2021;():n. pag.. | http://dx.doi.org/10.1093/ehjqcco/qcab014 |
| [Mahesh et al. (2018)](https://dx.doi.org/10.1007/s10072-017-3172-6) | Mahesh, P K B, Gunathunga, M W, Jayasinghe, S, Arnold, S M. Factors influencing pre-stroke and post-stroke quality of life among stroke survivors in a lower middle-income country. Neurological sciences : official journal of the Italian Neurological Society and of the Italian Society of Clinical Neurophysiology 2018;39(2):287-95. | https://dx.doi.org/10.1007/s10072-017-3172-6 |
| [Maksimovic et al. (2014)](https://dx.doi.org/10.1177/0003319713488640) | Maksimovic, Milos, Vlajinac, Hristina, Marinkovic, Jelena, Kocev, Nikola, Voskresenski, Tatjana. Health-related quality of life among patients with peripheral arterial disease. Angiology 2014;65(6):501-06. | https://dx.doi.org/10.1177/0003319713488640 |
| [Mar et al. (2015)](https://dx.doi.org/10.1186/s12955-015-0230-8) | Mar, Javier, Masjuan, Jaime, Oliva-Moreno, Juan, Gonzalez-Rojas, Nuria, Becerra, Virginia, Casado, Miguel Angel, et al. Outcomes measured by mortality rates, quality of life and degree of autonomy in the first year in stroke units in Spain. Health and quality of life outcomes 2015;13():36. | https://dx.doi.org/10.1186/s12955-015-0230-8 |
| [Marrett et al. (2013)](https://dx.doi.org/10.1186/1477-7525-11-175) | Marrett, Elizabeth & DiBonaventura, Marco daCosta. Burden of peripheral arterial disease in Europe and the United States: a patient survey. Health and quality of life outcomes 2013;11():175. | https://dx.doi.org/10.1186/1477-7525-11-175 |
| [Martins et al. (2021)](https://dx.doi.org/10.1080/10749357.2020.1805244) | Martins, Lais Geronutti, Molle da Costa, Rafael Dalle, Alvarez Sartor, Lorena Cristina, Thomaz de Souza, Juli, Winckler, Fernanda Cristina, Regina da Silva, Tais, et al. Clinical factors associated with trunk control after stroke: A prospective study. Topics in stroke rehabilitation 2021;28(3):181-89. | https://dx.doi.org/10.1080/10749357.2020.1805244 |
| [Martinson et al. (2017)](https://dx.doi.org/10.1002/ejhf.642) | Martinson, Melissa, Bharmi, Rupinder, Dalal, Nirav, Abraham, William T. Pulmonary artery pressure-guided heart failure management: US cost-effectiveness analyses using the results of the CHAMPION clinical trial. European journal of heart failure 2017;19(5):652-60. | https://dx.doi.org/10.1002/ejhf.642 |
| [Masciocco et al. (2013)](http://dx.doi.org/10.1159/000355169) | Masciocco, L, Benvenuto, A, Saracino, P, Massari, F, De Viti, D, Meliota, G, et al. Ivabradine improves quality of life in subjects with chronic heart failure compared to treatment with beta-blockers: results of a multicentric observational APULIA study. Pharmacology 2013;92(5-6):276-80. | http://dx.doi.org/10.1159/000355169 |
| [Matsumoto et al. (2016)](http://ovidsp.ovid.com/ovidweb.cgi?T=JS&PAGE=reference&D=med13&NEWS=N&AN=27996326) | Matsumoto, Shuji, Shimodozono, Megumi, Noma, Tomokazu, Uema, Tomohiro, Horio, Shinya, Tomioka, Kazutoshi, et al. Outcomes of repetitive facilitation exercises in convalescent patients after stroke with impaired health status. Brain injury 2016;30(13-14):1722-30. | http://ovidsp.ovid.com/ovidweb.cgi?T=JS&PAGE=reference&D=med13&NEWS=N&AN=27996326 |
| [Matza et al. (2015)](https://dx.doi.org/10.1186/s12913-015-0772-9) | Matza, Louis S, Stewart, Katie D, Gandra, Shravanthi R, Delio, Philip R, Fenster, Brett E, Davies, Evan W, et al. Acute and chronic impact of cardiovascular events on health state utilities. BMC health services research 2015;15():173. | https://dx.doi.org/10.1186/s12913-015-0772-9 |
| [Mayo et al. (2015a)](https://dx.doi.org/10.1007/s11136-013-0605-4) | Mayo, Nancy E, Scott, Susan C, Bayley, Mark, Cheung, Angela, Garland, Jayne, Jutai, Jeffrey. Modeling health-related quality of life in people recovering from stroke. Quality of life research : an international journal of quality of life aspects of treatment, care and rehabilitation 2015;24(1):41-53. | https://dx.doi.org/10.1007/s11136-013-0605-4 |
| [Mayo et al. (2015b)](https://dx.doi.org/10.1007/s11136-013-0605-4) | Mayo, Nancy E, Anderson, Sharon, Barclay, Ruth, Cameron, Jill I, Desrosiers, Johanne, Eng, Janice J, et al. Getting on with the rest of your life following stroke: a randomized trial of a complex intervention aimed at enhancing life participation post stroke. Clinical rehabilitation 2015;29(12):1198-2111. | https://dx.doi.org/10.1177/0269215514565396 |
| [McCreanor et al. (2021)](https://dx.doi.org/10.1136/bmjopen-2020-044054) | McCreanor, Victoria, Nowbar, Alexandra, Rajkumar, Christopher, Barnett, Adrian G, Francis, Darrel, Graves, Nicholas, et al. Cost-effectiveness analysis of percutaneous coronary intervention for single-vessel coronary artery disease: an economic evaluation of the ORBITA trial. BMJ open 2021;11(2):e044054. | https://dx.doi.org/10.1136/bmjopen-2020-044054 |
| [Mehralian et al. (2014)](https://dx.doi.org/10.5539/gjhs.v6n3p256) | Mehralian, Hossein, Salehi, Shahriar, Moghaddasi, Jafar, Amiri, Masoud. The comparison of the effects of education provided by nurses on the quality of life in patients with congestive heart failure (CHF) in usual and home-visit cares in Iran. Global journal of health science 2014;6(3):256-60. | https://dx.doi.org/10.5539/gjhs.v6n3p256 |
| [Mei et al. (2021)](https://dx.doi.org/10.1038/s41598-021-84554-6) | Mei, Yong-Xia, Wu, Hui, Zhang, Huan-Yun, Hou, Jian, Zhang, Zhen-Xiang, Liao, Wei, et al. Health-related quality of life and its related factors in coronary heart disease patients: results from the Henan Rural Cohort study. Scientific reports 2021;11(1):5011. | https://dx.doi.org/10.1038/s41598-021-84554-6 |
| [Meisinger et al. (2019)](http://dx.doi.org/10.1007/s11136-019-02306-6) | Meisinger, Christine, Kirchberger, Inge, Burkhardt, Katrin, Heier, Margit, Thilo Inge. Resilience is strongly associated with health-related quality of life but does not buffer work-related stress in employed persons 1 year after acute myocardial infarction. Quality of Life Research 2019;():n. pag.. | http://dx.doi.org/10.1007/s11136-019-02306-6 |
| [Mejhert & (2015)](https://dx.doi.org/10.3109/13814788.2014.908282) | Mejhert, Marit. A management programme for suspected heart failure in primary care in cooperation with specialists in cardiology. The European journal of general practice 2015;21(1):26-32. | https://dx.doi.org/10.3109/13814788.2014.908282 |
| [Mejia et al. (2014)](https://dx.doi.org/10.1016/j.ijnurstu.2014.01.009) | Mejia, Aurelio, Richardson, Gerry, Pattenden, Jill, Cockayne, Sarah. Cost-effectiveness of a nurse facilitated, cognitive behavioural self-management programme compared with usual care using a CBT manual alone for patients with heart failure: secondary analysis of data from the SEMAPHFOR trial. International journal of nursing studies 2014;51(9):1214-20. | https://dx.doi.org/10.1016/j.ijnurstu.2014.01.009 |
| [Min & (2015)](https://dx.doi.org/10.1093/ageing/afv060) | Min, Kyoung-Bok. Health-related quality of life is associated with stroke deficits in older adults. Age and ageing 2015;44(4):700-04. | https://dx.doi.org/10.1093/ageing/afv060 |
| [Miyahara et al. (2018)](https://dx.doi.org/10.1097/HCR.0000000000000296) | Miyahara, So, Fujimoto, Naoki, Dohi, Kaoru, Sugiura, Emiyo, Moriwaki, Keishi, Omori, Taku, et al. Postdischarge Light-Intensity Physical Activity Predicts Rehospitalization of Older Japanese Patients With Heart Failure. Journal of cardiopulmonary rehabilitation and prevention 2018;38(3):182-86. | https://dx.doi.org/10.1097/HCR.0000000000000296 |
| [Molle Da et al. (2019)](https://dx.doi.org/10.1080/10749357.2019.1631605) | Molle Da Costa, Rafael Dalle, Luvizutto, Gustavo Jose, Martins, Lais Geronutti, Thomaz De Souza, Juli, Regina Da Silva, Tais, Alvarez Sartor, Lorena Cristina, et al. Clinical factors associated with the development of nonuse learned after stroke: a prospective study. Topics in stroke rehabilitation 2019;26(7):511-17. | https://dx.doi.org/10.1080/10749357.2019.1631605 |
| [Monahan et al. (2017)](https://dx.doi.org/10.1016/j.ijcard.2017.02.149) | Monahan, Mark, Barton, Pelham, Taylor, Clare J, Roalfe, Andrea K, Hobbs, F D Richard, investigators, REFER, et al. MICE or NICE? An economic evaluation of clinical decision rules in the diagnosis of heart failure in primary care. International journal of cardiology 2017;241():255-61. | https://dx.doi.org/10.1016/j.ijcard.2017.02.149 |
| [Moren et al. (2016)](https://dx.doi.org/10.1097/NPT.0000000000000134) | Moren, Carina, Welmer, Anna-Karin, Hagstromer, Maria, Karlsson, Emelie. The Effects of 'Physical Activity on Prescription' in Persons With Transient Ischemic Attack: A Randomized Controlled Study. Journal of neurologic physical therapy : JNPT 2016;40(3):176-83. | https://dx.doi.org/10.1097/NPT.0000000000000134 |
| [Morey et al. (2021)](https://dx.doi.org/10.1161/CIRCOUTCOMES.120.006769) | Morey, Jacob R, Jiang, Shangqing, Klein, Sharon, Max, Wendy, Masharani, Umesh, Fleischmann, Kirsten E, Hunink, M G Myriam. Estimating Long-Term Health Utility Scores and Expenditures for Cardiovascular Disease From the Medical Expenditure Panel Survey. Circulation. Cardiovascular quality and outcomes 2021;():CIRCOUTCOMES120006769. | https://dx.doi.org/10.1161/CIRCOUTCOMES.120.006769 |
| [Morys et al. (2015)](https://dx.doi.org/10.5603/CJ.a2015.0027) | Morys, Joanna M, Hofer, Stefan, Rynkiewicz, Andrzej. The Polish MacNew heart disease heath-related quality of life questionnaire: a validation study. Cardiology journal 2015;22(5):541-50. | https://dx.doi.org/10.5603/CJ.a2015.0027 |
| [Muggah et al. (2013)](https://dx.doi.org/10.1186/1471-2458-13-16) | Muggah, Elizabeth, Graves, Erin, Bennett, Carol. Ascertainment of chronic diseases using population health data: a comparison of health administrative data and patient self-report. BMC public health 2013;13():16. | https://dx.doi.org/10.1186/1471-2458-13-16 |
| [Muli & (2013)](https://dx.doi.org/10.4314/ahs.v13i3.16) | Muli, G. Quality of life amongst young adults with stroke living in Kenya. African health sciences 2013;13(3):632-38. | https://dx.doi.org/10.4314/ahs.v13i3.16 |
| [Muller-Werdan et al. (2014)](http://ovidsp.ovid.com/ovidweb.cgi?T=JS&PAGE=reference&D=med11&NEWS=N&AN=25193811) | Muller-Werdan, Ursula, Stockl, Georg, Ebelt, Henning, Nuding, Sebastian, Hopfner, Florian, Werdan, Karl. Ivabradine in combination with beta-blocker reduces symptoms and improves quality of life in elderly patients with stable angina pectoris: age-related results from the ADDITIONS study. Experimental gerontology 2014;59():34-41. | http://ovidsp.ovid.com/ovidweb.cgi?T=JS&PAGE=reference&D=med11&NEWS=N&AN=25193811 |
| [Munyombwe et al. (2020)](https://dx.doi.org/10.1136/heartjnl-2019-315510) | Munyombwe, Theresa, Hall, Marlous, Dondo, Tatendashe Bernadette, Alabas, Oras A, Gerard, Oliver, West, Robert M, et al. Quality of life trajectories in survivors of acute myocardial infarction: a national longitudinal study. Heart (British Cardiac Society) 2020;106(1):33-39. | https://dx.doi.org/10.1136/heartjnl-2019-315510 |
| [Mustapha et al. (2019)](https://dx.doi.org/10.1177/1526602819827295) | Mustapha, Jihad, Gray, William, Martinsen, Brad J, Bolduan, Ryan W, Adams, George L, Ansel, Gary. One-Year Results of the LIBERTY 360 Study: Evaluation of Acute and Midterm Clinical Outcomes of Peripheral Endovascular Device Interventions. Journal of endovascular therapy : an official journal of the International Society of Endovascular Specialists 2019;26(2):143-54. | https://dx.doi.org/10.1177/1526602819827295 |
| [Nafees et al. (2014)](https://dx.doi.org/10.1016/j.jval.2014.08.1462) | Nafees, B, Cowie, M R, Patel, C, Deschaseaux, C, Brazier, J. Health State Utilities In Chronic Heart Failure In The Uk. Value in health : the journal of the International Society for Pharmacoeconomics and Outcomes Research 2014;17(7):A493. | https://dx.doi.org/10.1016/j.jval.2014.08.1462 |
| [Nagayama et al. (2017)](https://dx.doi.org/10.1080/10749357.2017.1289686) | Nagayama, Hirofumi, Tomori, Kounosuke, Ohno, Kanta, Takahashi, Kayoko, Nagatani, Ryutaro, Izumi, Ryota, Moriwaki, Kensuke. Cost effectiveness of the occupation-based approach for subacute stroke patients: result of a randomized controlled trial. Topics in stroke rehabilitation 2017;24(5):337-44. | https://dx.doi.org/10.1080/10749357.2017.1289686 |
| [Nam et al. (2015)](http://dx.doi.org/10.1186/s12962-015-0045-9) | Nam, Julian, Briggs, Andrew, Layland, Jamie, Oldroyd, Keith G, Eteiba, Hany, Petrie, Mark C, et al. Fractional flow reserve (FFR) versus angiography in guiding management to optimise outcomes in non-ST segment elevation myocardial infarction (FAMOUS-NSTEMI) developmental trial: Cost-effectiveness using a mixed trialand model-based methods. Cost Effectiveness and Resource Allocation 2015;13(1):19. | http://dx.doi.org/10.1186/s12962-015-0045-9 |
| [Nandal et al. (2021)](http://dx.doi.org/10.1111/imj.14749) | Nandal, Savvy, Chow, Chee Loong, Hannah, Vikki, Vaddadi, Gautam, Van Gaal Savvy. Tolerability and efficacy of sacubitril/valsartan in clinical practice. Internal Medicine Journal 2021;51(1):87-92. | http://dx.doi.org/10.1111/imj.14749 |
| [Nauck et al. (2019)](https://dx.doi.org/10.1111/dom.13547) | Nauck, Michael A, Buse, John B, Mann, Johannes F E, Pocock, Stuart, Bosch-Traberg, Heidrun, Frimer-Larsen, Helle, et al. Health-related quality of life in people with type 2 diabetes participating in the LEADER trial. Diabetes, obesity & metabolism 2019;21(3):525-32. | https://dx.doi.org/10.1111/dom.13547 |
| [Nicolau et al. (2020)](https://dx.doi.org/10.1002/clc.23476) | Nicolau, Jose C, Brieger, David, Owen, Ruth, Furtado, Remo H M, Goodman, Shaun G, Cohen, Mauricio G, et al. Diabetes association with self-reported health, resource utilization, and prognosis post-myocardial infarction. Clinical cardiology 2020;43(12):1352-61. | https://dx.doi.org/10.1002/clc.23476 |
| [Niewada et al. (2021)](http://dx.doi.org/10.33963/KP.15885) | Niewada, Maciej, Tabor, Bernadetta, Piotrowicz, Ewa, Piotrowicz, Ryszard, Opolski, Grzegorz, Banach, Maciej. Cost-effectiveness of telerehabilitation in patients with heart failure in Poland: an analysis based on the results of the Telerehabilitation in Heart Failure Patients (TELEREH-HF) randomized clinical trial. Kardiologia polska 2021;():n. pag.. | http://dx.doi.org/10.33963/KP.15885 |
| [Nikolic et al. (2013)](https://dx.doi.org/10.1093/eurheartj/ehs149) | Nikolic, Elisabet, Janzon, Magnus, Hauch, Ole, Wallentin, Lars, Henriksson, Martin. Cost-effectiveness of treating acute coronary syndrome patients with ticagrelor for 12 months: results from the PLATO study. European heart journal 2013;34(3):220-28. | https://dx.doi.org/10.1093/eurheartj/ehs149 |
| [Nolte et al. (2015)](https://dx.doi.org/10.1177/2047487314526071) | Nolte, Kathleen, Herrmann-Lingen, Christoph, Wachter, Rolf, Gelbrich, Gotz, Dungen, Hans-Dirk, Duvinage, Andre, et al. Effects of exercise training on different quality of life dimensions in heart failure with preserved ejection fraction: the Ex-DHF-P trial. European journal of preventive cardiology 2015;22(5):582-93. | https://dx.doi.org/10.1177/2047487314526071 |
| [Nordanstig et al. (2016)](https://dx.doi.org/10.1002/bjs.10198) | Nordanstig, J, Taft, C, Hensater, M, Perlander, A, Osterberg, K. Two-year results from a randomized clinical trial of revascularization in patients with intermittent claudication. The British journal of surgery 2016;103(10):1290-99. | https://dx.doi.org/10.1002/bjs.10198 |
| [Nozoe et al. (2021)](http://dx.doi.org/10.3390/ijerph18010251) | Nozoe, Masafumi, Mase, Kyoshi, Kanai, Masashi, Izawa, Kazuhiro P, Kubo, Hiroki, Shimada Kazuhiro P. Association of health utility score with physical activity outcomes in stroke survivors. International Journal of Environmental Research and Public Health 2021;18(1):1-9. | http://dx.doi.org/10.3390/ijerph18010251 |
| [Ock et al. (2016)](https://dx.doi.org/10.1186/s12889-016-2904-5) | Ock, Minsu, Jo, Min-Woo, Gong, Young-Hoon, Lee, Hyeon-Jeong, Lee, Jiho. Estimating the severity distribution of disease in South Korea using EQ-5D-3L: a cross-sectional study. BMC public health 2016;16():234. | https://dx.doi.org/10.1186/s12889-016-2904-5 |
| [Oemrawsingh et al. (2019)](https://dx.doi.org/10.1186/s12874-019-0864-z) | Oemrawsingh, Arvind, van Leeuwen, Nikki, Venema, Esmee, Limburg, Martien, de Leeuw, Frank-Erik, Wijffels, Markus P, et al. Value-based healthcare in ischemic stroke care: case-mix adjustment models for clinical and patient-reported outcomes. BMC medical research methodology 2019;19(1):229. | https://dx.doi.org/10.1186/s12874-019-0864-z |
| [Oh et al. (2017)](https://dx.doi.org/10.1016/j.wneu.2016.12.124) | Oh, Taemin, Lafage, Renaud, Lafage, Virginie, Protopsaltis, Themistocles, Challier, Vincent, Shaffrey, Christopher, et al. Comparing Quality of Life in Cervical Spondylotic Myelopathy with Other Chronic Debilitating Diseases Using the Short Form Survey 36-Health Survey. World neurosurgery 2017;106():699-706. | https://dx.doi.org/10.1016/j.wneu.2016.12.124 |
| [Ozyemisci-Taskiran et al. (2019)](https://dx.doi.org/10.1080/10749357.2018.1550957) | Ozyemisci-Taskiran, Ozden, Batur, Elif Balevi, Yuksel, Selcen, Cengiz, Mustafa. Validity and reliability of fatigue severity scale in stroke. Topics in stroke rehabilitation 2019;26(2):122-27. | https://dx.doi.org/10.1080/10749357.2018.1550957 |
| [Palmcrantz et al. (2014)](https://dx.doi.org/10.1186/1471-2377-14-20) | Palmcrantz, Susanne & Widen Holmqvist, Lotta. Young individuals with stroke: a cross sectional study of long-term disability associated with self-rated global health. BMC neurology 2014;14():20. | https://dx.doi.org/10.1186/1471-2377-14-20 |
| [Palsdottir et al. (2020a)](http://dx.doi.org/10.2340/16501977-2652) | Palsdottir, Anna Maria, Stigmar, Kjerstin, Norrving, Bo, Petersson, Ingemar F, Astrom, Mikael. The nature stroke study; NASTRU: A randomized controlled trial of nature-based post-stroke fatigue rehabilitation. Journal of rehabilitation medicine 2020;52(2):jrm00020. | https://dx.doi.org/10.2340/16501977-2652 |
| [Palsdottir et al. (2020b)](http://dx.doi.org/10.2340/16501977-2652) | Palsdottir, Anna-Maria, Stigmar, Kjerstin, Norrving, Bo, Petersson, Ingemar F, Astrom, Mikael. Nature-based rehabilitation to reduce post-stroke fatigue is not effective: A randomized controlled trial. Journal of rehabilitation medicine 2020;():n. pag.. | http://dx.doi.org/10.2340/16501977-2652 |
| [Pan et al. (2018)](https://dx.doi.org/10.1016/j.archger.2018.01.008) | Pan, Chen-Wei, Cong, Xiao-Ling, Zhou, Hui-Jun, Wang, Xing-Zhi, Sun, Hong-Peng, Xu, Yong. Evaluating health-related quality of life impact of chronic conditions among older adults from a rural town in Suzhou, China. Archives of gerontology and geriatrics 2018;76():6-11. | https://dx.doi.org/10.1016/j.archger.2018.01.008 |
| [Pavy et al. (2015)](https://dx.doi.org/10.1016/j.acvd.2014.09.006) | Pavy, Bruno, Iliou, Marie-Christine, Hofer, Stefan, Verges-Patois, Benedicte, Corone, Sonia, Aeberhard, Patrick, et al. Validation of the French version of the MacNew heart disease health-related quality of life questionnaire. Archives of cardiovascular diseases 2015;108(2):107-17. | https://dx.doi.org/10.1016/j.acvd.2014.09.006 |
| [Peng et al. (2019)](https://dx.doi.org/10.1016/j.archger.2019.04.011) | Peng, Li-Ning, Chen, Li-Ju, Lu, Wan-Hsuan, Tsai, Shu-Ling, Chen, Liang-Kung. Post-acute care regains quality of life among middle-aged and older stroke patients in Taiwan. Archives of gerontology and geriatrics 2019;83():271-76. | https://dx.doi.org/10.1016/j.archger.2019.04.011 |
| [Persson et al. (2017)](https://dx.doi.org/10.1186/s12955-017-0724-7) | Persson, Josefine, Levin, Lars-Ake, Holmegaard, Lukas, Redfors, Petra, Jood, Katarina, Jern, Christina, Blomstrand, Christian. Stroke survivors' long-term QALY-weights in relation to their spouses' QALY-weights and informal support: a cross-sectional study. Health and quality of life outcomes 2017;15(1):150. | https://dx.doi.org/10.1186/s12955-017-0724-7 |
| [Persson et al. (2020)](https://dx.doi.org/10.2147/COPD.S236192) | Persson, Hans Lennart & Lyth, Johan. The Health Diary Telemonitoring and Hospital-Based Home Care Improve Quality of Life Among Elderly Multimorbid COPD and Chronic Heart Failure Subjects. International journal of chronic obstructive pulmonary disease 2020;15():527-41. | https://dx.doi.org/10.2147/COPD.S236192 |
| [Peters-Klimm et al. (2013)](https://dx.doi.org/10.1177/1474515112439964) | Peters-Klimm, F, Freund, T, Kunz, C U, Laux, G, Frankenstein, L, Muller-Tasch, T. Determinants of heart failure self-care behaviour in community-based patients: a cross-sectional study. European journal of cardiovascular nursing : journal of the Working Group on Cardiovascular Nursing of the European Society of Cardiology 2013;12(2):167-76. | https://dx.doi.org/10.1177/1474515112439964 |
| [Petersohn et al. (2019)](https://dx.doi.org/10.1007/s11136-019-02166-0) | Petersohn, Svenja, Ramaekers, Bram L T, Olie, Renske H, Ten Cate-Hoek, Arina J, Daemen, Jan-Willem H C, Ten Cate, Hugo. Comparison of three generic quality-of-life metrics in peripheral arterial disease patients undergoing conservative and invasive treatments. Quality of life research : an international journal of quality of life aspects of treatment, care and rehabilitation 2019;28(8):2257-79. | https://dx.doi.org/10.1007/s11136-019-02166-0 |
| [Phan et al. (2019)](http://dx.doi.org/10.1161/STROKEAHA.118.024437) | Phan HT, Blizzard CL, Reeves MJ, et al. Sex Differences in Long-Term Quality of Life among Survivors after Stroke in the INSTRUCT. Stroke. 2019. | http://dx.doi.org/10.1161/STROKEAHA.118.024437 |
| [Phan et al. (2021)](https://dx.doi.org/10.1111/ene.14531) | Phan, H T, Gall, S L, Blizzard, C L, Lannin, N A, Thrift, A G, Anderson, C S, et al. Sex differences in quality of life after stroke were explained by patient factors, not clinical care: evidence from the Australian Stroke Clinical Registry. European journal of neurology 2021;28(2):469-78. | https://dx.doi.org/10.1111/ene.14531 |
| [Piotrowicz et al. (2015a)](https://dx.doi.org/10.1177/1474515114537023) | Piotrowicz, Ewa, Zielinski, Tomasz, Bodalski, Robert, Rywik, Tomasz, Dobraszkiewicz-Wasilewska, Barbara, Sobieszczanska-Malek, Malgorzata, et al. Home-based telemonitored Nordic walking training is well accepted, safe, effective and has high adherence among heart failure patients, including those with cardiovascular implantable electronic devices: a randomised controlled study. European journal of preventive cardiology 2015;22(11):1368-77. | https://dx.doi.org/10.1177/2047487314551537 |
| [Piotrowicz et al. (2015b)](https://dx.doi.org/10.1177/1474515114537023) | Piotrowicz, Ewa, Stepnowska, Monika, Leszczynska-Iwanicka, Kinga, Piotrowska, Dorota, Kowalska, Monika, Tylka, Jan, Piotrowski, Walerian. Quality of life in heart failure patients undergoing home-based telerehabilitation versus outpatient rehabilitation--a randomized controlled study. European journal of cardiovascular nursing : journal of the Working Group on Cardiovascular Nursing of the European Society of Cardiology 2015;14(3):256-63. | https://dx.doi.org/10.1177/1474515114537023 |
| [Pisa et al. (2015)](http://dx.doi.org/10.2147/PPA.S88167) | Pisa, Giovanni & Eichmann, Florian. Assessing patient preferences in heart failure using conjoint methodology. Patient Preference and Adherence 2015;9():1233-41. | http://dx.doi.org/10.2147/PPA.S88167 |
| [Pockett et al. (2018)](https://dx.doi.org/10.1080/13696998.2018.1454453) | Pockett, Rhys D, McEwan, Phil, Ray, Joshua, Tran, Irwin, Shutler, Simon, Martin, Steven, Yousef, Zaheer. Prospective utility study of patients with multiple cardiovascular events. Journal of medical economics 2018;21(6):616-21. | https://dx.doi.org/10.1080/13696998.2018.1454453 |
| [Pocock et al. (2021)](https://dx.doi.org/10.1136/openhrt-2020-001499) | Pocock, Stuart, Brieger, David B, Owen, Ruth, Chen, Jiyan, Cohen, Mauricio G, Goodman, Shaun, et al. Health-related quality of life 1-3 years post-myocardial infarction: its impact on prognosis. Open heart 2021;8(1):n. pag.. | https://dx.doi.org/10.1136/openhrt-2020-001499 |
| [Pogosova et al. (2014)](https://doi.org/10.20996/1819-6446-2014-10-6-584-596) | Pogosova, N V, Kursakov, A A, Boycharov, I H, Hofer, S. Validation of the MacNew questionnaire for the assessment of health-related quality of life in patients with ischemic heart disease. Rational Pharmacotherapy in Cardiology 2014;10(6):584-96. | https://doi.org/10.20996/1819-6446-2014-10-6-584-596 |
| [Pokharel et al. (2017)](https://dx.doi.org/10.1016/j.atherosclerosis.2017.09.019) | Pokharel, Yashashwi, Sharma, Puza P, Qintar, Mohammed, Lu, Yuan, Tang, Yuanyuan, Jones, Philip, Dreyer, Rachel P. High-sensitivity C-reactive protein levels and health status outcomes after myocardial infarction. Atherosclerosis 2017;266():16-23. | https://dx.doi.org/10.1016/j.atherosclerosis.2017.09.019 |
| [Poole et al. (2013)](https://dx.doi.org/10.1001/jama.2013.282540) | Poole, Joseph, Mavromatis, Kreton, Binongo, Jose N, Khan, Ali, Li, Qunna, Khayata, Mohamed, et al. Effect of progenitor cell mobilization with granulocyte-macrophage colony-stimulating factor in patients with peripheral artery disease: a randomized clinical trial. JAMA 2013;310(24):2631-39. | https://dx.doi.org/10.1001/jama.2013.282540 |
| [Prevost et al. (2015)](https://dx.doi.org/10.1177/2047487313512217) | Prevost, Alain, Lafitte, Marianne, Pucheu, Yann, Couffinhal, Thierry. Education and home based training for intermittent claudication: functional effects and quality of life. European journal of preventive cardiology 2015;22(3):373-79. | https://dx.doi.org/10.1177/2047487313512217 |
| [Prichard et al. (2021)](https://dx.doi.org/10.1007/s11136-020-02722-z) | Prichard, Roslyn A, Zhao, Fei-Li, Mcdonagh, Julee, Goodall, Stephen, Davidson, Patricia M, Newton, Phillip J, Farr-Wharton, Ben. Discrepancies between proxy estimates and patient reported, health related, quality of life: minding the gap between patient and clinician perceptions in heart failure. Quality of life research : an international journal of quality of life aspects of treatment, care and rehabilitation 2021;30(4):1049-59. | https://dx.doi.org/10.1007/s11136-020-02722-z |
| [Puumalainen et al. (2016)](https://dx.doi.org/10.1111/ane.12509) | Puumalainen, A, Numminen, H, Elonheimo, O, Roine, R O. Health outcomes and costs of ischemic stroke patients in Finland. Acta neurologica Scandinavica 2016;134(1):42-48. | https://dx.doi.org/10.1111/ane.12509 |
| [Pyo et al. (2017)](https://dx.doi.org/10.5535/arm.2017.41.6.935) | Pyo, Hannah, Kim, Bo Ra, Park, Mina, Hong, Jeong Hee. Effects of Overactive Bladder Symptoms in Stroke Patients' Health Related Quality of Life and Their Performance Scale. Annals of rehabilitation medicine 2017;41(6):935-43. | https://dx.doi.org/10.5535/arm.2017.41.6.935 |
| [Qvarfordt et al. (2018)](http://dx.doi.org/10.1007/s00270-018-1925-0) | Qvarfordt, Peter, Gottsater, Anders, Bergman, Stefan, Jansson, I, Litterfeldt, E, Lindgren, Hans I V, et al. Primary Stenting of the Superficial Femoral Artery in Patients with Intermittent Claudication Has Durable Effects on Health-Related Quality of Life at 24 Months: Results of a Randomized Controlled Trial. CardioVascular and Interventional Radiology 2018;41(6):872-81. | http://dx.doi.org/10.1007/s00270-018-1925-0 |
| [Rachpukdee et al. (2013)](https://dx.doi.org/10.1016/j.jstrokecerebrovasdis.2012.05.005) | Rachpukdee, Sangkaew, Howteerakul, Nopporn, Suwannapong, Nawarat. Quality of life of stroke survivors: a 3-month follow-up study. Journal of stroke and cerebrovascular diseases : the official journal of National Stroke Association 2013;22(7):e70-8. | https://dx.doi.org/10.1016/j.jstrokecerebrovasdis.2012.05.005 |
| [Raggi et al. (2016)](https://dx.doi.org/10.1371/journal.pone.0159293) | Raggi, Alberto, Corso, Barbara, Minicuci, Nadia, Quintas, Rui, Sattin, Davide, De Torres, Laura, et al. Determinants of Quality of Life in Ageing Populations: Results from a Cross-Sectional Study in Finland, Poland and Spain. PloS one 2016;11(7):e0159293. | https://dx.doi.org/10.1371/journal.pone.0159293 |
| [Rahmani et al. (2020)](https://dx.doi.org/10.1155/2020/8897881) | Rahmani, Ali, Vahedian-Azimi, Amir, Sirati-Nir, Masoud, Norouzadeh, Reza, Rozdar, Hamid. The Effect of the Teach-Back Method on Knowledge, Performance, Readmission, and Quality of Life in Heart Failure Patients. Cardiology research and practice 2020;2020():8897881. | https://dx.doi.org/10.1155/2020/8897881 |
| [Rajati et al. (2016)](https://dx.doi.org/10.1016/j.apmr.2016.05.010) | Rajati, Fatemeh, Feizi, Awat, Tavakol, Kamran, Mostafavi, Firoozeh, Sadeghi, Masoumeh. Comparative Evaluation of Health-Related Quality of Life Questionnaires in Patients With Heart Failure Undergoing Cardiac Rehabilitation: A Psychometric Study. Archives of physical medicine and rehabilitation 2016;97(11):1953-62. | https://dx.doi.org/10.1016/j.apmr.2016.05.010 |
| [Rancic et al. (2013)](http://dx.doi.org/10.2478/s11536-012-0118-5) | Rancic, Natasa K, Petrovic, Branislav D, Kocic, Biljana N, Ilic, Mirko V. Health-related quality of life in patients after the acute myocardial infarction. Central European Journal of Medicine 2013;8(2):266-72. | http://dx.doi.org/10.2478/s11536-012-0118-5 |
| [Rangaraju et al. (2016)](https://dx.doi.org/10.1159/000443801) | Rangaraju, Srikant & Frankel, Michael. Prognostic Value of the 24-Hour Neurological Examination in Anterior Circulation Ischemic Stroke: A post hoc Analysis of Two Randomized Controlled Stroke Trials. Interventional neurology 2016;4(3-4):120-29. | https://dx.doi.org/10.1159/000443801 |
| [Rangaraju et al. (2017)](https://dx.doi.org/10.1159/000452634) | Rangaraju, Srikant, Haussen, Diogo, Nogueira, Raul G, Nahab, Fadi. Comparison of 3-Month Stroke Disability and Quality of Life across Modified Rankin Scale Categories. Interventional neurology 2017;6(1-2):36-41. | https://dx.doi.org/10.1159/000452634 |
| [Rasmussen et al. (2019)](https://dx.doi.org/10.1097/JCN.0000000000000583) | Rasmussen, Trine Bernholdt, Palm, Pernille, Herning, Margrethe, Christensen, Anne Vinggaard, Borregaard, Britt, Nielsen, Kathrine Sjostedt Gandrup, et al. Subgroup Differences and Determinants of Patient-Reported Mental and Physical Health in Patients With Ischemic Heart Disease: Results From the DenHeart Study. The Journal of cardiovascular nursing 2019;34(4):E11-21. | https://dx.doi.org/10.1097/JCN.0000000000000583 |
| [Rasmussen et al. (2020a)](http://dx.doi.org/10.1093/ehjcvp/pvaa097) | Rasmussen, Anne A, Johnsen, Soren P, Berg, Selina K, Rasmussen, Trine B, Borregaard, Britt, Thrysoee, Lars, et al. Predictors of patient-reported outcomes at discharge in patients with heart failure. European journal of cardiovascular nursing : journal of the Working Group on Cardiovascular Nursing of the European Society of Cardiology 2020;19(8):748-56. | https://dx.doi.org/10.1177/1474515120902390 |
| [Rasmussen et al. (2020b)](http://dx.doi.org/10.1093/ehjcvp/pvaa097) | Rasmussen, Anne Ankerstjerne, Wiggers, Henrik, Mols, Rikke Elmose, Larsen, Signe Holm, Jensen, Martin, Johnsen, Soren Paaske, et al. Patient-Reported Outcomes and Medication Adherence in Patients with Heart Failure. European heart journal. Cardiovascular pharmacotherapy 2020;():n. pag.. | http://dx.doi.org/10.1093/ehjcvp/pvaa097 |
| [Rasmussen et al. (2020c)](http://dx.doi.org/10.1093/ehjcvp/pvaa097) | Rasmussen, Anne Ankerstjerne, Larsen, Signe Holm, Mols, Rikke Elmose, Wiggers, Henrik, Jensen, Martin, Johnsen, Soren Paaske, et al. Prognostic Impact of Self-Reported Health on Clinical Outcomes in Patients with Heart Failure. European heart journal. Quality of care & clinical outcomes 2020;():n. pag.. | http://dx.doi.org/10.1093/ehjqcco/qcaa026 |
| [Reed et al. (2013)](https://dx.doi.org/10.1016/j.cardfail.2013.07.003) | Reed, Shelby D, Kaul, Padma, Li, Yanhong, Eapen, Zubin J, Davidson-Ray, Linda, Schulman, Kevin A, et al. Medical resource use, costs, and quality of life in patients with acute decompensated heart failure: findings from ASCEND-HF. Journal of cardiac failure 2013;19(9):611-20. | https://dx.doi.org/10.1016/j.cardfail.2013.07.003 |
| [Renton et al. (2014)](http://dx.doi.org/10.1016/j.jstrokecerebrovasdis.2014.04.022) | Renton, Cheryl, Ellender, Sharon, Ankolekar, Sandeep, Sare, Gillian, Sprigg, Nikola, Bath, Philip M W, Wardlaw Nikola. Relationship between poststroke cognition, baseline factors, and functional outcome: Data from efficacy of nitric oxide in stroke trial. Journal of Stroke and Cerebrovascular Diseases 2014;23(7):1821-29. | http://dx.doi.org/10.1016/j.jstrokecerebrovasdis.2014.04.022 |
| [Rethnam et al. (2020)](https://dx.doi.org/10.1177/1747493019830583) | Rethnam, Venesha, Bernhardt, Julie, Dewey, Helen, Moodie, Marj, Johns, Hannah, Gao, Lan, et al. Utility-weighted modified Rankin Scale: Still too crude to be a truly patient-centric primary outcome measure?. International journal of stroke : official journal of the International Stroke Society 2020;15(3):268-77. | https://dx.doi.org/10.1177/1747493019830583 |
| [Reverte-Villarroya et al. (2020)](https://dx.doi.org/10.3390/ijerph17176014) | Reverte-Villarroya, Silvia, Davalos, Antoni, Font-Mayolas, Silvia, Berenguer-Poblet, Marta, Sauras-Colon, Esther, Lopez-Pablo, Carlos, et al. Coping Strategies, Quality of Life, and Neurological Outcome in Patients Treated with Mechanical Thrombectomy after an Acute Ischemic Stroke. International journal of environmental research and public health 2020;17(17):n. pag.. | https://dx.doi.org/10.3390/ijerph17176014 |
| [Rha et al. (2018)](https://dx.doi.org/10.4070/kcj.2017.0340) | Rha, Seung Woon, Choi, Seung Hyuk, Kim, Doo Il, Jeon, Dong Woon, Lee, Jae Hwan, Hong, Kyung Soon, et al. Medical Resource Consumption and Quality of Life in Peripheral Arterial Disease in Korea: PAD Outcomes (PADO) Research. Korean circulation journal 2018;48(9):813-25. | https://dx.doi.org/10.4070/kcj.2017.0340 |
| [Rhoda (2014)](https://dx.doi.org/10.4102/ajod.v3i1.126) | Rhoda, Anthea J. Health-related quality of life of patients six months poststroke living in the Western Cape, South Africa. African journal of disability 2014;3(1):126. | https://dx.doi.org/10.4102/ajod.v3i1.126 |
| [Rieckmann et al. (2020)](https://dx.doi.org/10.1186/s12955-020-01312-4) | Rieckmann, Nina, Neumann, Konrad, Feger, Sarah, Ibes, Paolo, Napp, Adriane, Preus, Daniel, et al. Health-related qualify of life, angina type and coronary artery disease in patients with stable chest pain. Health and quality of life outcomes 2020;18(1):140. | https://dx.doi.org/10.1186/s12955-020-01312-4 |
| [Robertson & (2016)](https://dx.doi.org/10.1016/j.cjca.2015.05.010) | Robertson, Alan J. A Randomized Controlled Trial of Allopurinol in Patients With Peripheral Arterial Disease. The Canadian journal of cardiology 2016;32(2):190-96. | https://dx.doi.org/10.1016/j.cjca.2015.05.010 |
| [Robles-Zurita et al. (2020)](https://dx.doi.org/10.1007/s10198-020-01235-3) | Robles-Zurita, Jose Antonio, Briggs, Andrew, Rana, Dikshyanta, Quayyum, Zahidul, Oldroyd, Keith G, Zeymer, Uwe, et al. Economic evaluation of culprit lesion only PCI vs. immediate multivessel PCI in acute myocardial infarction complicated by cardiogenic shock: the CULPRIT-SHOCK trial. The European journal of health economics : HEPAC : health economics in prevention and care 2020;21(8):1197-2009. | https://dx.doi.org/10.1007/s10198-020-01235-3 |
| [Rodriguez-Hernandez et al. (2021)](https://dx.doi.org/10.3390/ijerph18062810) | Rodriguez-Hernandez, Marta, Criado-Alvarez, Juan-Jose, Corregidor-Sanchez, Ana-Isabel, Martin-Conty, Jose L, Mohedano-Moriano, Alicia. Effects of Virtual Reality-Based Therapy on Quality of Life of Patients with Subacute Stroke: A Three-Month Follow-Up Randomized Controlled Trial. International journal of environmental research and public health 2021;18(6):n. pag.. | https://dx.doi.org/10.3390/ijerph18062810 |
| [Roffe et al. (2018)](https://dx.doi.org/10.3310/hta22140) | Roffe, Christine, Nevatte, Tracy, Bishop, Jon, Sim, Julius, Penaloza, Cristina, Jowett, Susan, et al. Routine low-dose continuous or nocturnal oxygen for people with acute stroke: three-arm Stroke Oxygen Supplementation RCT. Health technology assessment (Winchester, England) 2018;22(14):1-88. | https://dx.doi.org/10.3310/hta22140 |
| [Romero-Naranjo et al. (2019)](http://ovidsp.ovid.com/ovidweb.cgi?T=JS&PAGE=reference&D=med16&NEWS=N&AN=31260554) | Romero-Naranjo, Fernando, Espinosa-Uquillas, Carlos, Gordillo-Altamirano, Fernando. Which Factors may reduce the Health-Related Quality of Life of Ecuadorian Patients with Diabetes?. Puerto Rico health sciences journal 2019;38(2):102-08. | http://ovidsp.ovid.com/ovidweb.cgi?T=JS&PAGE=reference&D=med16&NEWS=N&AN=31260554 |
| [Rosic et al. (2021)](https://www.researchgate.net/publication/347937258_The_Role_of_Ambulatory_Cardiac_Rehabilitation_in_Improvement_of_Quality_of_Life_Anxiety_and_Depression) | Rosic, Damir, Krstacic, Goran, Krstacic, Antonija, Brborovic, Ognjen, Filipcic, Igor, Jelavic, Marko Mornar. The role of ambulatory cardiac rehabilitation in improvement of quality of life, anxiety and depression. Psychiatria Danubina 2021;32(Suppl 4):496-504. | https://www.researchgate.net/publication/347937258_The_Role_of_Ambulatory_Cardiac_Rehabilitation_in_Improvement_of_Quality_of_Life_Anxiety_and_Depression |
| [Rudberg et al. (2018)](https://dx.doi.org/10.1177/2396987317753444) | Rudberg, Ann-Sofie, Berge, Eivind, Gustavsson, Anders, Nasman, Per. Long-term health-related quality of life, survival and costs by different levels of functional outcome six months after stroke. European stroke journal 2018;3(2):157-64. | https://dx.doi.org/10.1177/2396987317753444 |
| [Sadlonova et al. (2021)](https://dx.doi.org/10.1016/j.jpsychores.2020.110353) | Sadlonova, Monika, Wasser, Katrin, Nagel, Jonas, Weber-Kruger, Mark, Groschel, Sonja, Uphaus, Timo, et al. Health-related quality of life, anxiety and depression up to 12 months post-stroke: Influence of sex, age, stroke severity and atrial fibrillation - A longitudinal subanalysis of the Find-AFRANDOMISED trial. Journal of psychosomatic research 2021;142():110353. | https://dx.doi.org/10.1016/j.jpsychores.2020.110353 |
| [Sahlen et al. (2016)](https://dx.doi.org/10.1177/0269216315618544) | Sahlen, Klas-Goran & Boman, Kurt. A cost-effectiveness study of person-centered integrated heart failure and palliative home care: Based on a randomized controlled trial. Palliative medicine 2016;30(3):296-302. | https://dx.doi.org/10.1177/0269216315618544 |
| [Salisbury et al. (2018)](https://dx.doi.org/10.1016/j.jvn.2017.09.003) | Salisbury, Dereck L, Whipple, Mary O, Burt, Marsha, Brown, Rebecca J L, Hirsch, Alan, Foley, Christopher. Translation of an evidence-based therapeutic exercise program for patients with peripheral artery disease. Journal of vascular nursing : official publication of the Society for Peripheral Vascular Nursing 2018;36(1):23-33. | https://dx.doi.org/10.1016/j.jvn.2017.09.003 |
| [Sanchez-Iriso et al. (2019)](https://dx.doi.org/10.1002/hec.3952) | Sanchez-Iriso, Eduardo & Errea Rodriguez, Maria. Valuing health using EQ-5D: The impact of chronic diseases on the stock of health. Health economics 2019;28(12):1402-17. | https://dx.doi.org/10.1002/hec.3952 |
| [Sand et al. (2016)](https://dx.doi.org/10.1111/ene.12848) | Sand, K M, Wilhelmsen, G, Naess, H, Midelfart, A, Thomassen, L. Vision problems in ischaemic stroke patients: effects on life quality and disability. European journal of neurology 2016;23 Suppl 1():1-7. | https://dx.doi.org/10.1111/ene.12848 |
| [Sandberg et al. (2016)](https://dx.doi.org/10.1016/j.apmr.2016.01.030) | Sandberg, Klas, Kleist, Marie, Falk, Lars, Enthoven, Paul, Sandberg, Klas, Falk, Lars, et al. Effects of Twice-Weekly Intense Aerobic Exercise in Early Subacute Stroke: A Randomized Controlled Trial. Archives of physical medicine and rehabilitation 2016;97(8):1244-53. | https://dx.doi.org/10.1016/j.apmr.2016.01.030 |
| [Sandercock (2013)](http://dx.doi.org/10.1016/S1474-4422%2813%2970130-3) | Sandercock, Peter. Effect of thrombolysis with alteplase within 6 h of acute ischaemic stroke on long-term outcomes (the third International Stroke Trial [IST-3]): 18-month follow-up of a randomised controlled trial. The Lancet Neurology 2013;12(8):768-76. | http://dx.doi.org/10.1016/S1474-4422%2813%2970130-3 |
| [Sandercock et al. (2013)](https://dx.doi.org/10.1016/S1474-4422(13)70130-3) | group, IST-3 collaborative. Effect of thrombolysis with alteplase within 6 h of acute ischaemic stroke on long-term outcomes (the third International Stroke Trial [IST-3]): 18-month follow-up of a randomised controlled trial. The Lancet. Neurology 2013;12(8):768-76. | https://dx.doi.org/10.1016/S1474-4422(13)70130-3 |
| [Sasaki et al. (2018)](https://dx.doi.org/10.1080/10749357.2018.1492775) | Sasaki, Shin, Kanai, Masashi, Shinoda, Taku, Morita, Hidemi, Shimada, Shinichi. Relation between health utility score and physical activity in community-dwelling ambulatory patients with stroke: a preliminary cross-sectional study. Topics in stroke rehabilitation 2018;():1-5. | https://dx.doi.org/10.1080/10749357.2018.1492775 |
| [Scherer et al. (2013)](https://dx.doi.org/10.1016/j.ejim.2013.01.003) | Scherer, Martin, Dungen, Hans-Dirk, Inkrot, Simone, Tahirovic, Elvis, Lashki, Diana Jahandar, Apostolovic, Svetlana, et al. Determinants of change in quality of life in the Cardiac Insufficiency Bisoprolol Study in Elderly (CIBIS-ELD). European journal of internal medicine 2013;24(4):333-38. | https://dx.doi.org/10.1016/j.ejim.2013.01.003 |
| [Schmid et al. (2013)](https://dx.doi.org/10.1111/j.1755-5922.2012.00313.x) | Schmid, Jean-Paul, Capoferri, Mauro, Wahl, Andreas, Eshtehardi, Parham. Cardiac shock wave therapy for chronic refractory angina pectoris. A prospective placebo-controlled randomized trial. Cardiovascular therapeutics 2013;31(3):e1-6. | https://dx.doi.org/10.1111/j.1755-5922.2012.00313.x |
| [Schneider et al. (2021)](https://dx.doi.org/10.1016/j.jstrokecerebrovasdis.2020.105499) | Schneider, Siim, Taba, Nele, Saapar, Minni, Vibo, Riina. Determinants of Long-Term Health-Related Quality of Life in Young Ischemic Stroke Patients. Journal of stroke and cerebrovascular diseases : the official journal of National Stroke Association 2021;30(2):105499. | https://dx.doi.org/10.1016/j.jstrokecerebrovasdis.2020.105499 |
| [Schowalter et al. (2013)](https://dx.doi.org/10.1007/s00392-012-0531-4) | Schowalter, Marion, Gelbrich, Gotz, Stork, Stefan, Langguth, Jan-Philip, Morbach, Caroline, Ertl, Georg, Faller, Hermann. Generic and disease-specific health-related quality of life in patients with chronic systolic heart failure: impact of depression. Clinical research in cardiology : official journal of the German Cardiac Society 2013;102(4):269-78. | https://dx.doi.org/10.1007/s00392-012-0531-4 |
| [Schreuders et al. (2017)](https://dx.doi.org/10.1177/1747493017706244) | Schreuders, Jennifer, van den Berg, Lucie A, Fransen, Puck Ss, Berkhemer, Olvert A, Beumer, Debbie, Lingsma, Hester F, et al. Quality of life after intra-arterial treatment for acute ischemic stroke in the MR CLEAN trial-Update. International journal of stroke : official journal of the International Stroke Society 2017;12(7):708-12. | https://dx.doi.org/10.1177/1747493017706244 |
| [Schulte et al. (2019)](https://dx.doi.org/10.1024/0301-1526/a000798) | Schulte, Karl-Ludwig, Hardung, David, Tiefenbacher, Christiane, Weiss, Thomas, Hoffmann, Ulrich, Amendt, Klaus, et al. Real-world outcomes of endovascular treatment in a non-selected population with peripheral artery disease - prospective study with 2-year follow-up. VASA. Zeitschrift fur Gefasskrankheiten 2019;48(5):433-41. | https://dx.doi.org/10.1024/0301-1526/a000798 |
| [Scuffham et al. (2017)](http://dx.doi.org/10.1016/j.ijcard.2016.11.030) | Scuffham, Paul A, Kularatna, Sanjeewa, Byrnes, Joshua, Chan, Yih Kai, Carrington, Melinda J, Stewart Sanjeewa, et al. Comparison of contemporaneous responses for EQ-5D-3L and Minnesota Living with Heart Failure; a case for disease specific multiattribute utility instrument in cardiovascular conditions. International Journal of Cardiology 2017;227():172-76. | http://dx.doi.org/10.1016/j.ijcard.2016.11.030 |
| [Seidel et al. (2014)](https://dx.doi.org/10.1371/journal.pone.0091176) | Seidel, Ulla K, Gronewold, Janine, Volsek, Michaela, Todica, Olga, Kribben, Andreas, Bruck, Heike. Physical, cognitive and emotional factors contributing to quality of life, functional health and participation in community dwelling in chronic kidney disease. PloS one 2014;9(3):e91176. | https://dx.doi.org/10.1371/journal.pone.0091176 |
| [Seidl et al. (2015)](https://dx.doi.org/10.1007/s10198-014-0623-3) | Seidl, Hildegard, Hunger, Matthias, Leidl, Reiner, Meisinger, Christa, Wende, Rupert, Kuch, Bernhard. Cost-effectiveness of nurse-based case management versus usual care for elderly patients with myocardial infarction: results from the KORINNA study. The European journal of health economics : HEPAC : health economics in prevention and care 2015;16(6):671-81. | https://dx.doi.org/10.1007/s10198-014-0623-3 |
| [Seidl et al. (2017)](https://dx.doi.org/10.1016/j.jval.2016.10.001) | Seidl, Hildegard, Hunger, Matthias, Meisinger, Christa, Kirchberger, Inge, Kuch, Bernhard, Leidl, Reiner. The 3-Year Cost-Effectiveness of a Nurse-Based Case Management versus Usual Care for Elderly Patients with Myocardial Infarction: Results from the KORINNA Follow-Up Study. Value in health : the journal of the International Society for Pharmacoeconomics and Outcomes Research 2017;20(3):441-50. | https://dx.doi.org/10.1016/j.jval.2016.10.001 |
| [Shao et al. (2019)](https://dx.doi.org/10.1007/s40273-019-00775-8) | Shao, Hui, Yang, Shuang, Fonseca, Vivian, Stoecker, Charles. Estimating Quality of Life Decrements Due to Diabetes Complications in the United States: The Health Utility Index (HUI) Diabetes Complication Equation. PharmacoEconomics 2019;37(7):921-29. | https://dx.doi.org/10.1007/s40273-019-00775-8 |
| [Shawo et al. (2020)](http://dx.doi.org/10.3310/hta24240) | Shawo, Lisa, Franciso, Richard, Hillso, Katie, Bhattaraio, Nawaraj, Howelo, Denise, Stampo, Elaine, et al. An extended stroke rehabilitation service for people who have had a stroke: The extras rct. Health Technology Assessment 2020;24(24):1-202. | http://dx.doi.org/10.3310/hta24240 |
| [Shireman et al. (2017)](https://dx.doi.org/10.1161/STROKEAHA.116.014735) | Shireman, Theresa I, Wang, Kaijun, Saver, Jeffrey L, Goyal, Mayank, Bonafe, Alain, Diener, Hans-Christoph, et al. Cost-Effectiveness of Solitaire Stent Retriever Thrombectomy for Acute Ischemic Stroke: Results From the SWIFT-PRIME Trial (Solitaire With the Intention for Thrombectomy as Primary Endovascular Treatment for Acute Ischemic Stroke). Stroke 2017;48(2):379-87. | https://dx.doi.org/10.1161/STROKEAHA.116.014735 |
| [Silva et al. (2020)](https://dx.doi.org/10.1080/10749357.2020.1864964) | Silva, Leonardo Carvalho, Silva, Andressa, Rangel, Marcela Ferreira De Andrade, Caetano, Livia Cristina Guimaraes, Teixeira-Salmela, Luci Fuscaldi. Depressive symptoms and functional status are associated with sleep quality after stroke. Topics in stroke rehabilitation 2020;():1-8. | https://dx.doi.org/10.1080/10749357.2020.1864964 |
| [Simpson et al. (2015)](https://dx.doi.org/10.12715/har.2015.4.4) | Simpson, Annie N & Simpson, Kit N. Health-related quality of life in older adults: Effects of hearing loss and common chronic conditions. Healthy aging research 2015;4():n. pag.. | https://dx.doi.org/10.12715/har.2015.4.4 |
| [Smolderen et al. (2015)](https://dx.doi.org/10.1161/JAHA.114.001424) | Smolderen, Kim G, Strait, Kelly M, Dreyer, Rachel P, D'Onofrio, Gail, Zhou, Shengfan, Lichtman, Judith H, et al. Depressive symptoms in younger women and men with acute myocardial infarction: insights from the VIRGO study. Journal of the American Heart Association 2015;4(4):n. pag.. | https://dx.doi.org/10.1161/JAHA.114.001424 |
| [Sobajima et al. (2015)](https://dx.doi.org/10.1536/ihj.14-266) | Sobajima, Mitsuo, Nozawa, Takashi, Fukui, Yasutaka, Ihori, Hiroyuki, Ohori, Takashi, Fujii, Nozomu. Waon therapy improves quality of life as well as cardiac function and exercise capacity in patients with chronic heart failure. International heart journal 2015;56(2):203-08. | https://dx.doi.org/10.1536/ihj.14-266 |
| [Sprigg et al. (2013)](https://dx.doi.org/10.1161/STROKEAHA.113.002201) | Sprigg, Nikola, Selby, James, Fox, Lydia, Berge, Eivind, Whynes, David, Bath, Philip M W. Very low quality of life after acute stroke: data from the Efficacy of Nitric Oxide in Stroke trial. Stroke 2013;44(12):3458-62. | https://dx.doi.org/10.1161/STROKEAHA.113.002201 |
| [Squire et al. (2017)](http://dx.doi.org/10.5837/bjc.2017.007) | Squire, Lain, Glover, Jason, Corp, Jacqueline, Haroun, Rola, Kuzan, David. Impact of HF on HRQoL in patients and their caregivers in England: Results from the ASSESS study. British Journal of Cardiology 2017;24(1):30-34. | http://dx.doi.org/10.5837/bjc.2017.007 |
| [Stauber et al. (2013)](https://dx.doi.org/10.1177/1358863X13505861) | Stauber, Stefanie, Guera, Viviane, Barth, Jurgen, Schmid, Jean Paul, Saner, Hugo, Znoj, Hansjorg, Grolimund, Johannes. Psychosocial outcome in cardiovascular rehabilitation of peripheral artery disease and coronary artery disease patients. Vascular medicine (London, England) 2013;18(5):257-62. | https://dx.doi.org/10.1177/1358863X13505861 |
| [Stehlik et al. (2017)](http://dx.doi.org/10.1161/CIRCHEARTFAILURE.116.003910) | Stehlik, Josef, Selzman, Craig H, Estep, Jerry D, Rogers, Joseph G, Spertus, John A, Shah, Keyur B, et al. Patient-Reported Health-Related Quality of Life Is a Predictor of Outcomes in Ambulatory Heart Failure Patients Treated with Left Ventricular Assist Device Compared with Medical Management: Results from the ROADMAP Study (Risk Assessment and Comparative E. Circulation: Heart Failure 2017;10(6):e003910. | http://dx.doi.org/10.1161/CIRCHEARTFAILURE.116.003910 |
| [Stewart et al. (2016)](http://ovidsp.ovid.com/ovidweb.cgi?T=JS&PAGE=reference&D=med13&NEWS=N&AN=27834198) | Stewart, Garrick C, Kittleson, Michelle M, Patel, Parag C, Cowger, Jennifer A, Patel, Chetan B, Mountis, Maria M, et al. INTERMACS (Interagency Registry for Mechanically Assisted Circulatory Support) Profiling Identifies Ambulatory Patients at High Risk on Medical Therapy After Hospitalizations for Heart Failure. Circulation. Heart failure 2016;9(11):n. pag.. | http://ovidsp.ovid.com/ovidweb.cgi?T=JS&PAGE=reference&D=med13&NEWS=N&AN=27834198 |
| [Stojanovic et al. (2018)](https://dx.doi.org/10.21101/cejph.a5022) | Stojanovic, Miodrag, Cvetanovic, Goran, Andelkovic Apostolovic, Marija, Stojanovic, Dijana. Impact of socio-demographic characteristics and long-term complications on quality of life in patients with diabetes mellitus. Central European journal of public health 2018;26(2):104-10. | https://dx.doi.org/10.21101/cejph.a5022 |
| [Stolz et al. (2019)](https://dx.doi.org/10.1097/MRR.0000000000000375) | Stolz, Robert, Nayyar, Rohini, Louie, Julie, Bower, Kelly J, Paul, Sanjoy K. The effectiveness of a novel cable-driven gait trainer (Robowalk) combined with conventional physiotherapy compared to conventional physiotherapy alone following stroke: a randomised controlled trial. International journal of rehabilitation research. Internationale Zeitschrift fur Rehabilitationsforschung. Revue internationale de recherches de readaptation 2019;42(4):377-84. | https://dx.doi.org/10.1097/MRR.0000000000000375 |
| [Stone et al. (2016)](https://dx.doi.org/10.1093/eurheartj/ehv436) | Stone, Gregg W, Chung, Eugene S, Stancak, Branislav, Svendsen, Jesper H, Fischer, Trent M, Kueffer, Fred, et al. Peri-infarct zone pacing to prevent adverse left ventricular remodelling in patients with large myocardial infarction. European heart journal 2016;37(5):484-93. | https://dx.doi.org/10.1093/eurheartj/ehv436 |
| [Stummer et al. (2015)](https://dx.doi.org/10.3109/09638288.2014.948137) | Stummer, C, Verheyden, G, Putman, K, Jenni, W, Schupp, W. Predicting sickness impact profile at six months after stroke: further results from the European multi-center CERISE study. Disability and rehabilitation 2015;37(11):942-50. | https://dx.doi.org/10.3109/09638288.2014.948137 |
| [Sun et al. (2019)](https://dx.doi.org/10.12998/wjcc.v7.i16.2165) | Sun, Jing, Zhang, Zhi-Wei, Ma, Yue-Xian, Liu, Wei. Application of self-care based on full-course individualized health education in patients with chronic heart failure and its influencing factors. World journal of clinical cases 2019;7(16):2165-75. | https://dx.doi.org/10.12998/wjcc.v7.i16.2165 |
| [Szocs et al. (2020)](https://dx.doi.org/10.1371/journal.pone.0241059) | Szocs, Ildiko, Dobi, Balazs, Lam, Judit, Orban-Kis, Karoly, Hakkinen, Unto, Belicza, Eva, Bereczki, Daniel. Health related quality of life and satisfaction with care of stroke patients in Budapest: A substudy of the EuroHOPE project. PloS one 2020;15(10):e0241059. | https://dx.doi.org/10.1371/journal.pone.0241059 |
| [Szygula-Jurkiewicz et al. (2014)](https://dx.doi.org/10.5114/kitp.2014.45678) | Szygula-Jurkiewicz, Bozena, Zakliczynski, Michal, Owczarek, Aleksander, Partyka, Robert, Moscinski, Mateusz, Pudlo, Robert, et al. Low health-related quality of life is a predictor of major adverse cardiovascular events in patients with chronic nonischemic heart failure. Kardiochirurgia i torakochirurgia polska = Polish journal of cardio-thoracic surgery 2014;11(3):283-88. | https://dx.doi.org/10.5114/kitp.2014.45678 |
| [Tasic et al. (2014)](http://dx.doi.org/10.2478/s11536-013-0186-1) | Tasic, Ivan, Kostic, Svetlana, Djordjevic, Dragan, Simonovic, Dejan, Rihter, Marija, Lazarevic, Gordana, Vulic, Dusan. Impact of depression on sexual dysfunction and HRQoL in CAD patients. Central European Journal of Medicine 2014;9(1):54-63. | http://dx.doi.org/10.2478/s11536-013-0186-1 |
| [Teng et al. (2018)](https://dx.doi.org/10.1177/1474515118778453) | Teng, Hsiu-Chin & Yeh, Mei-Ling. Walking with controlled breathing improves exercise tolerance, anxiety, and quality of life in heart failure patients: A randomized controlled trial. European journal of cardiovascular nursing : journal of the Working Group on Cardiovascular Nursing of the European Society of Cardiology 2018;17(8):717-27. | https://dx.doi.org/10.1177/1474515118778453 |
| [Thrift et al. (2019)](http://dx.doi.org/10.1016/j.jstrokecerebrovasdis.2019.01.026) | Thrift, Amanda G, Cadilhac, Dominique A, Kilkenny, Monique F, Grabsch, Brenda, Donnan, Geoffrey A, Lannin, Natasha A, et al. Outcomes for Patients With In-Hospital Stroke: A Multicenter Study From the Australian Stroke Clinical Registry (AuSCR). Journal of Stroke and Cerebrovascular Diseases 2019;28(5):1302-10. | http://dx.doi.org/10.1016/j.jstrokecerebrovasdis.2019.01.026 |
| [Timmermans et al. (2014)](https://dx.doi.org/10.1186/1743-0003-11-45) | Timmermans, Annick A A, Lemmens, Ryanne J M, Monfrance, Maurice, Geers, Richard P J, Bakx, Wilbert, Smeets, Rob J E M. Effects of task-oriented robot training on arm function, activity, and quality of life in chronic stroke patients: a randomized controlled trial. Journal of neuroengineering and rehabilitation 2014;11():45. | https://dx.doi.org/10.1186/1743-0003-11-45 |
| [Timoteo et al. (2020)](https://dx.doi.org/10.1016/j.repc.2019.09.013) | Timoteo, Ana Teresa, Dias, Sara Simoes, Rodrigues, Ana Maria, Gregorio, Maria Joao, Sousa, Rute Dinis. Quality of life in adults living in the community with previous self-reported myocardial infarction. Revista portuguesa de cardiologia : orgao oficial da Sociedade Portuguesa de Cardiologia = Portuguese journal of cardiology : an official journal of the Portuguese Society of Cardiology 2020;39(7):367-73. | https://dx.doi.org/10.1016/j.repc.2019.09.013 |
| [Toell et al. (2020)](http://dx.doi.org/10.1016/j.eclinm.2020.100476) | Toell, Thomas, Boehme, Christian, Mayer, Lukas, Seekircher, Lisa, Tschiderer, Lena, Schoenherr, Gudrun, et al. STROKE-CARD care to prevent cardiovascular events and improve quality of life after acute ischaemic stroke or TIA: A randomised clinical trial. EClinicalMedicine 2020;25():100476. | http://dx.doi.org/10.1016/j.eclinm.2020.100476 |
| [Trystula (2018)](http://dx.doi.org/10.5604/01.3001.0011.7065) | Trystula, Mariusz. Health related quality of life for patients after a transient ischaemic attack: Is carotid endarterectomy (CEA) or carotid artery stenting (CAS) more influential? Acta Neuropsychologica 2018;16(1):61-68. | http://dx.doi.org/10.5604/01.3001.0011.7065 |
| [Tsai et al. (2019)](https://dx.doi.org/10.6515/ACS.201907_35(4).20190330A) | Tsai, Yun-Jeng, Huang, Wei-Chun, Weng, Tzu-Pin. Early Phase II Comprehensive Cardiac Rehabilitation after Acute Myocardial Infarction. Acta Cardiologica Sinica 2019;35(4):425-29. | https://dx.doi.org/10.6515/ACS.201907_35(4).20190330A |
| [Turkstra et al. (2013)](https://dx.doi.org/10.1186/1471-2261-13-33) | Turkstra, Erika, Hawkes, Anna L, Oldenburg, Brian. Cost-effectiveness of a coronary heart disease secondary prevention program in patients with myocardial infarction: results from a randomised controlled trial (ProActive Heart). BMC cardiovascular disorders 2013;13():33. | https://dx.doi.org/10.1186/1471-2261-13-33 |
| [Tusek-Bunc & (2016)](http://ovidsp.ovid.com/ovidweb.cgi?T=JS&PAGE=reference&D=med13&NEWS=N&AN=27846850) | Tusek-Bunc, Ksenija. Comorbidities and characteristics of coronary heart disease patients: their impact on health-related quality of life. Health and quality of life outcomes 2016;14(1):159. | http://ovidsp.ovid.com/ovidweb.cgi?T=JS&PAGE=reference&D=med13&NEWS=N&AN=27846850 |
| [Vahedian-Azimi et al. (2016)](https://dx.doi.org/10.1136/openhrt-2015-000349) | Vahedian-Azimi, Amir, Miller, Andrew C, Hajiesmaieli, Mohammadreza, Kangasniemi, Mari, Alhani, Fatemah, Jelvehmoghaddam, Hosseinali, et al. Cardiac rehabilitation using the Family-Centered Empowerment Model versus home-based cardiac rehabilitation in patients with myocardial infarction: a randomised controlled trial. Open heart 2016;3(1):e000349. | https://dx.doi.org/10.1136/openhrt-2015-000349 |
| [Vaidya et al. (2018)](https://dx.doi.org/10.2217/cer-2017-0029) | Vaidya, Anil, Kleinegris, Marie-Claire, Severens, Johan L, Ramaekers, Bram L, Ten Cate-Hoek, Arina J, Ten Cate, Hugo. Comparison of EQ-5D and SF-36 in untreated patients with symptoms of intermittent claudication. Journal of comparative effectiveness research 2018;7(6):535-48. | https://dx.doi.org/10.2217/cer-2017-0029 |
| [van den et al. (2017)](https://dx.doi.org/10.1056/NEJMoa1612136) | van den Berg, Lucie A, Dijkgraaf, Marcel G W, Berkhemer, Olvert A, Fransen, Puck S S, Beumer, Debbie, Lingsma, Hester F, et al. Two-Year Outcome after Endovascular Treatment for Acute Ischemic Stroke. The New England journal of medicine 2017;376(14):1341-49. | https://dx.doi.org/10.1056/NEJMoa1612136 |
| [van et al. (2015)](https://dx.doi.org/10.1136/bmjopen-2015-008220) | van Eeden, M, van Heugten, C, van Mastrigt, G A P G, van Mierlo, M, Visser-Meily, J M A. The burden of stroke in the Netherlands: estimating quality of life and costs for 1 year poststroke. BMJ open 2015;5(11):e008220. | https://dx.doi.org/10.1136/bmjopen-2015-008220 |
| [Van et al. (2019)](https://dx.doi.org/10.1001/jama.2019.0710) | Van Spall, Harriette G C, Lee, Shun Fu, Xie, Feng, Oz, Urun Erbas, Perez, Richard, Mitoff, Peter R, et al. Effect of Patient-Centered Transitional Care Services on Clinical Outcomes in Patients Hospitalized for Heart Failure: The PACT-HF Randomized Clinical Trial. JAMA 2019;321(8):753-61. | https://dx.doi.org/10.1001/jama.2019.0710 |
| [van et al. (2020)](https://dx.doi.org/10.1186/s12913-020-05103-x) | van Mastrigt, Ghislaine A P G, van Eeden, Mitchel, van Heugten, Caroline M, Tielemans, Nienke, Schepers, Vera P M. A trial-based economic evaluation of the Restore4Stroke self-management intervention compared to an education-based intervention for stroke patients and their partners. BMC health services research 2020;20(1):294. | https://dx.doi.org/10.1186/s12913-020-05103-x |
| [Varnfield et al. (2014)](https://dx.doi.org/10.1136/heartjnl-2014-305783) | Varnfield, Marlien, Karunanithi, Mohanraj, Lee, Chi-Keung, Honeyman, Enone, Arnold, Desre, Ding, Hang, Smith, Catherine. Smartphone-based home care model improved use of cardiac rehabilitation in postmyocardial infarction patients: results from a randomised controlled trial. Heart (British Cardiac Society) 2014;100(22):1770-79. | https://dx.doi.org/10.1136/heartjnl-2014-305783 |
| [Venkataraman et al. (2013)](https://dx.doi.org/10.1111/j.1365-2265.2012.04480.x) | Venkataraman, K, Wee, H L, Leow, M K S, Tai, E S, Lee, J, Lim, S C, et al. Associations between complications and health-related quality of life in individuals with diabetes. Clinical endocrinology 2013;78(6):865-73. | https://dx.doi.org/10.1111/j.1365-2265.2012.04480.x |
| [Verberne et al. (2021)](https://dx.doi.org/10.1136/bmjopen-2020-039201) | Verberne, Daan P J, van Mastrigt, Ghislaine A P G, Ponds, Rudolf W H M, van Heugten, Caroline M. Economic evaluation of nurse-led stroke aftercare addressing long-term psychosocial outcome: a comparison to care-as-usual. BMJ open 2021;11(2):e039201. | https://dx.doi.org/10.1136/bmjopen-2020-039201 |
| [Vicent et al. (2017)](https://dx.doi.org/10.1186/s12904-017-0208-x) | Vicent, Lourdes, Nunez Olarte, Juan Manuel, Puente-Maestu, Luis, Oliva, Alicia, Lopez, Juan Carlos, Postigo, Andrea, et al. Degree of dyspnoea at admission and discharge in patients with heart failure and respiratory diseases. BMC palliative care 2017;16(1):35. | https://dx.doi.org/10.1186/s12904-017-0208-x |
| [Villano et al. (2013)](https://dx.doi.org/10.1016/j.amjcard.2013.02.045) | Villano, Angelo, Di Franco, Antonino, Nerla, Roberto, Sestito, Alfonso, Tarzia, Pierpaolo, Lamendola, Priscilla, et al. Effects of ivabradine and ranolazine in patients with microvascular angina pectoris. The American journal of cardiology 2013;112(1):8-13. | https://dx.doi.org/10.1016/j.amjcard.2013.02.045 |
| [Visser et al. (2015)](https://dx.doi.org/10.1016/j.apmr.2015.04.007) | Visser, Marieke M, Heijenbrok-Kal, Majanka H, Spijker, Adriaan Van't, Oostra, Kristine M, Busschbach, Jan J. Coping, problem solving, depression, and health-related quality of life in patients receiving outpatient stroke rehabilitation. Archives of physical medicine and rehabilitation 2015;96(8):1492-98. | https://dx.doi.org/10.1016/j.apmr.2015.04.007 |
| [Visser et al. (2016)](https://dx.doi.org/10.1161/STROKEAHA.115.010961) | Visser, Marieke M, Heijenbrok-Kal, Majanka H, Van't Spijker, Adriaan, Lannoo, Engelien, Busschbach, Jan J V. Problem-Solving Therapy During Outpatient Stroke Rehabilitation Improves Coping and Health-Related Quality of Life: Randomized Controlled Trial. Stroke 2016;47(1):135-42. | https://dx.doi.org/10.1161/STROKEAHA.115.010961 |
| [Vlajinac et al. (2014)](https://dx.doi.org/10.1007/s00508-014-0663-9) | Vlajinac, Hristina, Marinkovic, Jelena, Tanaskovic, Slobodan, Kocev, Nikola, Radak, Djordje, Davidovic, Dragana, et al. Quality of life after peripheral bypass surgery: a 1 year follow-up. Wiener klinische Wochenschrift 2014;127(5-6):210-17. | https://dx.doi.org/10.1007/s00508-014-0663-9 |
| [Waehler et al. (2021)](https://dx.doi.org/10.1186/s12883-021-02128-5) | Waehler, Idunn Snorresdatter, Saltvedt, Ingvild, Lydersen, Stian, Fure, Brynjar, Askim, Torunn, Einstad, Marte Stine. Association between in-hospital frailty and health-related quality of life after stroke: the Nor-COAST study. BMC neurology 2021;21(1):100. | https://dx.doi.org/10.1186/s12883-021-02128-5 |
| [Walker et al. (2021)](https://dx.doi.org/10.1136/heartjnl-2020-316990) | Walker, Simon, Cox, Edward, Rothwell, Ben, Berry, Colin, McCann, Gerry P, Bucciarelli-Ducci, Chiara, et al. Cost-effectiveness of cardiovascular imaging for stable coronary heart disease. Heart (British Cardiac Society) 2021;107(5):381-88. | https://dx.doi.org/10.1136/heartjnl-2020-316990 |
| [Wallace et al. (2020)](https://dx.doi.org/10.1177/1545968319887682) | Wallace, Amanda Claire, Talelli, Penelope, Crook, Lucinda, Austin, Duncan, Farrell, Rachel, Hoad, Damon, et al. Exploratory Randomized Double-Blind Placebo-Controlled Trial of Botulinum Therapy on Grasp Release After Stroke (PrOMBiS). Neurorehabilitation and neural repair 2020;34(1):51-60. | https://dx.doi.org/10.1177/1545968319887682 |
| [Wang et al. (2014a)](https://dx.doi.org/10.1177/2047487312454757) | Wang, Wenru, Thompson, David R, Ski, Chantal F. Health-related quality of life and its associated factors in Chinese myocardial infarction patients. European journal of preventive cardiology 2014;21(3):321-29. | https://dx.doi.org/10.1177/2047487312454757 |
| [Wang et al. (2014b)](https://dx.doi.org/10.1177/2047487312454757) | Wang, Yi-Long, Pan, Yue-Song, Zhao, Xing-Quan, Wang, David, Johnston, S Claiborne, Liu, Li-Ping, et al. Recurrent stroke was associated with poor quality of life in patients with transient ischemic attack or minor stroke: finding from the CHANCE trial. CNS neuroscience & therapeutics 2014;20(12):1029-35. | https://dx.doi.org/10.1111/cns.12329 |
| [Wang et al. (2015a)](https://dx.doi.org/10.1007/s11239-015-1191-9) | Wang, Ling, Wu, Yi-Qun, Tang, Xun, Li, Na, He, Liu, Cao, Yang, Chen, Da-Fang. Profile and Correlates of Health-related Quality of Life in Chinese Patients with Coronary Heart Disease. Chinese medical journal 2015;128(14):1853-61. | https://dx.doi.org/10.4103/0366-6999.160486 |
| [Wang et al. (2015b)](https://dx.doi.org/10.1007/s11239-015-1191-9) | Wang, Ye, Xie, Feng, Kong, Ming Chai, Lee, Lai Heng, Ng, Heng Joo. Patient-reported health preferences of anticoagulant-related outcomes. Journal of thrombosis and thrombolysis 2015;40(3):268-73. | https://dx.doi.org/10.1007/s11239-015-1191-9 |
| [Wang et al. (2020a)](https://dx.doi.org/10.18632/aging.103288) | Wang, Xia, Moullaali, Tom J, Li, Qiang, Berge, Eivind, Robinson, Thompson G, Lindley, Richard, et al. Utility-Weighted Modified Rankin Scale Scores for the Assessment of Stroke Outcome: Pooled Analysis of 20 000+ Patients. Stroke 2020;51(8):2411-17. | https://dx.doi.org/10.1161/STROKEAHA.119.028523 |
| [Wang et al. (2020b)](https://dx.doi.org/10.18632/aging.103288) | Wang, Chung-Yuan, Miyoshi, Seido, Chen, Chang-Hung, Lee, Kai-Chun, Chang, Long-Chung, Chung, Jo-Hsuan. Walking ability and functional status after post-acute care for stroke rehabilitation in different age groups: a prospective study based on propensity score matching. Aging 2020;12(11):10704-14. | https://dx.doi.org/10.18632/aging.103288 |
| [Ware et al. (2020)](https://dx.doi.org/10.2196/16538) | Ware, Patrick, Ross, Heather J, Cafazzo, Joseph A, Boodoo, Chris, Munnery, Mikayla. Outcomes of a Heart Failure Telemonitoring Program Implemented as the Standard of Care in an Outpatient Heart Function Clinic: Pretest-Posttest Pragmatic Study. Journal of medical Internet research 2020;22(2):e16538. | https://dx.doi.org/10.2196/16538 |
| [Warraich et al. (2018)](https://dx.doi.org/10.1161/CIRCOUTCOMES.117.004528) | Warraich, Haider J, Kaltenbach, Lisa A, Fonarow, Gregg C, Peterson, Eric D. Adverse Change in Employment Status After Acute Myocardial Infarction: Analysis From the TRANSLATE-ACS Study. Circulation. Cardiovascular quality and outcomes 2018;11(6):e004528. | https://dx.doi.org/10.1161/CIRCOUTCOMES.117.004528 |
| [Watanabe-Fujinuma et al. (2020)](https://dx.doi.org/10.1186/s12955-020-01483-0) | Watanabe-Fujinuma, Emi, Origasa, Hideki, Bamber, Luke, Roessig, Lothar, Toyoda, Tetsumi, Haga, Yuri, Gwaltney, Chad. Psychometric properties of the Japanese version of the Kansas City Cardiomyopathy Questionnaire in Japanese patients with chronic heart failure. Health and quality of life outcomes 2020;18(1):236. | https://dx.doi.org/10.1186/s12955-020-01483-0 |
| [Werdan et al. (2016)](https://dx.doi.org/10.1159/000439584) | Werdan, Karl, Ebelt, Henning, Nuding, Sebastian, Hopfner, Florian, Stockl, Georg, Muller-Werdan, Ursula. Ivabradine in Combination with Metoprolol Improves Symptoms and Quality of Life in Patients with Stable Angina Pectoris: A post hoc Analysis from the ADDITIONS Trial. Cardiology 2016;133(2):83-90. | https://dx.doi.org/10.1159/000439584 |
| [Whynes (2013)](https://dx.doi.org/10.1186/1477-7525-11-155) | Whynes, David K. Does the correspondence between EQ-5D health state description and VAS score vary by medical condition?. Health and quality of life outcomes 2013;11():155. | https://dx.doi.org/10.1186/1477-7525-11-155 |
| [Whynes et al. (2013)](https://dx.doi.org/10.1177/0272989X12465016) | Whynes, David K, Sprigg, Nikola, Selby, James, Berge, Eivind, Bath, Philip M. Testing for differential item functioning within the EQ-5D. Medical decision making : an international journal of the Society for Medical Decision Making 2013;33(2):252-60. | https://dx.doi.org/10.1177/0272989X12465016 |
| [Winter et al. (2018)](https://dx.doi.org/10.1016/j.yebeh.2017.12.037) | Winter, Yaroslav, Daneshkhah, Naeimeh, Galland, Nikolaus, Kotulla, Isabel, Kruger, Anna. Health-related quality of life in patients with poststroke epilepsy. Epilepsy & behavior : E&B 2018;80():303-06. | https://dx.doi.org/10.1016/j.yebeh.2017.12.037 |
| [Wong et al. (2018)](https://dx.doi.org/10.1007/s11136-018-1887-3) | Wong, Carlos K H, Mulhern, Brendan, Cheng, Garvin H L. SF-6D population norms for the Hong Kong Chinese general population. Quality of life research : an international journal of quality of life aspects of treatment, care and rehabilitation 2018;27(9):2349-59. | https://dx.doi.org/10.1007/s11136-018-1887-3 |
| [Wu et al. (2013)](https://dx.doi.org/10.1177/1474515112468067) | Wu, Eline & Martensson, Jan. Enhanced external counterpulsation in patients with refractory angina pectoris: a pilot study with six months follow-up regarding physical capacity and health-related quality of life. European journal of cardiovascular nursing : journal of the Working Group on Cardiovascular Nursing of the European Society of Cardiology 2013;12(5):437-45. | https://dx.doi.org/10.1177/1474515112468067 |
| [Wu et al. (2014a)](https://dx.doi.org/10.1186/s12955-014-0156-6) | Wu, Jing, Han, Yuerong, Zhao, Fei-Li, Zhou, Jin, Chen, Zhijun. Validation and comparison of EuroQoL-5 dimension (EQ-5D) and Short Form-6 dimension (SF-6D) among stable angina patients. Health and quality of life outcomes 2014;12():156. | https://dx.doi.org/10.1186/s12955-014-0156-6 |
| [Wu et al. (2014b)](https://dx.doi.org/10.1186/s12955-014-0156-6) | Wu, Jing, Han, Yuerong, Xu, Judy, Lu, Yang, Cong, Hongliang, Zheng, Junyi. Chronic stable angina is associated with lower health-related quality of life: evidence from Chinese patients. PloS one 2014;9(5):e97294. | https://dx.doi.org/10.1371/journal.pone.0097294 |
| [Wu et al. (2014c)](https://dx.doi.org/10.1186/s12955-014-0156-6) | Wu, Xiaoning, Min, Lianqiu, Cong, Lin, Jia, Yujie, Liu, Chang, Zhao, Haiping, Liu, Ping. Sex differences in health-related quality of life among adult stroke patients in Northeastern China. Journal of clinical neuroscience : official journal of the Neurosurgical Society of Australasia 2014;21(6):957-61. | https://dx.doi.org/10.1016/j.jocn.2013.08.030 |
| [Wu et al. (2015a)](https://dx.doi.org/10.1159/000370027) | Wu, Monica, Villano, Angelo, Russo, Giulio, Di Franco, Antonino, Stazi, Alessandra, Lauria, Christian, et al. Poor tolerance and limited effects of isosorbide-5-mononitrate in microvascular angina. Cardiology 2015;130(4):201-06. | https://dx.doi.org/10.1159/000370027 |
| [Wu et al. (2015b)](https://dx.doi.org/10.1159/000370027) | Wu, Ming-Hsiu, Lee, Sheuan, Su, Hui-Yi. The effect of cognitive appraisal in middle-aged women stroke survivors and the psychological health of their caregivers: a follow-up study. Journal of clinical nursing 2015;24(21-22):3155-64. | https://dx.doi.org/10.1111/jocn.12926 |
| [Xian et al. (2016)](https://dx.doi.org/10.1016/j.jep.2016.03.066) | Xian, Shaoxiang, Yang, Zhongqi, Lee, Jun, Jiang, Zhiping, Ye, Xiaohan, Luo, Luyi, et al. A randomized, double-blind, multicenter, placebo-controlled clinical study on the efficacy and safety of Shenmai injection in patients with chronic heart failure. Journal of ethnopharmacology 2016;186():136-42. | https://dx.doi.org/10.1016/j.jep.2016.03.066 |
| [Xu et al. (2015)](https://dx.doi.org/10.1161/CIRCULATIONAHA.114.012826) | Xu, Xiao, Bao, Haikun, Strait, Kelly, Spertus, John A, Lichtman, Judith H, D'Onofrio, Gail, et al. Sex differences in perceived stress and early recovery in young and middle-aged patients with acute myocardial infarction. Circulation 2015;131(7):614-23. | https://dx.doi.org/10.1161/CIRCULATIONAHA.114.012826 |
| [Yang et al. (2017)](https://dx.doi.org/10.5535/arm.2017.41.5.761) | Yang, You-Na, Kim, Bo-Ram, Uhm, Kyeong Eun, Kim, Soo Jin, Lee, Seunghwan, Oh-Park, Mooyeon. Life Space Assessment in Stroke Patients. Annals of rehabilitation medicine 2017;41(5):761-68. | https://dx.doi.org/10.5535/arm.2017.41.5.761 |
| [Yeoh et al. (2018)](https://dx.doi.org/10.1186/s12955-018-1043-3) | Yeoh, Yen Shing, Koh, Gerald Choon-Huat, Tan, Chuen Seng, Lee, Kim En, Tu, Tian Ming, Singh, Rajinder, et al. Can acute clinical outcomes predict health-related quality of life after stroke: a one-year prospective study of stroke survivors. Health and quality of life outcomes 2018;16(1):221. | https://dx.doi.org/10.1186/s12955-018-1043-3 |
| [Yeoh et al. (2019)](https://dx.doi.org/10.1371/journal.pone.0211493) | Yeoh, Yen Shing, Koh, Gerald Choon-Huat, Tan, Chuen Seng, Tu, Tian Ming, Singh, Rajinder, Chang, Hui Meng, et al. Health-related quality of life loss associated with first-time stroke. PloS one 2019;14(1):e0211493. | https://dx.doi.org/10.1371/journal.pone.0211493 |
| [Yfantopoulos & (2020)](https://dx.doi.org/10.1007/s10198-020-01167-y) | Yfantopoulos, John. Health-related quality of life and health utilities in insulin-treated type 2 diabetes: the impact of related comorbidities/complications. The European journal of health economics : HEPAC : health economics in prevention and care 2020;21(5):729-43. | https://dx.doi.org/10.1007/s10198-020-01167-y |
| [Yu et al. (2019)](https://dx.doi.org/10.1007/s40520-018-1099-2) | Yu, Tsung, Enkh-Amgalan, Nomin, Zorigt, Ganchimeg, Hsu, Yea-Jen, Chen, Hsin-Jen. Gender differences and burden of chronic conditions: impact on quality of life among the elderly in Taiwan. Aging clinical and experimental research 2019;31(11):1625-33. | https://dx.doi.org/10.1007/s40520-018-1099-2 |
| [Zanaboni et al. (2013)](https://dx.doi.org/10.2196/jmir.2587) | Zanaboni, Paolo, Landolina, Maurizio, Marzegalli, Maurizio, Lunati, Maurizio, Perego, Giovanni B, Guenzati, Giuseppe, et al. Cost-utility analysis of the EVOLVO study on remote monitoring for heart failure patients with implantable defibrillators: randomized controlled trial. Journal of medical Internet research 2013;15(5):e106. | https://dx.doi.org/10.2196/jmir.2587 |
| [Zannad et al. (2015)](https://dx.doi.org/10.1093/eurheartj/ehu345) | Zannad, Faiez, De Ferrari, Gaetano M, Tuinenburg, Anton E, Wright, David, Brugada, Josep, Butter, Christian, et al. Chronic vagal stimulation for the treatment of low ejection fraction heart failure: results of the NEural Cardiac TherApy foR Heart Failure (NECTAR-HF) randomized controlled trial. European heart journal 2015;36(7):425-33. | https://dx.doi.org/10.1093/eurheartj/ehu345 |
| [Zarifis et al. (2015)](https://dx.doi.org/10.1002/clc.22479) | Zarifis, John, Grammatikou, Violetta, Kallistratos, Manolis, Katsivas, Apostolos. Treatment of Stable Angina Pectoris With Ivabradine in Everyday Practice: A Pan-Hellenic, Prospective, Noninterventional Study. Clinical cardiology 2015;38(12):725-32. | https://dx.doi.org/10.1002/clc.22479 |
| [Zeuner et al. (2017)](https://dx.doi.org/10.1080/02699052.2016.1218545) | Zeuner, Kirsten E, Knutzen, Arne, Kuhl, Carina, Moller, Bettina, Hellriegel, Helge, Margraf, Nils G, Deuschl, Gunther. Functional impact of different muscle localization techniques for Botulinum neurotoxin A injections in clinical routine management of post-stroke spasticity. Brain injury 2017;31(1):75-82. | https://dx.doi.org/10.1080/02699052.2016.1218545 |
| [Zhang et al. (2018)](http://dx.doi.org/10.14704/nq.2018.16.5.1327) | Zhang, Ying, Qin, Li, Shi, Yuanyuan. Effects of high-quality nursing services on the neurological functions and abilities of daily living of stroke patients. NeuroQuantology 2018;16(5):7-12. | http://dx.doi.org/10.14704/nq.2018.16.5.1327 |
| [Zhang et al. (2020)](https://dx.doi.org/10.1007/s13300-020-00788-z) | Zhang, Yichen, Wu, Jing, Chen, Yingyao. EQ-5D-3L Decrements by Diabetes Complications and Comorbidities in China. Diabetes therapy : research, treatment and education of diabetes and related disorders 2020;11(4):939-50. | https://dx.doi.org/10.1007/s13300-020-00788-z |
| [Zhang et al. (2021)](http://dx.doi.org/10.1111/liv.14780) | Zhang, Meng, Zhang, Hui-Wen, Jin, Jing-Lu, Liu, Hui-Hui, Zhang, Yan, Guo, Yuan-Lin, et al. Impact of liver fibrosis score on prognosis in patients with previous myocardial infarction: A prospective cohort study. Liver International 2021;():n. pag.. | http://dx.doi.org/10.1111/liv.14780 |
| [Zhen et al. (2016)](https://dx.doi.org/10.3389/fneur.2016.00235) | Zhen, Xiaoyue, Zheng, Yu, Hong, Xunning, Chen, Yan, Gu, Ping, Tang, Jinrong, et al. Physiological Ischemic Training Promotes Brain Collateral Formation and Improves Functions in Patients with Acute Cerebral Infarction. Frontiers in neurology 2016;7():235. | https://dx.doi.org/10.3389/fneur.2016.00235 |
| [Zhou et al. (2019)](http://dx.doi.org/10.1161/STROKEAHA.118.021558) | Zhou, Bo, Zhao, Qiongrui, Shi, Jingpu, Zhang, Jing, Luo, Rong, Zhao, Yi, et al. Caregiver-Delivered Stroke Rehabilitation in Rural China: The RECOVER Randomized Controlled Trial. Stroke 2019;50(7):1825-30. | http://dx.doi.org/10.1161/STROKEAHA.118.021558 |
| [Zugck et al. (2014)](https://dx.doi.org/10.1007/s12325-014-0147-3) | Zugck, Christian & Martinka, Peter. Ivabradine treatment in a chronic heart failure patient cohort: symptom reduction and improvement in quality of life in clinical practice. Advances in therapy 2014;31(9):961-74. | https://dx.doi.org/10.1007/s12325-014-0147-3 |

Appendix 5- Summary details of all identified studies reporting cardiovascular health state utility values

| **Study** | **Number of study participants** | **Country** | **Instrument** | **Stable Angina** | **Unstable Angina** | **MI** | **Post-MI** | **Stroke** | **Post-stroke** | **TIA** | **Post-TIA** | **Heart failure** | **PAD** | **Number of states** | **Comorbid T2DM** | **Comorbid CKD** |
| --- | --- | --- | --- | --- | --- | --- | --- | --- | --- | --- | --- | --- | --- | --- | --- | --- |
| [Marrett et al. (2013)](https://dx.doi.org/10.1186/1477-7525-11-175) | 743 | OECD (Across Europe inc. UK) | Other generic instrument |  |  |  |  |  |  |  |  |  | ✓ | 1 |  |  |
| [Rieckmann et al. (2020)](https://dx.doi.org/10.1186/s12955-020-01312-4) | 1263 | OECD (Across Europe) | EQ5D-3L | ✓ |  |  |  |  |  |  |  |  |  | 1 |  |  |
| [Hornslien et al. (2013)](https://dx.doi.org/10.1161/STROKEAHA.113.001022) | 882 | OECD (Across North Europe) | EQ5D-3L |  |  |  |  | ✓ |  |  |  |  |  | 1 |  |  |
| [Cadilhac et al. (2017)](https://dx.doi.org/10.1161/STROKEAHA.116.015714) | 9787 | OECD (Austrailia) | EQ5D-3L |  |  |  |  | ✓ |  |  |  |  |  | 1 |  |  |
| [Adey-Wakeling et al. (2016)](https://dx.doi.org/10.1097/PHM.0000000000000496) | 263 | OECD (Australia) | EQ5D-3L |  |  |  |  |  | ✓ |  |  |  |  | 1 |  |  |
| [Ahimastos et al. (2013)](https://dx.doi.org/10.1001/jama.2012.216237) | 106 | OECD (Australia) | SF-36 |  |  |  |  |  |  |  |  |  | ✓ | 1 |  |  |
| [Andrew et al. (2016)](https://dx.doi.org/10.1007/s11136-016-1234-5) | 173 | OECD (Australia) | EQ-VAS |  |  |  |  | ✓ |  |  |  |  |  | 1 |  |  |
| [Hutchinson et al. (2015)](https://dx.doi.org/10.1186/s12955-015-0260-2) | 151 | OECD (Australia) | Other generic instrument |  |  |  |  |  |  |  |  |  | ✓ | 1 |  |  |
| [Hwang et al. (2018)](http://dx.doi.org/10.1016/j.hlc.2018.11.010) | 53 | OECD (Australia) | EQ5D-3L |  |  |  |  |  |  |  |  | ✓ |  | 1 |  |  |
| [Kularatna et al. (2020)](https://dx.doi.org/10.1186/s12955-020-01368-2) | 141 | OECD (Australia) | EQ5D-5L |  |  |  |  |  |  |  |  | ✓ |  | 1 |  |  |
| [Lynch et al. (2020)](https://dx.doi.org/10.1080/09638288.2020.1852616) | 5252 | OECD (Australia) | EQ-VAS |  |  |  |  | ✓ | ✓ |  |  |  |  | 2 |  |  |
| [Nandal et al. (2021)](http://dx.doi.org/10.1111/imj.14749) | 65 | OECD (Australia) | EQ-VAS |  |  |  |  |  |  |  |  | ✓ |  | 1 |  |  |
| [Phan et al. (2021)](https://dx.doi.org/10.1111/ene.14531) | 6852 | OECD (Australia) | EQ5D-3L |  |  |  |  | ✓ |  |  |  |  |  | 1 |  |  |
| [Prichard et al. (2021)](https://dx.doi.org/10.1007/s11136-020-02722-z) | 75 | OECD (Australia) | EQ5D-5L |  |  |  |  |  |  |  |  | ✓ |  | 1 |  |  |
| [Scuffham et al. (2017)](http://dx.doi.org/10.1016/j.ijcard.2016.11.030) | 280 | OECD (Australia) | EQ5D-3L |  |  |  |  |  |  |  |  | ✓ |  | 1 |  |  |
| [Stolz et al. (2019)](https://dx.doi.org/10.1097/MRR.0000000000000375) | 36 | OECD (Australia) | EQ-VAS |  |  |  |  | ✓ |  |  |  |  |  | 1 |  |  |
| [Thrift et al. (2019)](http://dx.doi.org/10.1016/j.jstrokecerebrovasdis.2019.01.026) | 431 | OECD (Australia) | EQ5D-3L |  |  |  |  | ✓ | ✓ |  |  |  |  | 2 |  |  |
| [Turkstra et al. (2013)](https://dx.doi.org/10.1186/1471-2261-13-33) | 430 | OECD (Australia) | SF-36 |  |  | ✓ | ✓ |  |  |  |  |  |  | 2 |  |  |
| [Varnfield et al. (2014)](https://dx.doi.org/10.1136/heartjnl-2014-305783) | 41 | OECD (Australia) | EQ5D-3L |  |  | ✓ | ✓ |  |  |  |  |  |  | 2 |  |  |
| [Toell et al. (2020)](http://dx.doi.org/10.1016/j.eclinm.2020.100476) | 2149 | OECD (Austria) | EQ5D-3L |  |  |  |  | ✓ | ✓ | ✓ | ✓ |  |  | 4 |  |  |
| [Visser et al. (2015)](https://dx.doi.org/10.1016/j.apmr.2015.04.007) | 166 | OECD (Belgium and Netherlands) | EQ5D-5L |  |  |  |  | ✓ |  |  |  |  |  | 1 |  |  |
| [Dewilde et al. (2019)](https://dx.doi.org/10.1186/s12955-018-1069-6) | 539 | OECD (Belgium) | EQ5D-3L |  |  |  |  | ✓ |  |  |  |  |  | 1 |  |  |
| [Appau et al. (2019)](https://dx.doi.org/10.1177/0269215519834064) | 488 | OECD (Canada) | EQ5D-3L |  |  |  |  | ✓ |  |  |  |  |  | 1 |  |  |
| [Barclay & (2014)](https://dx.doi.org/10.1016/j.jclinepi.2013.12.003) | 168 | OECD (Canada) | SF-36 |  |  |  |  |  | ✓ |  |  |  |  | 1 |  |  |
| [Cohen et al. (2018)](https://dx.doi.org/10.1016/j.apmr.2017.12.007) | 75 | OECD (Canada) | SF-36 |  |  |  |  | ✓ |  |  |  |  |  | 1 |  |  |
| [Cui et al. (2013)](http://ovidsp.ovid.com/ovidweb.cgi?T=JS&PAGE=reference&D=med10&NEWS=N&AN=24359716) | 179 | OECD (Canada) | SF-36 |  |  |  |  |  |  |  |  | ✓ |  | 1 |  |  |
| [Davies et al. (2015)](https://dx.doi.org/10.1186/s12955-015-0266-9) | 199 | OECD (Canada) | Directly elicited (TTO) | ✓ | ✓ | ✓ |  | ✓ |  |  |  | ✓ | ✓ | 6 |  | ✓ |
| [Joundi et al. (2021)](https://dx.doi.org/10.1161/STROKEAHA.120.033872) | 165 | OECD (Canada) | EQ5D-3L |  |  |  |  | ✓ | ✓ |  |  |  |  | 2 |  |  |
| [Klassen et al. (2020)](https://dx.doi.org/10.1161/STROKEAHA.120.029245) | 25 | OECD (Canada) | EQ5D-5L |  |  |  |  | ✓ |  |  |  |  |  | 1 |  |  |
| [Mayo et al. (2015a)](https://dx.doi.org/10.1007/s11136-013-0605-4) | 678 | OECD (Canada) | EQ-VAS |  |  |  |  | ✓ |  |  |  |  |  | 1 |  |  |
| [Mayo et al. (2015b)](https://dx.doi.org/10.1177/0269215514565396) | 93 | OECD (Canada) | EQ5D-3L |  |  |  |  | ✓ |  |  |  |  |  | 1 |  |  |
| [Muggah et al. (2013)](https://dx.doi.org/10.1186/1471-2458-13-16) | 698 | OECD (Canada) | HUI2 |  |  | ✓ |  | ✓ |  |  |  |  |  | 2 |  |  |
| [Van et al. (2019)](https://dx.doi.org/10.1001/jama.2019.0710) | 380 | OECD (Canada) | EQ5D-5L |  |  |  |  |  |  |  |  | ✓ |  | 1 |  |  |
| [Ware et al. (2020)](https://dx.doi.org/10.2196/16538) | 208 | OECD (Canada) | EQ5D-5L |  |  |  |  |  |  |  |  | ✓ |  | 1 |  |  |
| [Aguirre-Acevedo et al. (2020)](http://dx.doi.org/10.1016/j.rccar.2019.04.003) | 544 | OECD (Colombia) | SF-36 |  |  |  |  |  |  |  |  | ✓ |  | 1 |  |  |
| [Kielbergerova et al. (2015)](https://dx.doi.org/10.1007/s12975-015-0418-6) | 341 | OECD (Czech Republic) | SF-36 |  |  |  |  | ✓ |  |  |  |  |  | 1 |  |  |
| [Berg et al. (2017)](https://dx.doi.org/10.1016/j.jpsychores.2017.01.003) | 987 | OECD (Denmark) | EQ5D-3L |  |  |  |  |  |  |  |  | ✓ |  | 1 |  |  |
| [Rasmussen et al. (2019)](https://dx.doi.org/10.1097/JCN.0000000000000583) | 14115 | OECD (Denmark) | EQ5D-3L | ✓ | ✓ | ✓ |  |  |  |  |  |  |  | 3 |  |  |
| [Rasmussen et al. (2020a)](https://dx.doi.org/10.1177/1474515120902390) | 1506 | OECD (Denmark) | EQ5D-5L |  |  |  |  |  |  |  |  | ✓ |  | 1 |  |  |
| [Rasmussen et al. (2020b)](http://dx.doi.org/10.1093/ehjcvp/pvaa097) | 1464 | OECD (Denmark) | EQ5D-5L |  |  |  |  |  |  |  |  | ✓ |  | 1 |  |  |
| [Rasmussen et al. (2020c)](http://dx.doi.org/10.1093/ehjqcco/qcaa026) | 1499 | OECD (Denmark) | EQ5D-5L |  |  |  |  |  |  |  |  | ✓ |  | 1 |  |  |
| [Schneider et al. (2021)](https://dx.doi.org/10.1016/j.jstrokecerebrovasdis.2020.105499) | 352 | OECD (Estonia) | EQ5D-3L |  |  |  |  |  | ✓ |  |  |  |  | 1 |  |  |
| [Harno et al. (2014)](https://dx.doi.org/10.1212/WNL.0000000000000818) | 824 | OECD (Finland) | EQ-VAS |  |  |  |  |  | ✓ |  |  |  |  | 1 |  |  |
| [Puumalainen et al. (2016)](https://dx.doi.org/10.1111/ane.12509) | 823 | OECD (Finland) | Other generic instrument |  |  |  |  | ✓ | ✓ |  |  |  |  | 2 |  |  |
| [Raggi et al. (2016)](https://dx.doi.org/10.1371/journal.pone.0159293) | 5639 | OECD (Finland, Poland and Spain) | Other generic instrument | ✓ |  |  |  |  |  |  |  |  |  | 1 |  |  |
| [Pavy et al. (2015)](https://dx.doi.org/10.1016/j.acvd.2014.09.006) | 323 | OECD (France) | SF-36 | ✓ | ✓ | ✓ |  |  |  |  |  | ✓ |  | 4 |  |  |
| [Prevost et al. (2015)](https://dx.doi.org/10.1177/2047487313512217) | 46 | OECD (France) | SF-36 |  |  |  |  |  |  |  |  |  | ✓ | 1 |  |  |
| [Schulte et al. (2019)](https://dx.doi.org/10.1024/0301-1526/a000798) | 1781 | OECD (Germany and Austria) | EQ-VAS |  |  |  |  |  |  |  |  |  | ✓ | 1 |  |  |
| [Chavanon et al. (2017)](https://dx.doi.org/10.1007/s00392-017-1101-6) | 530 | OECD (Germany and Serbia) | SF-36 |  |  |  |  |  |  |  |  | ✓ |  | 1 |  |  |
| [Boczor et al. (2019)](http://dx.doi.org/10.1186/s12889-019-7623-2) | 3155 | OECD (Germany) | EQ5D-5L |  |  |  |  |  |  |  |  | ✓ |  | 1 |  |  |
| [Diekmann et al. (2019)](https://dx.doi.org/10.1080/03007995.2019.1646000) | 198 | OECD (Germany) | EQ5D-3L |  |  |  |  | ✓ | ✓ |  |  |  |  | 2 |  |  |
| [Edelmann et al. (2015)](https://dx.doi.org/10.1002/ejhf.203) | 415 | OECD (Germany) | SF-36 |  |  |  |  |  |  |  |  | ✓ |  | 1 |  |  |
| [Graessel et al. (2014)](https://dx.doi.org/10.1097/MRR.0000000000000060) | 204 | OECD (Germany) | EQ5D-3L |  |  |  |  | ✓ |  |  |  |  |  | 1 |  |  |
| [Guder et al. (2015)](https://dx.doi.org/10.1002/ejhf.252) | 363 | OECD (Germany) | SF-36 |  |  |  |  |  |  |  |  | ✓ |  | 1 |  |  |
| [Hotter et al. (2018)](https://dx.doi.org/10.1177/2396987318771174) | 57 | OECD (Germany) | EQ5D-3L |  |  |  |  | ✓ | ✓ |  |  |  |  | 2 |  |  |
| [Khattab et al. (2013)](https://dx.doi.org/10.1177/2047487312447751) | 207 | OECD (Germany) | SF-36 | ✓ |  |  |  |  |  |  |  |  |  | 1 |  |  |
| [Krack et al. (2018)](https://dx.doi.org/10.1186/s12877-018-0827-y) | 127 | OECD (Germany) | EQ-VAS |  |  | ✓ |  |  |  |  |  |  |  | 1 |  |  |
| [Laxy et al. (2015)](https://dx.doi.org/10.1016/j.jval.2015.07.003) | 901 | OECD (Germany) | EQ5D-3L |  |  | ✓ |  |  |  |  |  |  |  | 1 | ✓ |  |
| [Laxy et al. (2021)](https://dx.doi.org/10.1016/j.jval.2020.09.017) | 1105 | OECD (Germany) | EQ5D-5L |  |  | ✓ |  | ✓ |  |  |  | ✓ |  | 3 |  |  |
| [Meisinger et al. (2019)](http://dx.doi.org/10.1007/s11136-019-02306-6) | 270 | OECD (Germany) | SF-36 |  |  | ✓ | ✓ |  |  |  |  |  |  | 2 |  |  |
| [Muller-Werdan et al. (2014)](http://ovidsp.ovid.com/ovidweb.cgi?T=JS&PAGE=reference&D=med11&NEWS=N&AN=25193811) | 479 | OECD (Germany) | EQ5D-3L | ✓ |  |  |  |  |  |  |  |  |  | 1 |  |  |
| [Nolte et al. (2015)](https://dx.doi.org/10.1177/2047487314526071) | 20 | OECD (Germany) | SF-36 |  |  |  |  |  |  |  |  | ✓ |  | 1 |  |  |
| [Peters-Klimm et al. (2013)](https://dx.doi.org/10.1177/1474515112439964) | 318 | OECD (Germany) | SF-36 |  |  |  |  |  |  |  |  | ✓ |  | 1 |  |  |
| [Pisa et al. (2015)](http://dx.doi.org/10.2147/PPA.S88167) | 300 | OECD (Germany) | EQ5D-3L |  |  |  |  |  |  |  |  | ✓ |  | 1 |  |  |
| [Robles-Zurita et al. (2020)](https://dx.doi.org/10.1007/s10198-020-01235-3) | 322 | OECD (Germany) | EQ5D-3L |  |  | ✓ | ✓ |  |  |  |  | ✓ |  | 3 | ✓ |  |
| [Sadlonova et al. (2021)](https://dx.doi.org/10.1016/j.jpsychores.2020.110353) | 364 | OECD (Germany) | EQ5D-3L |  |  |  |  | ✓ | ✓ |  |  |  |  | 2 |  |  |
| [Scherer et al. (2013)](https://dx.doi.org/10.1016/j.ejim.2013.01.003) | 589 | OECD (Germany) | SF-36 |  |  |  |  |  |  |  |  | ✓ |  | 1 |  |  |
| [Schowalter et al. (2013)](https://dx.doi.org/10.1007/s00392-012-0531-4) | 699 | OECD (Germany) | SF-36 |  |  |  |  |  |  |  |  | ✓ |  | 1 |  |  |
| [Seidel et al. (2014)](https://dx.doi.org/10.1371/journal.pone.0091176) | 119 | OECD (Germany) | SF-36 |  | ✓ | ✓ |  | ✓ |  | ✓ |  | ✓ | ✓ | 6 |  | ✓ |
| [Seidl et al. (2015)](https://dx.doi.org/10.1007/s10198-014-0623-3) | 329 | OECD (Germany) | EQ5D-3L |  |  | ✓ |  |  |  |  |  |  |  | 1 |  |  |
| [Seidl et al. (2017)](https://dx.doi.org/10.1016/j.jval.2016.10.001) | 138 | OECD (Germany) | EQ5D-3L |  |  | ✓ | ✓ |  |  |  |  |  |  | 2 |  |  |
| [Werdan et al. (2016)](https://dx.doi.org/10.1159/000439584) | 877 | OECD (Germany) | EQ5D-3L | ✓ |  |  |  |  |  |  |  |  |  | 1 |  |  |
| [Winter et al. (2018)](https://dx.doi.org/10.1016/j.yebeh.2017.12.037) | 351 | OECD (Germany) | EQ5D-3L |  |  |  |  |  | ✓ |  |  |  |  | 1 |  |  |
| [Zeuner et al. (2017)](https://dx.doi.org/10.1080/02699052.2016.1218545) | 14 | OECD (Germany) | EQ5D-3L |  |  |  |  |  | ✓ |  |  |  |  | 1 |  |  |
| [Zugck et al. (2014)](https://dx.doi.org/10.1007/s12325-014-0147-3) | 1956 | OECD (Germany) | EQ5D-3L |  |  |  |  |  |  |  |  | ✓ |  | 1 |  |  |
| [Pocock et al. (2021)](https://dx.doi.org/10.1136/openhrt-2020-001499) | 9126 | OECD (Global) | EQ5D-3L |  |  | ✓ | ✓ |  |  |  |  |  |  | 2 |  |  |
| [Yfantopoulos & (2020)](https://dx.doi.org/10.1007/s10198-020-01167-y) | 354 | OECD (Greece) | EQ5D-5L |  |  | ✓ |  | ✓ |  |  |  | ✓ | ✓ | 4 | ✓ |  |
| [Zarifis et al. (2015)](https://dx.doi.org/10.1002/clc.22479) | 2403 | OECD (Greece) | EQ5D-3L | ✓ |  |  |  |  |  |  |  |  |  | 1 |  |  |
| [Farkas et al. (2020)](https://dx.doi.org/10.1024/0301-1526/a000845) | 765 | OECD (Hungary) | EQ5D-3L |  |  |  |  |  |  |  |  |  | ✓ | 1 |  |  |
| [Szocs et al. (2020)](https://dx.doi.org/10.1371/journal.pone.0241059) | 200 | OECD (Hungary) | EQ5D-5L |  |  |  |  | ✓ |  |  |  |  |  | 1 |  |  |
| [Ketilsdottir et al. (2019)](https://dx.doi.org/10.1002/ehf2.12369) | 124 | OECD (Iceland) | EQ-VAS |  |  |  |  |  |  |  |  | ✓ |  | 1 |  |  |
| [Sprigg et al. (2013)](https://dx.doi.org/10.1161/STROKEAHA.113.002201) | 2238 | OECD (International inc. UK) | EQ-VAS |  |  |  |  | ✓ |  |  |  |  |  | 1 |  |  |
| [Ambrosy et al. (2017)](https://dx.doi.org/10.1016/j.ahj.2016.12.017) | 1078 | OECD (International) | EQ5D-3L |  |  |  |  |  |  |  |  | ✓ |  | 1 |  |  |
| [Hamo et al. (2015)](https://dx.doi.org/10.1161/CIRCHEARTFAILURE.114.001838) | 1431 | OECD (International) | EQ5D-3L |  |  |  |  |  |  |  |  | ✓ |  | 1 |  |  |
| [Li et al. (2013)](https://dx.doi.org/10.1016/j.cardfail.2013.03.008) | 2288 | OECD (International) | EQ5D-3L |  |  |  |  |  |  |  |  | ✓ |  | 1 |  |  |
| [Magnuson et al. (2017)](https://dx.doi.org/10.1016/j.jacc.2017.05.063) | 7067 | OECD (International) | EQ5D-3L |  |  |  | ✓ |  |  |  |  |  |  | 1 |  |  |
| [Nicolau et al. (2020)](https://dx.doi.org/10.1002/clc.23476) | 8968 | OECD (International) | EQ5D-3L |  |  |  | ✓ |  |  |  |  |  |  | 1 | ✓ |  |
| [Shireman et al. (2017)](https://dx.doi.org/10.1161/STROKEAHA.116.014735) | 196 | OECD (International) | EQ5D-3L |  |  |  |  | ✓ | ✓ |  |  |  |  | 2 |  |  |
| [Smolderen et al. (2015)](https://dx.doi.org/10.1161/JAHA.114.001424) | 3572 | OECD (International) | EQ5D-3L |  |  | ✓ |  |  |  |  |  |  |  | 1 |  |  |
| [Stone et al. (2016)](https://dx.doi.org/10.1093/eurheartj/ehv436) | 33 | OECD (International) | EQ5D-3L |  |  | ✓ | ✓ |  |  |  |  |  |  | 2 |  |  |
| [Wang et al. (2020a)](https://dx.doi.org/10.1161/STROKEAHA.119.028523) | 22946 | OECD (International) | EQ5D-3L |  |  |  |  | ✓ |  |  |  |  |  | 1 |  |  |
| [Guidon & (2013)](https://dx.doi.org/10.3109/09638288.2012.694963) | 29 | OECD (Ireland) | SF-36 |  |  |  |  |  |  |  |  |  | ✓ | 1 |  |  |
| [Dankner et al. (2016)](http://ovidsp.ovid.com/ovidweb.cgi?T=JS&PAGE=reference&D=med13&NEWS=N&AN=27548022) | 51 | OECD (Israel) | SF-36 |  |  |  |  |  | ✓ |  |  |  |  | 1 |  |  |
| [Aprile et al. (2015)](http://ovidsp.ovid.com/ovidweb.cgi?T=JS&PAGE=reference&D=med12&NEWS=N&AN=25739508) | 106 | OECD (Italy) | SF-36 |  |  |  |  | ✓ |  |  |  |  |  | 1 |  |  |
| [Campo et al. (2020)](https://dx.doi.org/10.1136/heartjnl-2019-316349) | 117 | OECD (Italy) | EQ-VAS |  |  | ✓ | ✓ |  |  |  |  |  |  | 2 |  |  |
| [D'Aniello et al. (2014)](https://dx.doi.org/10.1016/j.jns.2014.01.005) | 81 | OECD (Italy) | SF-36 |  |  |  |  |  | ✓ |  |  |  |  | 1 |  |  |
| [De et al. (2018)](https://dx.doi.org/10.1371/journal.pone.0199770) | 1308 | OECD (Italy) | EQ5D-5L | ✓ | ✓ |  |  |  |  |  |  |  |  | 2 |  |  |
| [Fattirolli et al. (2015)](https://dx.doi.org/10.1007/s11739-015-1203-y) | 298 | OECD (Italy) | SF-36 | ✓ | ✓ | ✓ |  |  |  |  |  | ✓ |  | 4 |  |  |
| [Lamberti et al. (2017)](https://dx.doi.org/10.23736/S1973-9087.16.04322-7) | 17 | OECD (Italy) | SF-36 |  |  |  |  | ✓ |  |  |  |  |  | 1 |  |  |
| [Lombardi et al. (2015)](https://dx.doi.org/10.1016/j.nut.2014.04.021) | 25 | OECD (Italy) | EQ5D-3L |  |  |  |  |  |  |  |  | ✓ |  | 1 |  |  |
| [Masciocco et al. (2013)](http://dx.doi.org/10.1159/000355169) | 110 | OECD (Italy) | SF-36 |  |  |  |  |  |  |  |  | ✓ |  | 1 |  |  |
| [Villano et al. (2013)](https://dx.doi.org/10.1016/j.amjcard.2013.02.045) | 46 | OECD (Italy) | EQ-VAS | ✓ |  |  |  |  |  |  |  |  |  | 1 |  |  |
| [Wu et al. (2015a)](https://dx.doi.org/10.1159/000370027) | 9 | OECD (Italy) | EQ5D-3L | ✓ |  |  |  |  |  |  |  |  |  | 1 |  |  |
| [Matsumoto et al. (2016)](http://ovidsp.ovid.com/ovidweb.cgi?T=JS&PAGE=reference&D=med13&NEWS=N&AN=27996326) | 552 | OECD (Japan) | SF-36 |  |  |  |  | ✓ |  |  |  |  |  | 1 |  |  |
| [Miyahara et al. (2018)](https://dx.doi.org/10.1097/HCR.0000000000000296) | 41 | OECD (Japan) | SF-36 |  |  |  |  |  |  |  |  | ✓ |  | 1 |  |  |
| [Nagayama et al. (2017)](https://dx.doi.org/10.1080/10749357.2017.1289686) | 48 | OECD (Japan) | SF-36 |  |  |  |  | ✓ |  |  |  |  |  | 1 |  |  |
| [Nozoe et al. (2021)](http://dx.doi.org/10.3390/ijerph18010251) | 50 | OECD (Japan) | EQ5D-3L |  |  |  |  | ✓ |  |  |  |  |  | 1 |  |  |
| [Sasaki et al. (2018)](https://dx.doi.org/10.1080/10749357.2018.1492775) | 22 | OECD (Japan) | EQ5D-3L |  |  |  |  | ✓ |  |  |  |  |  | 1 |  |  |
| [Sobajima et al. (2015)](https://dx.doi.org/10.1536/ihj.14-266) | 49 | OECD (Japan) | SF-36 |  |  |  |  |  |  |  |  | ✓ |  | 1 |  |  |
| [Wang et al. (2020b)](https://dx.doi.org/10.18632/aging.103288) | 316 | OECD (Japan) | EQ5D-3L |  |  |  |  | ✓ | ✓ |  |  |  |  | 2 |  |  |
| [Watanabe-Fujinuma et al. (2020)](https://dx.doi.org/10.1186/s12955-020-01483-0) | 141 | OECD (Japan) | EQ5D-3L |  |  |  |  |  |  |  |  | ✓ |  | 1 |  |  |
| [Chang et al. (2016)](https://dx.doi.org/10.1007/s00415-016-8119-y) | 2271 | OECD (Korea) | EQ5D-3L |  |  |  |  | ✓ |  |  |  |  |  | 1 |  |  |
| [Han et al. (2019)](https://dx.doi.org/10.1136/bmjopen-2018-028673) | 193 | OECD (Korea) | EQ5D-3L |  |  |  |  | ✓ |  |  |  |  |  | 1 |  |  |
| [Hong et al. (2018)](https://dx.doi.org/10.1007/s40261-018-0659-8) | 100 | OECD (Korea) | EQ5D-5L |  |  |  |  |  |  |  |  | ✓ |  | 1 |  |  |
| [Im et al. (2020)](https://dx.doi.org/10.1016/j.jstrokecerebrovasdis.2020.105406) | 181 | OECD (Korea) | EQ5D-3L |  |  |  |  | ✓ | ✓ |  |  |  |  | 2 |  |  |
| [Jeon et al. (2017a)](https://dx.doi.org/10.5535/arm.2017.41.4.556) | 46 | OECD (Korea) | EQ5D-3L |  |  |  |  | ✓ |  |  |  |  |  | 1 |  |  |
| [Jeon et al. (2017b)](https://dx.doi.org/10.5535/arm.2017.41.5.743) | 757 | OECD (Korea) | EQ-VAS |  |  |  |  |  | ✓ |  |  |  |  | 1 |  |  |
| [Kim & (2019)](https://dx.doi.org/10.1111/ggi.13797) | 504 | OECD (Korea) | EQ5D-3L |  |  |  |  | ✓ |  |  |  |  |  | 1 |  |  |
| [Kim et al. (2015)](https://dx.doi.org/10.1097/MRR.0000000000000103) | 22 | OECD (Korea) | SF-36 |  |  |  |  | ✓ |  |  |  |  |  | 1 |  |  |
| [Kim et al. (2016)](https://dx.doi.org/10.5535/arm.2016.40.6.1010) | 130 | OECD (Korea) | EQ5D-3L |  |  |  |  | ✓ |  |  |  |  |  | 1 |  |  |
| [Kim et al. (2020)](https://dx.doi.org/10.2196/15377) | 99 | OECD (Korea) | EQ5D-3L |  |  |  |  |  | ✓ |  |  |  |  | 1 |  |  |
| [Kweon et al. (2017)](https://dx.doi.org/10.5535/arm.2017.41.2.248) | 53 | OECD (Korea) | SF-36 |  | ✓ | ✓ |  |  |  |  |  | ✓ |  | 3 |  |  |
| [Kwon et al. (2018)](https://dx.doi.org/10.1371/journal.pone.0195713) | 575 | OECD (Korea) | EQ5D-3L |  |  |  |  | ✓ |  |  |  |  |  | 1 |  |  |
| [Lee et al. (2018)](https://dx.doi.org/10.1097/PHM.0000000000000920) | 32 | OECD (Korea) | EQ5D-3L |  |  |  |  | ✓ |  |  |  |  |  | 1 |  |  |
| [Lee et al. (2019)](https://dx.doi.org/10.1093/ehjci/jey099) | 903 | OECD (Korea) | EQ5D-3L |  | ✓ |  |  |  |  |  |  |  |  | 1 |  |  |
| [Min & (2015)](https://dx.doi.org/10.1093/ageing/afv060) | 6698 | OECD (Korea) | EQ5D-3L |  |  |  |  |  | ✓ |  |  |  |  | 1 |  |  |
| [Ock et al. (2016)](https://dx.doi.org/10.1186/s12889-016-2904-5) | 360 | OECD (Korea) | EQ5D-3L | ✓ | ✓ |  |  | ✓ |  |  |  | ✓ |  | 4 |  |  |
| [Pyo et al. (2017)](https://dx.doi.org/10.5535/arm.2017.41.6.935) | 18 | OECD (Korea) | SF-36 |  |  |  |  | ✓ |  |  |  |  |  | 1 |  |  |
| [Rha et al. (2018)](https://dx.doi.org/10.4070/kcj.2017.0340) | 1208 | OECD (Korea) | EQ5D-3L |  |  |  |  |  |  |  |  |  | ✓ | 1 |  |  |
| [Yang et al. (2017)](https://dx.doi.org/10.5535/arm.2017.41.5.761) | 34 | OECD (Korea) | EQ5D-3L |  |  |  |  | ✓ |  |  |  |  |  | 1 |  |  |
| [Krishnan et al. (2020)](https://dx.doi.org/10.1016/j.ekir.2020.09.028) | 58 | OECD (Multinational) | EQ5D-3L |  |  |  |  | ✓ | ✓ |  |  |  |  | 2 |  | ✓ |
| [Albuquerque de et al. (2020)](https://dx.doi.org/10.1186/s12955-020-01508-8) | 426 | OECD (Multiple across Europe) | EQ5D-3L |  |  |  |  |  |  |  |  | ✓ |  | 1 |  |  |
| [Visser et al. (2016)](https://dx.doi.org/10.1161/STROKEAHA.115.010961) | 78 | OECD (Netherlands and Belgium) | EQ5D-5L |  |  |  |  | ✓ | ✓ |  |  |  |  | 2 |  |  |
| [Arwert et al. (2016)](https://dx.doi.org/10.1016/j.apmr.2015.09.018) | 44 | OECD (Netherlands) | SF-36 |  |  |  |  | ✓ |  |  |  |  |  | 1 |  |  |
| [Arwert et al. (2017)](https://dx.doi.org/10.1007/s10926-016-9651-4) | 46 | OECD (Netherlands) | SF-36 |  |  |  |  | ✓ |  |  |  |  |  | 1 |  |  |
| [Benda et al. (2015)](https://dx.doi.org/10.1371/journal.pone.0141256) | 20 | OECD (Netherlands) | SF-36 |  |  |  |  |  |  |  |  | ✓ |  | 1 |  |  |
| [Bosma et al. (2014)](https://dx.doi.org/10.1177/0284185113496560) | 79 | OECD (Netherlands) | SF-36 |  |  |  |  |  |  |  |  |  | ✓ | 1 |  |  |
| [de et al. (2021)](https://dx.doi.org/10.23736/S1973-9087.21.06335-8) | 360 | OECD (Netherlands) | EQ5D-5L |  |  |  |  | ✓ |  |  |  |  |  | 1 |  |  |
| [Dijkland et al. (2018)](http://dx.doi.org/10.1161/STROKEAHA.117.020194) | 267 | OECD (Netherlands) | EQ5D-3L |  |  |  |  | ✓ |  |  |  |  |  | 1 |  |  |
| [Fakhry et al. (2015)](https://dx.doi.org/10.1001/jama.2015.14851) | 106 | OECD (Netherlands) | SF-36 |  |  |  |  |  |  |  |  |  | ✓ | 1 |  |  |
| [Fokkenrood et al. (2015)](https://dx.doi.org/10.1016/j.ejvs.2014.11.002) | 41 | OECD (Netherlands) | SF-36 |  |  |  |  |  |  |  |  |  | ✓ | 1 |  |  |
| [Gijsberts et al. (2015)](https://dx.doi.org/10.1136/openhrt-2014-000231) | 1421 | OECD (Netherlands) | EQ5D-3L | ✓ | ✓ | ✓ |  |  |  |  |  |  |  | 3 |  |  |
| [Gingele et al. (2019)](http://dx.doi.org/10.1007/s12471-019-01323-x) | 173 | OECD (Netherlands) | EQ5D-3L |  |  |  |  |  |  |  |  | ✓ |  | 1 |  |  |
| [Groeneveld et al. (2019a)](https://dx.doi.org/10.1016/j.jstrokecerebrovasdis.2018.10.033) | 239 | OECD (Netherlands) | EQ5D-3L |  |  |  |  | ✓ | ✓ |  |  |  |  | 2 |  |  |
| [Groeneveld et al. (2019b)](https://dx.doi.org/10.1016/j.rehab.2018.05.1321) | 165 | OECD (Netherlands) | EQ5D-3L |  |  |  |  | ✓ |  |  |  |  |  | 1 |  |  |
| [Kraai et al. (2013)](https://dx.doi.org/10.1093/eurjhf/hft071) | 100 | OECD (Netherlands) | EQ-VAS |  |  |  |  |  |  |  |  | ✓ |  | 1 |  |  |
| [Lindeman et al. (2018)](https://dx.doi.org/10.1097/SLA.0000000000002896) | 28 | OECD (Netherlands) | SF-36 |  |  |  |  |  |  |  |  |  | ✓ | 1 |  |  |
| [Oemrawsingh et al. (2019)](https://dx.doi.org/10.1186/s12874-019-0864-z) | 1022 | OECD (Netherlands) | EQ5D-3L |  |  |  |  | ✓ |  |  |  |  |  | 1 |  |  |
| [Petersohn et al. (2019)](https://dx.doi.org/10.1007/s11136-019-02166-0) | 229 | OECD (Netherlands) | EQ5D-3L |  |  |  |  |  |  |  |  |  | ✓ | 1 |  |  |
| [Schreuders et al. (2017)](https://dx.doi.org/10.1177/1747493017706244) | 457 | OECD (Netherlands) | EQ5D-3L |  |  |  |  | ✓ |  |  |  |  |  | 1 |  |  |
| [Timmermans et al. (2014)](https://dx.doi.org/10.1186/1743-0003-11-45) | 22 | OECD (Netherlands) | SF-36 |  |  |  |  | ✓ |  |  |  |  |  | 1 |  |  |
| [Vaidya et al. (2018)](https://dx.doi.org/10.2217/cer-2017-0029) | 204 | OECD (Netherlands) | EQ5D-3L |  |  |  |  |  |  |  |  |  | ✓ | 1 |  |  |
| [van den et al. (2017)](https://dx.doi.org/10.1056/NEJMoa1612136) | 197 | OECD (Netherlands) | EQ5D-3L |  |  |  |  |  | ✓ |  |  |  |  | 1 |  |  |
| [van et al. (2015)](https://dx.doi.org/10.1136/bmjopen-2015-008220) | 395 | OECD (Netherlands) | EQ5D-3L |  |  |  |  | ✓ |  |  |  |  |  | 1 |  |  |
| [van et al. (2020)](https://dx.doi.org/10.1186/s12913-020-05103-x) | 113 | OECD (Netherlands) | EQ5D-3L |  |  |  |  | ✓ | ✓ |  |  |  |  | 2 |  |  |
| [Verberne et al. (2021)](https://dx.doi.org/10.1136/bmjopen-2020-039201) | 306 | OECD (Netherlands) | EQ5D-3L |  |  |  |  | ✓ |  |  |  |  |  | 1 |  |  |
| [Faulkner et al. (2015)](https://dx.doi.org/10.1177/0269215514555729) | 28 | OECD (New Zealand) | SF-36 |  |  |  |  |  |  | ✓ |  |  |  | 1 |  |  |
| [Oh et al. (2017)](https://dx.doi.org/10.1016/j.wneu.2016.12.124) | 107 | OECD (North America) | SF-36 | ✓ | ✓ |  |  |  |  |  |  |  |  | 2 |  |  |
| [Bohmer et al. (2014)](https://dx.doi.org/10.3109/14017431.2014.923581) | 248 | OECD (Norway) | Other generic instrument |  |  | ✓ | ✓ |  |  |  |  |  |  | 2 |  |  |
| [Dohl et al. (2020)](https://dx.doi.org/10.1186/s12913-020-05158-w) | 194 | OECD (Norway) | EQ5D-5L |  |  |  |  | ✓ |  |  |  |  |  | 1 |  |  |
| [Hokstad et al. (2016)](https://dx.doi.org/10.2340/16501977-2051) | 261 | OECD (Norway) | EQ5D-5L |  |  |  |  | ✓ |  |  |  |  |  | 1 |  |  |
| [Labberton et al. (2020)](https://dx.doi.org/10.1007/s11136-020-02516-3) | 320 | OECD (Norway) | EQ5D-3L |  |  |  |  | ✓ |  | ✓ |  |  |  | 2 |  |  |
| [Larsen et al. (2017)](https://dx.doi.org/10.1186/s12955-017-0760-3) | 68 | OECD (Norway) | SF-36 |  |  |  |  |  |  |  |  |  | ✓ | 1 |  |  |
| [Lerdal et al. (2019)](https://dx.doi.org/10.1186/s41687-019-0142-1) | 220 | OECD (Norway) | EQ-VAS |  |  |  |  |  |  |  |  | ✓ |  | 1 |  |  |
| [Lunde (2013)](https://dx.doi.org/10.1007/s10198-012-0402-y) | 408 | OECD (Norway) | EQ-VAS |  |  |  |  |  | ✓ |  |  |  |  | 1 |  |  |
| [Sand et al. (2016)](https://dx.doi.org/10.1111/ene.12848) | 244 | OECD (Norway) | EQ5D-3L |  |  |  |  | ✓ |  |  |  |  |  | 1 |  |  |
| [Waehler et al. (2021)](https://dx.doi.org/10.1186/s12883-021-02128-5) | 625 | OECD (Norway) | EQ5D-5L |  |  |  |  | ✓ | ✓ |  |  |  |  | 2 |  |  |
| [Golicki et al. (2015a)](https://dx.doi.org/10.1007/s11136-014-0834-1) | 408 | OECD (Poland) | EQ5D-3L |  |  |  |  | ✓ |  |  |  |  |  | 1 |  |  |
| [Golicki et al. (2015b)](https://dx.doi.org/10.1007/s11136-014-0873-7) | 112 | OECD (Poland) | EQ5D-3L |  |  |  |  | ✓ |  |  |  |  |  | 1 |  |  |
| [Morys et al. (2015)](https://dx.doi.org/10.5603/CJ.a2015.0027) | 126 | OECD (Poland) | SF-36 | ✓ |  | ✓ |  |  |  |  |  | ✓ |  | 3 |  |  |
| [Niewada et al. (2021)](http://dx.doi.org/10.33963/KP.15885) | 345 | OECD (Poland) | SF-36 |  |  |  |  |  |  |  |  | ✓ |  | 1 |  |  |
| [Piotrowicz et al. (2015a)](https://dx.doi.org/10.1177/2047487314551537) | 111 | OECD (Poland) | SF-36 |  |  |  |  |  |  |  |  | ✓ |  | 1 |  |  |
| [Piotrowicz et al. (2015b)](https://dx.doi.org/10.1177/1474515114537023) | 56 | OECD (Poland) | SF-36 |  |  |  |  |  |  |  |  | ✓ |  | 1 |  |  |
| [Szygula-Jurkiewicz et al. (2014)](https://dx.doi.org/10.5114/kitp.2014.45678) | 202 | OECD (Poland) | SF-36 |  |  |  |  |  |  |  |  | ✓ |  | 1 |  |  |
| [Trystula (2018)](http://dx.doi.org/10.5604/01.3001.0011.7065) | 36 | OECD (Poland) | SF-36 |  |  |  |  |  |  | ✓ | ✓ |  |  | 2 |  |  |
| [Timoteo et al. (2020)](https://dx.doi.org/10.1016/j.repc.2019.09.013) | 70 | OECD (Portugal) | EQ5D-3L |  |  | ✓ | ✓ |  |  |  |  |  |  | 2 |  |  |
| [Alberca et al. (2019)](http://dx.doi.org/10.1016/j.jstrokecerebrovasdis.2018.09.046) | 92 | OECD (Spain) | EQ5D-5L |  |  |  |  |  |  | ✓ |  |  |  | 1 |  |  |
| [Alvarez-Sabin et al. (2016)](https://dx.doi.org/10.3390/ijms17030390) | 163 | OECD (Spain) | EQ5D-5L |  |  |  |  | ✓ | ✓ |  |  |  |  | 2 |  |  |
| [Arrospide et al. (2019)](https://dx.doi.org/10.1186/s12955-019-1134-9) | 423 | OECD (Spain) | EQ5D-5L |  |  | ✓ |  | ✓ |  |  |  |  |  | 2 |  |  |
| [Cano-Manas et al. (2020)](http://dx.doi.org/10.1155/2020/5480315) | 48 | OECD (Spain) | EQ-VAS |  |  |  |  | ✓ |  |  |  |  |  | 1 |  |  |
| [Comin-Colet et al. (2016)](https://dx.doi.org/10.1016/j.rec.2015.07.030) | 1037 | OECD (Spain) | EQ5D-3L |  |  |  |  |  |  |  |  | ✓ |  | 1 |  |  |
| [Davalos et al. (2017)](https://dx.doi.org/10.1016/S1474-4422(17)30047-9) | 103 | OECD (Spain) | EQ5D-5L |  |  |  |  | ✓ | ✓ |  |  |  |  | 2 |  |  |
| [Ding et al. (2019)](https://dx.doi.org/10.1161/JAHA.118.010988) | 3501 | OECD (Spain) | EQ-VAS |  |  | ✓ | ✓ |  |  |  |  |  |  | 2 | ✓ |  |
| [Dreyer et al. (2016)](https://dx.doi.org/10.1177/2048872615568967) | 3501 | OECD (Spain) | EQ5D-3L |  |  | ✓ |  |  |  |  |  |  |  | 1 |  |  |
| [Gonzalez-Guerrero et al. (2018)](http://dx.doi.org/10.1007/s41999-018-0074-y) | 58 | OECD (Spain) | EQ5D-3L |  |  |  |  |  |  |  |  | ✓ |  | 1 |  |  |
| [Lozano et al. (2013)](http://dx.doi.org/10.1016/j.angio.2013.01.012) | 1641 | OECD (Spain) | EQ5D-3L |  |  |  |  |  |  |  |  |  | ✓ | 1 |  |  |
| [Lozano et al. (2014a)](https://dx.doi.org/10.1111/ijcp.12499) | 920 | OECD (Spain) | EQ5D-3L |  |  |  |  |  |  |  |  |  | ✓ | 1 |  |  |
| [Lozano et al. (2014b)](https://dx.doi.org/10.1089/jwh.2013.4653) | 1641 | OECD (Spain) | EQ5D-3L |  |  |  |  |  |  |  |  |  | ✓ | 1 |  |  |
| [Mar et al. (2015)](https://dx.doi.org/10.1186/s12955-015-0230-8) | 321 | OECD (Spain) | EQ5D-3L |  |  |  |  | ✓ | ✓ |  |  |  |  | 2 |  |  |
| [Reverte-Villarroya et al. (2020)](https://dx.doi.org/10.3390/ijerph17176014) | 36 | OECD (Spain) | EQ5D-3L |  |  |  |  | ✓ | ✓ |  |  |  |  | 2 |  |  |
| [Rodriguez-Hernandez et al. (2021)](https://dx.doi.org/10.3390/ijerph18062810) | 20 | OECD (Spain) | EQ-VAS |  |  |  |  | ✓ |  |  |  |  |  | 1 |  |  |
| [Sanchez-Iriso et al. (2019)](https://dx.doi.org/10.1002/hec.3952) | 793 | OECD (Spain) | EQ5D-5L |  |  | ✓ |  | ✓ |  |  |  |  |  | 2 |  |  |
| [Vicent et al. (2017)](https://dx.doi.org/10.1186/s12904-017-0208-x) | 190 | OECD (Spain) | EQ5D-3L |  |  |  |  |  |  |  |  | ✓ |  | 1 |  |  |
| [Xu et al. (2015)](https://dx.doi.org/10.1161/CIRCULATIONAHA.114.012826) | 3572 | OECD (Spain, Australia and US) | SF-36 |  |  | ✓ |  |  |  |  |  |  |  | 1 |  |  |
| [Karlstrom et al. (2016)](https://dx.doi.org/10.1186/s12872-016-0221-7) | 132 | OECD (Sweden and Norway) | SF-36 |  |  |  |  |  |  |  |  | ✓ |  | 1 |  |  |
| [Agren et al. (2013)](https://dx.doi.org/10.1111/j.1365-2702.2012.04246.x) | 155 | OECD (Sweden) | SF-36 |  |  |  |  |  |  |  |  | ✓ |  | 1 |  |  |
| [Berg et al. (2015)](http://dx.doi.org/10.1016/j.jval.2015.02.003) | 5334 | OECD (Sweden) | EQ5D-3L |  |  |  |  |  |  |  |  | ✓ |  | 1 |  |  |
| [Dagner et al. (2019)](https://dx.doi.org/10.1177/1474515118783936) | 368 | OECD (Sweden) | EQ-VAS |  |  |  | ✓ |  |  |  |  |  |  | 1 |  |  |
| [Eriksson et al. (2013)](https://dx.doi.org/10.1111/j.1471-6712.2012.01032.x) | 13 | OECD (Sweden) | SF-36 |  |  | ✓ | ✓ |  |  |  |  |  |  | 2 |  |  |
| [Forsberg & (2013)](https://dx.doi.org/10.3138/ptc.2011-54) | 67 | OECD (Sweden) | SF-36 |  |  |  |  | ✓ |  |  |  |  |  | 1 |  |  |
| [Fransson et al. (2014)](https://dx.doi.org/10.1111/jocn.12492) | 127 | OECD (Sweden) | EQ-VAS |  |  | ✓ |  |  |  |  |  |  |  | 1 |  |  |
| [Hansson et al. (2013)](http://dx.doi.org/10.1111/j.1471-6712.2012.01041.x) | 283 | OECD (Sweden) | EQ5D-3L |  |  |  |  |  | ✓ |  |  |  |  | 1 |  |  |
| [Hansson et al. (2014)](http://dx.doi.org/10.1177/1474515114567035) | 123 | OECD (Sweden) | EQ5D-3L |  |  |  |  |  |  |  |  | ✓ |  | 1 |  |  |
| [Henriksson et al. (2014)](https://dx.doi.org/10.1136/openhrt-2014-000051) | 18012 | OECD (Sweden) | EQ5D-3L |  |  |  | ✓ |  |  |  |  |  |  | 1 |  |  |
| [Johnston et al. (2016)](https://dx.doi.org/10.1016/j.ahj.2016.05.005) | 71 | OECD (Sweden) | EQ-VAS |  |  | ✓ | ✓ |  |  |  |  |  |  | 2 |  |  |
| [Jonsson et al. (2014)](https://dx.doi.org/10.1161/STROKEAHA.114.005164) | 145 | OECD (Sweden) | EQ5D-3L |  |  |  |  | ✓ |  |  |  |  |  | 1 |  |  |
| [Kiadaliri et al. (2014)](https://dx.doi.org/10.3390/ijerph110504939) | 1757 | OECD (Sweden) | EQ5D-3L |  |  |  | ✓ | ✓ | ✓ |  |  |  |  | 3 | ✓ |  |
| [Lans et al. (2018)](https://dx.doi.org/10.1002/ehf2.12230) | 22 | OECD (Sweden) | SF-36 |  |  |  |  |  |  |  |  | ✓ |  | 1 |  |  |
| [Lawson et al. (2018)](https://dx.doi.org/10.1371/journal.pmed.1002540) | 10575 | OECD (Sweden) | EQ-VAS |  |  |  |  |  |  |  |  | ✓ |  | 1 |  | ✓ |
| [Lindgren et al. (2017a)](https://dx.doi.org/10.1177/1179546817747528) | 242 | OECD (Sweden) | EQ5D-3L |  |  |  |  |  |  |  |  |  | ✓ | 1 |  |  |
| [Lindgren et al. (2017b)](https://dx.doi.org/10.1016/j.ejvs.2017.01.026) | 49 | OECD (Sweden) | EQ5D-3L |  |  |  |  |  |  |  |  |  | ✓ | 1 |  |  |
| [Mejhert & (2015)](https://dx.doi.org/10.3109/13814788.2014.908282) | 46 | OECD (Sweden) | EQ5D-3L |  |  |  |  |  |  |  |  | ✓ |  | 1 |  |  |
| [Moren et al. (2016)](https://dx.doi.org/10.1097/NPT.0000000000000134) | 31 | OECD (Sweden) | EQ-VAS |  |  |  |  |  |  | ✓ | ✓ |  |  | 2 |  |  |
| [Nikolic et al. (2013)](https://dx.doi.org/10.1093/eurheartj/ehs149) | 559 | OECD (Sweden) | EQ5D-3L |  | ✓ | ✓ | ✓ | ✓ | ✓ |  |  |  |  | 5 |  |  |
| [Nordanstig et al. (2016)](https://dx.doi.org/10.1002/bjs.10198) | 79 | OECD (Sweden) | SF-36 |  |  |  |  |  |  |  |  |  | ✓ | 1 |  |  |
| [Palmcrantz et al. (2014)](https://dx.doi.org/10.1186/1471-2377-14-20) | 150 | OECD (Sweden) | EQ-VAS |  |  |  |  | ✓ | ✓ |  |  |  |  | 2 |  |  |
| [Palsdottir et al. (2020a)](https://dx.doi.org/10.2340/16501977-2652) | 499 | OECD (Sweden) | EQ5D-3L |  |  |  |  |  | ✓ |  |  |  |  | 1 |  |  |
| [Palsdottir et al. (2020b)](http://dx.doi.org/10.2340/16501977-2652) | 100 | OECD (Sweden) | EQ5D-3L |  |  |  |  | ✓ | ✓ |  |  |  |  | 2 |  |  |
| [Persson et al. (2017)](https://dx.doi.org/10.1186/s12955-017-0724-7) | 248 | OECD (Sweden) | SF-36 |  |  |  |  | ✓ |  |  |  |  |  | 1 |  |  |
| [Persson et al. (2020)](https://dx.doi.org/10.2147/COPD.S236192) | 56 | OECD (Sweden) | EQ5D-3L |  |  |  |  |  |  |  |  | ✓ |  | 1 |  |  |
| [Qvarfordt et al. (2018)](http://dx.doi.org/10.1007/s00270-018-1925-0) | 47 | OECD (Sweden) | EQ5D-3L |  |  |  |  |  |  |  |  |  | ✓ | 1 |  |  |
| [Rudberg et al. (2018)](https://dx.doi.org/10.1177/2396987317753444) | 297 | OECD (Sweden) | EQ5D-3L |  |  |  |  | ✓ | ✓ |  |  |  |  | 2 |  |  |
| [Sahlen et al. (2016)](https://dx.doi.org/10.1177/0269216315618544) | 36 | OECD (Sweden) | EQ5D-3L |  |  |  |  |  |  |  |  | ✓ |  | 1 |  |  |
| [Sandberg et al. (2016)](https://dx.doi.org/10.1016/j.apmr.2016.01.030) | 56 | OECD (Sweden) | EQ5D-3L |  |  |  |  | ✓ | ✓ |  |  |  |  | 2 |  |  |
| [Wu et al. (2013)](https://dx.doi.org/10.1177/1474515112468067) | 34 | OECD (Sweden) | SF-36 | ✓ | ✓ |  |  |  |  |  |  |  |  | 2 |  |  |
| [Anon. (2013)](http://dx.doi.org/10.1016/j.jchf.2012.08.002) | 228 | OECD (Switzerland) | SF-12 |  |  |  |  |  |  |  |  | ✓ |  | 1 |  |  |
| [Gencer et al. (2016)](https://dx.doi.org/10.1136/openhrt-2016-000419) | 1882 | OECD (Switzerland) | EQ5D-3L |  | ✓ | ✓ |  |  |  |  |  |  |  | 2 |  |  |
| [Schmid et al. (2013)](https://dx.doi.org/10.1111/j.1755-5922.2012.00313.x) | 21 | OECD (Switzerland) | SF-36 |  | ✓ |  |  |  |  |  |  |  |  | 1 |  |  |
| [Stauber et al. (2013)](https://dx.doi.org/10.1177/1358863X13505861) | 69 | OECD (Switzerland) | SF-36 |  |  |  |  |  |  |  |  |  | ✓ | 1 |  |  |
| [Dundar et al. (2014)](https://dx.doi.org/10.1310/tsr2106-453) | 71 | OECD (Turkey) | SF-36 |  |  |  |  | ✓ | ✓ |  |  |  |  | 2 |  |  |
| [Em et al. (2015)](http://dx.doi.org/10.5152/tftrd.2015.80090) | 104 | OECD (Turkey) | SF-36 |  |  |  |  | ✓ |  |  |  |  |  | 1 |  |  |
| [Erta & (2018)](http://dx.doi.org/10.5543/tkda.2017.66724) | 1034 | OECD (Turkey) | EQ5D-3L |  | ✓ | ✓ |  |  |  |  |  |  |  | 2 |  |  |
| [Karapolat et al. (2013)](https://dx.doi.org/10.1016/j.transproceed.2013.06.009) | 40 | OECD (Turkey) | SF-36 |  |  |  |  |  |  |  |  | ✓ |  | 1 |  |  |
| [Ozyemisci-Taskiran et al. (2019)](https://dx.doi.org/10.1080/10749357.2018.1550957) | 46 | OECD (Turkey) | SF-36 |  |  |  |  | ✓ |  |  |  |  |  | 1 |  |  |
| [Blum & (2014)](https://dx.doi.org/10.1016/j.cardfail.2014.04.016) | 204 | OECD (United States) | SF-36 |  |  |  |  |  |  |  |  | ✓ |  | 1 |  |  |
| [Bushnell et al. (2014)](https://dx.doi.org/10.1212/WNL.0000000000000208) | 1370 | OECD (United States) | EQ5D-3L |  |  |  |  | ✓ | ✓ | ✓ | ✓ |  |  | 4 |  |  |
| [Gaziano et al. (2016)](https://dx.doi.org/10.1001/jamacardio.2016.1747) | 8271 | OECD (United States) | EQ5D-3L |  |  |  |  |  |  |  |  | ✓ |  | 1 |  |  |
| [Gillard et al. (2015)](https://dx.doi.org/10.1186/s12955-015-0340-3) | 274 | OECD (United States) | EQ5D-3L |  |  |  |  | ✓ |  |  |  |  |  | 1 |  |  |
| [Grady et al. (2014)](https://dx.doi.org/10.1016/j.healun.2013.10.017) | 1559 | OECD (United States) | EQ5D-3L |  |  |  |  |  |  |  |  | ✓ |  | 1 |  |  |
| [Herman et al. (2018)](https://dx.doi.org/10.1016/j.jdiacomp.2018.05.007) | 77 | OECD (United States) | Other generic instrument | ✓ | ✓ | ✓ |  | ✓ |  | ✓ |  | ✓ |  | 6 |  |  |
| [Hickey et al. (2013)](https://dx.doi.org/10.1111/j.1365-2702.2012.04307.x) | 57 | OECD (United States) | SF-36 |  |  |  |  |  |  |  |  | ✓ |  | 1 |  |  |
| [Jelani et al. (2019)](http://dx.doi.org/10.1177/1358863X19872542) | 797 | OECD (United States) | EQ-VAS |  |  |  |  |  |  |  |  |  | ✓ | 1 |  |  |
| [Jelani et al. (2020)](https://dx.doi.org/10.1161/JAHA.119.014583) | 1243 | OECD (United States) | EQ-VAS |  |  |  |  |  |  |  |  |  | ✓ | 1 |  |  |
| [Jia & (2016)](https://dx.doi.org/10.1007/s11136-016-1226-5) | 255 | OECD (United States) | EQ5D-3L |  |  |  |  | ✓ |  |  |  |  |  | 1 |  |  |
| [Jia et al. (2018)](https://dx.doi.org/10.1097/MLR.0000000000000943) | 37194 | OECD (United States) | EQ5D-3L | ✓ | ✓ | ✓ | ✓ | ✓ | ✓ |  |  | ✓ |  | 7 |  |  |
| [Kang et al. (2018)](https://dx.doi.org/10.1177/1099800418772346) | 27 | OECD (United States) | SF-36 |  |  |  |  |  |  |  |  | ✓ |  | 1 |  |  |
| [Katzan et al. (2017)](https://dx.doi.org/10.1161/JAHA.116.005356) | 3283 | OECD (United States) | EQ5D-3L |  |  |  |  | ✓ |  |  |  |  |  | 1 |  |  |
| [Luo et al. (2015)](https://dx.doi.org/10.1111/jgs.13796) | 963 | OECD (United States) | SF-36 |  |  |  |  | ✓ |  |  |  |  |  | 1 |  |  |
| [Magnuson et al. (2021)](http://dx.doi.org/10.1093/ehjqcco/qcab014) | 3925 | OECD (United States) | EQ5D-5L |  |  |  |  |  |  |  |  | ✓ |  | 1 |  |  |
| [Martinson et al. (2017)](https://dx.doi.org/10.1002/ejhf.642) | 270 | OECD (United States) | EQ5D-3L |  |  |  |  |  |  |  |  | ✓ |  | 1 |  |  |
| [Morey et al. (2021)](https://dx.doi.org/10.1161/CIRCOUTCOMES.120.006769) | 11570 | OECD (United States) | SF-36 | ✓ | ✓ | ✓ | ✓ | ✓ | ✓ |  |  | ✓ | ✓ | 8 |  |  |
| [Mustapha et al. (2019)](https://dx.doi.org/10.1177/1526602819827295) | 1204 | OECD (United States) | EQ-VAS |  |  |  |  |  |  |  |  |  | ✓ | 1 |  |  |
| [Pokharel et al. (2017)](https://dx.doi.org/10.1016/j.atherosclerosis.2017.09.019) | 3410 | OECD (United States) | EQ-VAS |  |  | ✓ | ✓ |  |  |  |  |  |  | 2 |  |  |
| [Poole et al. (2013)](https://dx.doi.org/10.1001/jama.2013.282540) | 159 | OECD (United States) | SF-36 |  |  |  |  |  |  |  |  |  | ✓ | 1 |  |  |
| [Rangaraju et al. (2016)](https://dx.doi.org/10.1159/000443801) | 587 | OECD (United States) | EQ5D-3L |  |  |  |  | ✓ |  |  |  |  |  | 1 |  |  |
| [Rangaraju et al. (2017)](https://dx.doi.org/10.1159/000452634) | 423 | OECD (United States) | EQ5D-3L |  |  |  |  | ✓ |  |  |  |  |  | 1 |  |  |
| [Reed et al. (2013)](https://dx.doi.org/10.1016/j.cardfail.2013.07.003) | 3459 | OECD (United States) | EQ5D-3L |  |  | ✓ | ✓ |  |  |  |  |  |  | 2 |  |  |
| [Salisbury et al. (2018)](https://dx.doi.org/10.1016/j.jvn.2017.09.003) | 46 | OECD (United States) | SF-36 |  |  |  |  |  |  |  |  |  | ✓ | 1 |  |  |
| [Shao et al. (2019)](https://dx.doi.org/10.1007/s40273-019-00775-8) | 2766 | OECD (United States) | HUI3 | ✓ | ✓ | ✓ |  | ✓ |  |  |  |  |  | 4 | ✓ |  |
| [Simpson et al. (2015)](https://dx.doi.org/10.12715/har.2015.4.4) | 2567 | OECD (United States) | EQ-VAS | ✓ | ✓ | ✓ |  |  |  |  |  |  |  | 3 |  |  |
| [Stehlik et al. (2017)](http://dx.doi.org/10.1161/CIRCHEARTFAILURE.116.003910) | 103 | OECD (United States) | EQ-VAS |  |  |  |  |  |  |  |  | ✓ |  | 1 |  |  |
| [Stewart et al. (2016)](http://ovidsp.ovid.com/ovidweb.cgi?T=JS&PAGE=reference&D=med13&NEWS=N&AN=27834198) | 166 | OECD (United States) | EQ5D-3L |  |  |  |  |  |  |  |  | ✓ |  | 1 |  |  |
| [Zanaboni et al. (2013)](https://dx.doi.org/10.2196/jmir.2587) | 200 | OECD (United States) | EQ-VAS |  |  |  |  |  |  |  |  | ✓ |  | 1 |  |  |
| [Hays et al. (2014)](https://dx.doi.org/10.1007/s11136-013-0503-9) | 47371 | OECD (USA) | SF-36 | ✓ |  | ✓ |  |  |  |  |  | ✓ |  | 3 | ✓ |  |
| [Zannad et al. (2015)](https://dx.doi.org/10.1093/eurheartj/ehu345) | 85 | Other (Across West Europe) | SF-36 |  |  |  |  |  |  |  |  | ✓ |  | 1 |  |  |
| [Dzubur et al. (2016)](https://dx.doi.org/10.5455/medarh.2016.70.419-424) | 40 | Other (Bosnia and Herzegovina) | SF-36 |  |  | ✓ |  |  |  |  |  |  |  | 1 |  |  |
| [Beinotti et al. (2013)](https://dx.doi.org/10.1310/tsr2003-226) | 24 | Other (Brazil) | SF-36 |  |  |  |  |  | ✓ |  |  |  |  | 1 |  |  |
| [do Nascimento et al. (2015)](http://dx.doi.org/10.1590/1677-5449.20140043) | 10 | Other (Brazil) | SF-36 |  |  |  |  |  |  |  |  |  | ✓ | 1 | ✓ |  |
| [Jorge et al. (2017)](https://dx.doi.org/10.5935/abc.20170123) | 59 | Other (Brazil) | SF-36 |  |  |  |  |  |  |  |  | ✓ |  | 1 |  |  |
| [Martins et al. (2021)](https://dx.doi.org/10.1080/10749357.2020.1805244) | 37 | Other (Brazil) | EQ5D-3L |  |  |  |  | ✓ |  |  |  |  |  | 1 |  |  |
| [Molle Da et al. (2019)](https://dx.doi.org/10.1080/10749357.2019.1631605) | 38 | Other (Brazil) | EQ5D-3L |  |  |  |  | ✓ |  |  |  |  |  | 1 |  |  |
| [Silva et al. (2020)](https://dx.doi.org/10.1080/10749357.2020.1864964) | 90 | Other (Brazil) | EQ-VAS |  |  |  |  |  | ✓ |  |  |  |  | 1 |  |  |
| [Chen et al. (2019)](https://dx.doi.org/10.1097/MD.0000000000015130) | 136 | Other (China) | SF-36 |  |  |  |  | ✓ |  |  |  |  |  | 1 |  |  |
| [Chen et al. (2020)](https://dx.doi.org/10.1161/STROKEAHA.119.027639) | 4016 | Other (China) | EQ5D-3L |  |  |  |  | ✓ |  |  |  |  |  | 1 |  |  |
| [Dong et al. (2017)](https://dx.doi.org/10.1007/s40520-016-0614-6) | 132 | Other (China) | SF-36 |  |  |  |  |  |  |  |  | ✓ |  | 1 |  |  |
| [Dreyer et al. (2019)](https://dx.doi.org/10.1177/2048872618803726) | 3415 | Other (China) | EQ5D-3L |  |  | ✓ | ✓ |  |  |  |  |  |  | 2 |  |  |
| [Fu et al. (2016)](https://dx.doi.org/10.1186/s12906-016-1306-7) | 115 | Other (China) | SF-36 |  |  |  |  |  |  |  |  | ✓ |  | 1 |  |  |
| [Gu et al. (2020)](https://dx.doi.org/10.1007/s11136-020-02524-3) | 353 | Other (China) | EQ5D-3L |  |  | ✓ | ✓ | ✓ | ✓ |  |  | ✓ |  | 5 |  |  |
| [Hsu et al. (2019)](https://dx.doi.org/10.4103/tcmj.tcmj_95_18) | 45 | Other (China) | EQ5D-3L |  |  |  |  | ✓ | ✓ |  |  |  |  | 2 |  |  |
| [Jackson et al. (2018)](https://dx.doi.org/10.2147/DDDT.S148949) | 933 | Other (China) | EQ5D-3L |  |  |  |  |  |  |  |  | ✓ |  | 1 |  |  |
| [Jiao et al. (2017)](http://dx.doi.org/10.1186/s12955-017-0699-4) | 72 | Other (China) | SF-12 |  |  |  |  | ✓ |  |  |  |  |  | 1 | ✓ |  |
| [Lawrie et al. (2018)](https://dx.doi.org/10.1186/s40814-018-0345-x) | 20 | Other (China) | EQ-VAS |  |  |  |  | ✓ |  |  |  |  |  | 1 |  |  |
| [Liu et al. (2020)](http://dx.doi.org/10.1111/eci.13261) | 350 | other (China) | EQ5D-3L |  |  |  |  |  |  |  |  | ✓ |  | 1 |  |  |
| [Mei et al. (2021)](https://dx.doi.org/10.1038/s41598-021-84554-6) | 208 | Other (China) | EQ5D-5L |  |  |  |  | ✓ |  |  |  |  |  | 1 |  |  |
| [Pan et al. (2018)](https://dx.doi.org/10.1016/j.archger.2018.01.008) | 104 | Other (China) | EQ5D-3L |  |  |  |  | ✓ |  |  |  |  |  | 1 |  |  |
| [Rethnam et al. (2020)](https://dx.doi.org/10.1177/1747493019830583) | 406 | Other (China) | EQ5D-5L | ✓ | ✓ | ✓ |  |  |  |  |  |  |  | 3 |  |  |
| [Sun et al. (2019)](https://dx.doi.org/10.12998/wjcc.v7.i16.2165) | 50 | Other (China) | SF-36 |  |  |  |  |  |  |  |  | ✓ |  | 1 |  |  |
| [Wang et al. (2014a)](https://dx.doi.org/10.1177/2047487312454757) | 192 | Other (China) | SF-36 |  |  | ✓ |  |  |  |  |  |  |  | 1 |  |  |
| [Wang et al. (2014b)](https://dx.doi.org/10.1111/cns.12329) | 5104 | Other (China) | EQ5D-3L |  |  |  |  |  |  | ✓ |  |  |  | 1 |  |  |
| [Wang et al. (2015a)](https://dx.doi.org/10.4103/0366-6999.160486) | 263 | Other (China) | EQ5D-3L |  |  |  |  | ✓ |  |  |  |  |  | 1 |  |  |
| [Wu et al. (2014a)](https://dx.doi.org/10.1186/s12955-014-0156-6) | 411 | Other (China) | EQ5D-3L | ✓ |  |  |  |  |  |  |  |  |  | 1 |  |  |
| [Wu et al. (2014b)](https://dx.doi.org/10.1371/journal.pone.0097294) | 411 | Other (China) | EQ5D-3L | ✓ |  |  |  |  |  |  |  |  |  | 1 |  |  |
| [Wu et al. (2014c)](https://dx.doi.org/10.1016/j.jocn.2013.08.030) | 386 | Other (China) | SF-36 |  |  |  |  | ✓ | ✓ |  |  |  |  | 2 |  |  |
| [Wu et al. (2015b)](https://dx.doi.org/10.1111/jocn.12926) | 217 | Other (China) | SF-36 |  |  |  |  | ✓ | ✓ |  |  |  |  | 2 |  |  |
| [Xian et al. (2016)](https://dx.doi.org/10.1016/j.jep.2016.03.066) | 109 | Other (China) | SF-36 |  |  |  |  |  |  |  |  | ✓ |  | 1 |  |  |
| [Zhang et al. (2018)](http://dx.doi.org/10.14704/nq.2018.16.5.1327) | 71 | Other (China) | SF-36 |  |  |  |  | ✓ |  |  |  |  |  | 1 |  |  |
| [Zhang et al. (2020)](https://dx.doi.org/10.1007/s13300-020-00788-z) | 590 | Other (China) | EQ5D-3L |  |  |  |  | ✓ |  |  |  |  |  | 1 | ✓ |  |
| [Zhang et al. (2021)](http://dx.doi.org/10.1111/liv.14780) | 3718 | Other (China) | HUI2 |  |  |  | ✓ |  |  |  |  |  |  | 1 |  |  |
| [Zhen et al. (2016)](https://dx.doi.org/10.3389/fneur.2016.00235) | 10 | Other (China) | SF-36 |  |  |  |  | ✓ |  |  |  |  |  | 1 |  |  |
| [Zhou et al. (2019)](http://dx.doi.org/10.1161/STROKEAHA.118.021558) | 226 | Other (China) | EQ5D-3L |  |  |  |  | ✓ |  |  |  |  |  | 1 |  |  |
| [Rosic et al. (2021)](https://www.researchgate.net/publication/347937258_The_Role_of_Ambulatory_Cardiac_Rehabilitation_in_Improvement_of_Quality_of_Life_Anxiety_and_Depression) | 38 | Other (Croatia) | SF-36 |  |  |  |  |  |  |  |  | ✓ |  | 1 |  |  |
| [Romero-Naranjo et al. (2019)](http://ovidsp.ovid.com/ovidweb.cgi?T=JS&PAGE=reference&D=med16&NEWS=N&AN=31260554) | 47 | Other (Ecuador) | EQ5D-3L |  |  |  |  |  |  |  |  |  | ✓ | 1 | ✓ |  |
| [Amer et al. (2013)](https://dx.doi.org/10.1111/j.1447-0594.2012.00928.x) | 30 | Other (Egypt) | SF-36 |  |  |  |  |  |  |  |  |  | ✓ | 1 | ✓ |  |
| [Wong et al. (2018)](https://dx.doi.org/10.1007/s11136-018-1887-3) | 21 | Other (Hong Kong) | SF-36 |  |  |  |  | ✓ |  |  |  |  |  | 1 |  |  |
| [Chandrasekaran et al. (2020)](http://dx.doi.org/10.1016/j.jacc.2020.01.050) | 1968 | Other (India) | EQ-VAS |  |  | ✓ |  |  |  |  |  |  |  | 1 |  |  |
| [Chandrasekhar et al. (2018)](http://dx.doi.org/10.1016/j.cegh.2018.05.001) | 64 | Other (India) | SF-36 |  |  |  |  | ✓ |  |  |  |  |  | 1 |  |  |
| [Andayani et al. (2020)](http://dx.doi.org/10.29090/psa.2020.03.019.0040) | 21 | Other (Indonesia) | EQ5D-5L |  |  |  |  | ✓ |  |  |  |  |  | 1 | ✓ |  |
| [Hayes et al. (2016)](https://dx.doi.org/10.1016/j.jval.2015.10.010) | 335 | Other (International inc. UK) | EQ5D-3L |  |  |  |  | ✓ |  |  |  |  |  | 1 | ✓ |  |
| [Huber et al. (2016)](http://ovidsp.ovid.com/ovidweb.cgi?T=JS&PAGE=reference&D=med13&NEWS=N&AN=27318487) | 5508 | Other (International inc. UK) | SF-36 | ✓ |  | ✓ |  |  |  |  |  | ✓ |  | 3 |  |  |
| [Nauck et al. (2019)](https://dx.doi.org/10.1111/dom.13547) | 369 | Other (International inc. UK) | EQ5D-3L |  |  | ✓ |  | ✓ |  |  |  | ✓ |  | 3 | ✓ |  |
| [Renton et al. (2014)](http://dx.doi.org/10.1016/j.jstrokecerebrovasdis.2014.04.022) | 1571 | Other (International inc. UK) | EQ5D-3L |  |  |  |  | ✓ |  |  |  |  |  | 1 |  |  |
| [Sandercock (2013)](http://dx.doi.org/10.1016/S1474-4422%2813%2970130-3) | 662 | Other (International inc. UK) | EQ5D-3L |  |  |  |  | ✓ |  |  |  |  |  | 1 |  |  |
| [Stummer et al. (2015)](https://dx.doi.org/10.3109/09638288.2014.948137) | 532 | Other (International inc. UK) | EQ-VAS |  |  |  |  | ✓ |  |  |  |  |  | 1 |  |  |
| [Whynes et al. (2013)](https://dx.doi.org/10.1177/0272989X12465016) | 1462 | Other (International inc. UK) | EQ5D-3L |  |  |  |  | ✓ |  |  |  |  |  | 1 |  |  |
| [Ambrosy et al. (2016)](https://dx.doi.org/10.1002/ejhf.420) | 6943 | Other (International) | EQ5D-3L |  |  |  |  |  |  |  |  | ✓ |  | 1 |  |  |
| [Ghanbari-Firoozabadi et al. (2014)](http://ovidsp.ovid.com/ovidweb.cgi?T=JS&PAGE=reference&D=med11&NEWS=N&AN=24653758) | 34 | Other (Iran) | SF-36 |  |  |  |  |  |  |  |  | ✓ |  | 1 |  |  |
| [Mehralian et al. (2014)](https://dx.doi.org/10.5539/gjhs.v6n3p256) | 50 | Other (Iran) | SF-36 |  |  |  |  |  |  |  |  | ✓ |  | 1 |  |  |
| [Rahmani et al. (2020)](https://dx.doi.org/10.1155/2020/8897881) | 70 | Other (Iran) | SF-36 |  |  |  |  |  |  |  |  | ✓ |  | 1 |  |  |
| [Rajati et al. (2016)](https://dx.doi.org/10.1016/j.apmr.2016.05.010) | 60 | Other (Iran) | SF-36 |  |  |  |  |  |  |  |  | ✓ |  | 1 |  |  |
| [Vahedian-Azimi et al. (2016)](https://dx.doi.org/10.1136/openhrt-2015-000349) | 70 | Other (Iran) | SF-36 |  |  | ✓ | ✓ |  |  |  |  |  |  | 2 |  |  |
| [Gordon et al. (2013)](https://dx.doi.org/10.1161/STROKEAHA.111.000642) | 64 | Other (Jamaica) | SF-36 |  |  |  |  | ✓ |  |  |  |  |  | 1 |  |  |
| [Muli & (2013)](https://dx.doi.org/10.4314/ahs.v13i3.16) | 161 | Other (Kenya) | SF-36 |  |  |  |  | ✓ |  |  |  |  |  | 1 |  |  |
| [Chimatiro et al. (2018)](https://dx.doi.org/10.4314/mmj.v30i3.4) | 114 | Other (Malawi) | EQ-VAS |  |  |  |  | ✓ |  |  |  |  |  | 1 |  |  |
| [Abdul et al. (2020)](https://dx.doi.org/10.1186/s12877-020-1453-z) | 65 | Other (Malaysia) | EQ5D-5L |  |  |  |  | ✓ | ✓ |  |  |  |  | 2 |  |  |
| [Azmi et al. (2015)](https://dx.doi.org/10.1016/j.vhri.2015.03.015) | 45 | Other (Malaysia) | EQ-VAS |  | ✓ |  |  |  |  |  |  |  |  | 1 |  |  |
| [Adibe et al. (2013)](https://dx.doi.org/10.1016/j.vhri.2013.06.007) | 11 | Other (Nigeria) | HUI2 |  |  | ✓ |  | ✓ |  |  |  |  |  | 2 | ✓ |  |
| [Pogosova et al. (2014)](https://doi.org/10.20996/1819-6446-2014-10-6-584-596) | 322 | Other (Russia) | SF-36 | ✓ |  | ✓ |  |  |  |  |  | ✓ |  | 3 |  |  |
| [Jovanic et al. (2018)](https://dx.doi.org/10.3390/ijerph15081761) | 200 | Other (Serbia) | SF-36 |  |  |  |  |  |  |  |  | ✓ |  | 1 |  |  |
| [Kocic et al. (2020)](http://dx.doi.org/10.3390/medicina56120666) | 160 | Other (Serbia) | EQ-VAS |  |  |  |  | ✓ | ✓ |  |  |  |  | 2 |  |  |
| [Maksimovic et al. (2014)](https://dx.doi.org/10.1177/0003319713488640) | 102 | Other (Serbia) | SF-36 |  |  |  |  |  |  |  |  |  | ✓ | 1 |  |  |
| [Rancic et al. (2013)](http://dx.doi.org/10.2478/s11536-012-0118-5) | 160 | Other (Serbia) | EQ-VAS |  |  | ✓ |  |  |  |  |  |  |  | 1 |  |  |
| [Stojanovic et al. (2018)](https://dx.doi.org/10.21101/cejph.a5022) | 209 | Other (Serbia) | EQ-VAS | ✓ | ✓ | ✓ |  | ✓ |  | ✓ |  | ✓ | ✓ | 7 | ✓ |  |
| [Tasic et al. (2014)](http://dx.doi.org/10.2478/s11536-013-0186-1) | 745 | Other (Serbia) | SF-36 |  |  | ✓ | ✓ |  |  |  |  |  |  | 2 |  |  |
| [Vlajinac et al. (2014)](https://dx.doi.org/10.1007/s00508-014-0663-9) | 102 | Other (Serbia) | SF-36 |  |  |  |  |  |  |  |  |  | ✓ | 1 |  |  |
| [Abdin et al. (2015)](https://dx.doi.org/10.1007/s11136-014-0859-5) | 157 | Other (Singapore) | EQ5D-3L |  |  | ✓ |  | ✓ |  |  |  |  |  | 2 |  |  |
| [Cheung et al. (2019)](https://dx.doi.org/10.1007/s11136-019-02254-1) | 473 | Other (Singapore) | HUI3 |  |  |  |  | ✓ | ✓ |  |  |  |  | 2 |  |  |
| [Guo et al. (2017)](http://dx.doi.org/10.1080/02687038.2016.1261269) | 78 | Other (Singapore) | EQ5D-3L |  |  |  |  | ✓ | ✓ |  |  |  |  | 2 |  |  |
| [Leow et al. (2013)](https://dx.doi.org/10.1371/journal.pone.0067138) | 111 | Other (Singapore) | SF-36 |  |  |  |  |  | ✓ |  |  |  |  | 1 |  |  |
| [Venkataraman et al. (2013)](https://dx.doi.org/10.1111/j.1365-2265.2012.04480.x) | 45 | Other (Singapore) | SF-36 |  |  |  |  | ✓ |  |  |  |  | ✓ | 2 | ✓ |  |
| [Wang et al. (2015b)](https://dx.doi.org/10.1007/s11239-015-1191-9) | 100 | Other (Singapore) | Directly elicited (SG) |  |  |  |  | ✓ |  |  |  |  |  | 1 |  |  |
| [Yeoh et al. (2018)](https://dx.doi.org/10.1186/s12955-018-1043-3) | 380 | Other (Singapore) | EQ5D-3L |  |  |  |  | ✓ | ✓ |  |  |  |  | 2 |  |  |
| [Yeoh et al. (2019)](https://dx.doi.org/10.1371/journal.pone.0211493) | 464 | Other (Singapore) | EQ5D-3L |  |  |  |  | ✓ | ✓ |  |  |  |  | 2 |  |  |
| [Tusek-Bunc & (2016)](http://ovidsp.ovid.com/ovidweb.cgi?T=JS&PAGE=reference&D=med13&NEWS=N&AN=27846850) | 423 | Other (Slovenia) | EQ5D-3L |  |  |  |  |  |  |  |  | ✓ | ✓ | 2 |  |  |
| [Rhoda (2014)](https://dx.doi.org/10.4102/ajod.v3i1.126) | 73 | Other (South Africa) | EQ5D-3L |  |  |  |  |  | ✓ |  |  |  |  | 1 |  |  |
| [Mahesh et al. (2018)](https://dx.doi.org/10.1007/s10072-017-3172-6) | 210 | Other (Sri Lanka) | SF-36 |  |  |  |  | ✓ |  |  |  |  |  | 1 |  |  |
| [Chen et al. (2015)](https://dx.doi.org/10.1016/j.jstrokecerebrovasdis.2015.02.002) | 35 | Other (Taiwan) | SF-36 |  |  |  |  | ✓ |  |  |  |  |  | 1 |  |  |
| [Chen et al. (2016)](https://dx.doi.org/10.1007/s11136-015-1196-z) | 65 | Other (Taiwan) | EQ5D-3L |  |  |  |  | ✓ |  |  |  |  |  | 1 |  |  |
| [Fu et al. (2015)](https://dx.doi.org/10.1536/ihj.15-012) | 157 | Other (Taiwan) | SF-36 |  |  |  |  |  |  |  |  | ✓ |  | 1 |  |  |
| [Hsieh et al. (2018)](https://dx.doi.org/10.1016/j.apmr.2018.03.017) | 24 | Other (Taiwan) | EQ-VAS |  |  |  |  | ✓ |  |  |  |  |  | 1 |  |  |
| [Kang et al. (2019)](https://dx.doi.org/10.1186/s12911-019-1000-z) | 76 | Other (Taiwan) | EQ5D-3L |  |  |  |  | ✓ |  |  |  |  |  | 1 |  |  |
| [Kuo et al. (2019)](https://dx.doi.org/10.1080/13607863.2017.1414148) | 592 | Other (Taiwan) | EQ5D-3L |  |  |  |  | ✓ |  |  |  |  |  | 1 |  |  |
| [Kuo et al. (2021)](https://dx.doi.org/10.1111/jdi.13520) | 2104 | Other (Taiwan) | EQ5D-3L |  |  |  |  | ✓ | ✓ | ✓ | ✓ | ✓ |  | 5 |  |  |
| [Lai et al. (2017)](https://dx.doi.org/10.2147/PPA.S136041) | 168 | Other (Taiwan) | EQ5D-3L |  |  |  |  | ✓ |  |  |  |  |  | 1 |  |  |
| [Lu et al. (2016)](https://dx.doi.org/10.2340/16501977-2069) | 478 | Other (Taiwan) | EQ5D-3L |  |  |  |  | ✓ | ✓ |  |  |  |  | 2 |  |  |
| [Peng et al. (2019)](https://dx.doi.org/10.1016/j.archger.2019.04.011) | 1522 | Other (Taiwan) | EQ5D-3L |  |  |  |  | ✓ | ✓ |  |  |  |  | 2 |  |  |
| [Teng et al. (2018)](https://dx.doi.org/10.1177/1474515118778453) | 84 | Other (Taiwan) | EQ-VAS |  |  |  |  |  |  |  |  | ✓ |  | 1 |  |  |
| [Tsai et al. (2019)](https://dx.doi.org/10.6515/ACS.201907_35(4).20190330A) | 42 | Other (Taiwan) | SF-36 |  |  | ✓ | ✓ |  |  |  |  |  |  | 2 |  |  |
| [Yu et al. (2019)](https://dx.doi.org/10.1007/s40520-018-1099-2) | 45 | Other (Taiwan) | SF-36 |  |  |  |  | ✓ |  |  |  |  |  | 1 |  |  |
| [Rachpukdee et al. (2013)](https://dx.doi.org/10.1016/j.jstrokecerebrovasdis.2012.05.005) | 125 | Other (Thailand) | SF-36 |  |  |  |  | ✓ |  |  |  |  |  | 1 |  |  |
| [Levytska et al. (2016)](http://ovidsp.ovid.com/ovidweb.cgi?T=JS&PAGE=reference&D=emed17&NEWS=N&AN=616515376) | 29 | Other (Ukraine) | SF-36 |  |  |  |  | ✓ | ✓ |  |  |  |  | 2 |  |  |
| [Agus et al. (2016)](https://dx.doi.org/10.1136/heartjnl-2015-308247) | 243 | UK | EQ5D-3L | ✓ |  |  |  |  |  |  |  |  |  | 1 |  |  |
| [Ali et al. (2017)](https://dx.doi.org/10.1177/2396987316683780) | 3858 | UK | EQ5D-3L |  |  |  |  | ✓ |  |  |  |  |  | 1 |  |  |
| [Alva et al. (2014)](https://dx.doi.org/10.1002/hec.2930) | 771 | UK | EQ5D-3L |  |  | ✓ |  | ✓ |  |  |  | ✓ |  | 3 | ✓ |  |
| [Ankolekar et al. (2014)](https://dx.doi.org/10.1016/j.jstrokecerebrovasdis.2014.04.022) | 1573 | UK | EQ5D-3L |  |  |  |  | ✓ |  |  |  |  |  | 1 |  |  |
| [Ayis et al. (2015)](https://dx.doi.org/10.1136/bmjopen-2014-007101) | 1848 | UK | SF-12 |  |  |  |  |  | ✓ |  |  |  |  | 1 |  |  |
| [Babber et al. (2020)](https://dx.doi.org/10.1002/bjs.11398) | 22 | UK | EQ5D-5L |  |  |  |  |  |  |  |  |  | ✓ | 1 |  |  |
| [Bath et al. (2017)](https://dx.doi.org/10.1371/journal.pone.0164608) | 83 | UK | EQ-VAS |  |  |  |  | ✓ | ✓ |  |  |  |  | 2 |  |  |
| [Briggs et al. (2017)](https://dx.doi.org/10.1016/j.diabres.2016.12.019) | 623 | UK | EQ5D-3L |  |  | ✓ |  | ✓ |  |  |  |  |  | 2 | ✓ |  |
| [Burton et al. (2014)](https://dx.doi.org/10.1186/1472-6963-14-63) | 80 | UK | EQ-VAS |  |  |  |  |  | ✓ |  |  |  |  | 1 |  |  |
| [Campbell et al. (2018)](http://dx.doi.org/10.1002/ejhf.1240) | 272 | UK | SF-12 |  |  |  |  |  |  |  |  | ✓ |  | 1 |  |  |
| [De et al. (2017)](https://dx.doi.org/10.1080/09638288.2016.1200676) | 226 | UK | EQ-VAS |  |  |  |  |  | ✓ |  |  |  |  | 1 |  |  |
| [Ezeofor et al. (2021)](https://dx.doi.org/10.1371/journal.pone.0244851) | 27 | UK | EQ5D-5L |  |  |  |  |  |  |  |  |  | ✓ | 1 |  |  |
| [Farndon et al. (2018)](https://dx.doi.org/10.1186/s13047-018-0269-y) | 18 | UK | EQ-VAS |  |  |  |  |  |  |  |  |  | ✓ | 1 |  |  |
| [Ford et al. (2018)](https://dx.doi.org/10.1016/j.jacc.2018.09.006) | 75 | UK | EQ5D-5L | ✓ |  |  |  |  |  |  |  |  |  | 1 |  |  |
| [Forster et al. (2015)](https://dx.doi.org/10.1161/STROKEAHA.115.008585) | 800 | UK | EQ5D-3L |  |  |  |  | ✓ | ✓ |  |  |  |  | 2 |  |  |
| [Gallagher et al. (2019)](https://dx.doi.org/10.1002/ehf2.12363) | 152 | UK | EQ5D-3L |  |  |  |  |  |  |  |  | ✓ |  | 1 |  |  |
| [Green et al. (2018)](https://dx.doi.org/10.1177/1708538118773618) | 15 | UK | EQ5D-3L |  |  |  |  |  |  |  |  |  | ✓ | 1 |  |  |
| [Hurdus et al. (2020)](https://dx.doi.org/10.1136/heartjnl-2020-316920) | 4570 | UK | EQ5D-3L |  |  | ✓ | ✓ |  |  |  |  |  |  | 2 |  |  |
| [Janssen et al. (2013)](https://dx.doi.org/10.1007/s11136-012-0322-4) | 160 | UK | EQ-VAS |  |  | ✓ |  | ✓ |  |  |  |  |  | 2 |  |  |
| [Jenkinson et al. (2013)](https://dx.doi.org/10.1161/STROKEAHA.113.001847) | 73 | UK | EQ5D-3L |  |  |  |  | ✓ |  |  |  |  |  | 1 |  |  |
| [Jones et al. (2016)](http://dx.doi.org/10.1136/bmjopen-2015-008900) | 78 | UK | SF-12 |  |  |  |  | ✓ |  |  |  |  |  | 1 |  |  |
| [Kirk et al. (2014)](https://dx.doi.org/10.1177/0269215513502211) | 24 | UK | SF-36 |  |  |  |  |  |  | ✓ | ✓ |  |  | 2 |  |  |
| [Lewis et al. (2014)](https://dx.doi.org/10.1016/j.jchf.2013.12.003) | 1785 | UK | EQ5D-3L |  |  | ✓ |  |  |  |  |  |  |  | 1 |  |  |
| [Logan et al. (2014)](https://dx.doi.org/10.3310/hta18290) | 568 | UK | EQ5D-3L |  |  |  |  |  | ✓ |  |  |  |  | 1 |  |  |
| [Luengo-Fernandez et al. (2013)](https://dx.doi.org/10.1212/WNL.0b013e3182a9f45f) | 759 | UK | EQ5D-3L |  |  |  |  | ✓ | ✓ | ✓ | ✓ |  |  | 4 |  |  |
| [Matza et al. (2015)](https://dx.doi.org/10.1186/s12913-015-0772-9) | 200 | UK | Directly elicited (TTO) |  |  |  |  | ✓ |  |  |  | ✓ |  | 2 |  |  |
| [McCreanor et al. (2021)](https://dx.doi.org/10.1136/bmjopen-2020-044054) | 195 | UK | EQ5D-5L | ✓ |  |  |  |  |  |  |  |  |  | 1 |  |  |
| [Mejia et al. (2014)](https://dx.doi.org/10.1016/j.ijnurstu.2014.01.009) | 260 | UK | EQ5D-3L |  |  |  |  |  |  |  |  | ✓ |  | 1 |  |  |
| [Monahan et al. (2017)](https://dx.doi.org/10.1016/j.ijcard.2017.02.149) | 304 | UK | EQ5D-3L |  |  |  |  |  |  |  |  | ✓ |  | 1 |  |  |
| [Munyombwe et al. (2020)](https://dx.doi.org/10.1136/heartjnl-2019-315510) | 9566 | UK | EQ5D-3L |  |  | ✓ | ✓ |  |  |  |  |  |  | 2 |  |  |
| [Nafees et al. (2014)](https://dx.doi.org/10.1016/j.jval.2014.08.1462) | 100 | UK | Directly elicited (TTO) |  |  |  | ✓ |  | ✓ |  |  |  |  | 2 |  |  |
| [Nam et al. (2015)](http://dx.doi.org/10.1186/s12962-015-0045-9) | 174 | UK | EQ5D-3L |  |  | ✓ | ✓ |  |  |  |  |  |  | 2 |  |  |
| [Phan et al. (2019)](http://dx.doi.org/10.1161/STROKEAHA.118.024437) | 4228 | UK | EQ5D-3L |  |  |  |  | ✓ | ✓ |  |  |  |  | 2 |  |  |
| [Pockett et al. (2018)](https://dx.doi.org/10.1080/13696998.2018.1454453) | 2103 | UK | EQ5D-3L |  | ✓ | ✓ | ✓ |  |  |  |  |  |  | 3 | ✓ |  |
| [Robertson & (2016)](https://dx.doi.org/10.1016/j.cjca.2015.05.010) | 35 | UK | SF-36 |  |  |  |  |  |  |  |  |  | ✓ | 1 |  |  |
| [Roffe et al. (2018)](https://dx.doi.org/10.3310/hta22140) | 2407 | UK | EQ5D-3L |  |  |  |  | ✓ | ✓ |  |  |  |  | 2 |  |  |
| [Sandercock et al. (2013)](https://dx.doi.org/10.1016/S1474-4422(13)70130-3) | 1520 | UK | EQ5D-3L |  |  |  |  | ✓ | ✓ |  |  |  |  | 2 |  |  |
| [Shawo et al. (2020)](http://dx.doi.org/10.3310/hta24240) | 259 | UK | EQ5D-5L |  |  |  |  | ✓ | ✓ |  |  |  |  | 2 |  |  |
| [Squire et al. (2017)](http://dx.doi.org/10.5837/bjc.2017.007) | 191 | UK | EQ5D-5L |  |  |  |  |  |  |  |  | ✓ |  | 1 |  |  |
| [Walker et al. (2021)](https://dx.doi.org/10.1136/heartjnl-2020-316990) | 1202 | UK | EQ5D-3L | ✓ |  |  |  |  |  |  |  |  |  | 1 |  |  |
| [Wallace et al. (2020)](https://dx.doi.org/10.1177/1545968319887682) | 19 | UK | EQ5D-3L |  |  |  |  | ✓ | ✓ |  |  |  |  | 2 |  |  |
| [Warraich et al. (2018)](https://dx.doi.org/10.1161/CIRCOUTCOMES.117.004528) | 9319 | UK | EQ-VAS |  |  | ✓ | ✓ |  |  |  |  |  |  | 2 |  |  |
| [Whynes (2013)](https://dx.doi.org/10.1186/1477-7525-11-155) | 655 | UK | EQ-VAS |  |  |  |  | ✓ |  |  |  |  |  | 1 |  |  |
| MI – myocardial infarction, TIA – transient ischemic attack, PAD – peripheral arterial disease, T2DM – type 2 diabetes mellitus, CKD – chronic kidney disease | | | | | | | | | | | | | | | | |

Appendix 6- Tables of characteristics for studies using EQ5D to assess (1) UK populations and (2) populations similar to the UK

| **UK populations** | | | |
| --- | --- | --- | --- |
| **Title CV health state design** | **Risk of Bias (RoB)**  **Applicability** | **Response details (e.g. participated/invited, utility assessed/participants)** | **Setting, inclusion- and exclusion- criteria** |
| Agus et al. (2016) Stable angina  RCT | Low RoB Partially applicable- exclusion criteria potentially compromise applicability | Seven [/250 withdrew] in cardiac CT arm; five [/250] in EST arm) MI for missing data in sensitivity analysis | 2 Rapid Access Chest Pain Clinics within one healthcare trust in Northern Ireland, October 2010-October 2013.  Inclusion: People with symptoms of recent onset stable chest pain, defined as troponin negative without symptoms suggestive of unstable angina, and no known established coronary artery disease. Exclusion: a history of contrast media reaction, previous known coronary disease, a body mass index greater than 35, tachyarrhythmias, impaired renal function with an estimated GFR of less than 35 ml/min, and standard reasons not to treadmill . |
| Ali et al. (2017) stroke Cohort | Potentially serious RoB - 21.8% proxy responses Partially applicable- UK value set applied to international health state descriptions | Complete data on EQ5D-3L and mRS were available for 0.78=3858/4946 | Virtual International stroke Trials Archive (VISTA) dataset, which pools data from registries and trials across 36 countries, 2008-the time of analysis.  Inclusion: stroke survivors with complete mRS and EQ5D-3L scores documented at 3 months.  Exclusion: acute registers containing fewer than 100 records and sources which did not require standard diagnostic criteria for stroke to be met. |
| Alva et al. (2014) MI + post-MI + stroke + Heart Failure RCT | Low RoB Directly applicable | Of the 352 participants recruited, 48 did not have a blood test (due to failed venepuncture) so were excluded from the final analysis. | General practitioners in the catchment areas of the 23 participating UKPDS hospitals, 1977-1991. Inclusion: People with diabetes aged 25-65 who had a fasting plasma glucose greater than 6 mmol/L on two mornings, 1-3 weeks apart.  Exclusion: complicating history of cardiovascular or other chronic diseases, clear indications for alternative treatments, and contraindications to the diabetes treatments assessed. |
| Ankolekar et al. (2014) stroke RCT | Low RoB Partially applicable- international | Between July 2001 and July 2011, 2450 patients were enrolled into ENOS from 18 countries. Of 2141 patients who had completed day 90 follow-up, cognition data were available for 1572 patients, including 246 who died. | 18 countries from 7 geographic regions (Africa, America, South Asia, Southeast Asia, Australasia, Europe, and United Kingdom), July 2001-July 2011.  Inclusion: Previously independent stroke survivors within 48 hours of symptom onset who had high systolic blood pressure (140-220 mm Hg) and limb weakness.  Exclusion: definite treatment indications or contraindications to nitrates and/or restrike antihypertensive therapy, as well as confounding diseases including coma, sensory stroke, neurological or psychiatric disease, liver or renal dysfunction. |
| Babber et al. (2020) PAD cohort | Potentially serious RoB - 56% response and no multiple imputation Partially applicable- IC, non-diabetic a subgroup | Seventy-five patients from the vascular outpatient clinic were screened, of whom 42 eligible patients were recruited to start the trial | The vascular outpatient clinic at Charing Cross Hospital, London, UK, December 2014-September 2015.  Inclusion: Non-diabetic people with IC of the legs diagnosed using the Edinburgh Claudication Questionnaire and no tissue loss.  Exclusion: Ankle brachial pressure index of 0·90 or higher, inability to comply with the study protocol, implanted electrical or defibrillator device, or recent leg injury. |
| Briggs et al. (2017) MI+stroke RCT | Low RoB Partially applicable- international | 16480 EQ5D measurements in 16488 participants (99.95%) | 788 sites worldwide, May 2010-December 2011.  Inclusion: People with a history of documented type 2 diabetes mellitus, a glycated hemoglobin level of 6.5% to 12.0%, and either a history of established cardiovascular disease or multiple risk factors for vascular disease.  Exclusion: recent or concurrent incretin-based therapy, end-stage renal disease and long-term dialysis, recipients of renal transplantation, serum creatinine level higher than 6.0 mg per deciliter (530 μmol per liter). |
| Ezeofor et al. (2021) PAD pilot trial | Potentially serious RoB - excluding by diabetes and shoes size may introduce bias, source not reported Partially applicable- supplemented by Dutch data | None reported | Setting not reported. Inclusion: Adults with PAD and critical limb ischemia after successful lower limb revascularisation who had European shoes size <46. Exclusion: not reported. |
| Ford et al. (2018) Stable angina RCT | Low RoB Directly applicable | 151 (83%) of 181 patients with no obstructive CAD were randomized | Elective, adult referrals to 2 large regional hospitals (Golden Jubilee National Hospital and Hairmyres Hospital) providing invasive cardiac services to all patients in the West of Scotland (population 2.5 million), November 2016-November 2017.  Inclusion: People undergoing clinically indicated, elective diagnostic coronary angiography for the investigation of definite or probable angina.  Exclusion: non-coronary indication for invasive angiography (e.g., valve disease) and inability to give informed consent' (i.e. those without angina excluded). |
| Forster et al. (2015) stroke RCT | Potentially serious RoB - partial proxy response Directly applicable | Response rates for patient reported outcomes at 6 months were 75.2% (300/399) in control and 77.3% (310/401) in intervention and at 12 months, 67.2% (268/399) in the control and 70.1% (281/401) in the intervention group. MI for missing data | Cluster-randomised stroke care co-ordinators based in the community, July 2009-March 2011. Inclusion: People who had experienced a stroke in the previous 6 weeks and were awaiting a stroke care co-ordinator.  Exclusion: a nursing or care home residence and main requirement for palliative care. |
| Gallagher et al. (2019) Heart failure cohort | Low RoB Directly applicable | The EQ5D-3L questionnaire had the highest completion rate, with all questions being answered by 140 patients (92%) | 2 specialist cardiology clinics at the Royal Brompton Hospital London, May 2015-May 2017. Inclusion: People with heart failure attending the clinics.  Exclusion: not reported. |
| Green et al. (2018) PAD RCT | Potentially serious RoB - 30/81 screened declined participation, potentially introducing selection bias Partially applicable- IC | Of the 81 screened, 30 were eligible and consented to participate. Patients were excluded either because they chose not to participate (n = 31) or because they did not meet the inclusion criteria (n = 20). | A single, tertiary, vascular surgical unit in a university teaching hospital, May 2015-July 2019. Inclusion: People with PAD, symptomatic unilateral calf claudication and post-exertional ankle-brachial pressure index (ankle brachial pressure index) <0.9.  Exclusion: warfarin therapy, diagnosed malignancy, a unilateral thigh IC or bilateral IC in any location. |
| Hurdus et al. (2020) Stroke Cohort | Potentially serious RoB - multiple imputation used but low response rate Directly applicable | 181/5557 (3.3%) withdrew, excluded 510 (9.2%) due to failed data linkage and 296 (5.3%) without diagnosis, leaving 4570. Multiple imputation used for missing data | 48 NHS hospitals in England, November 2011-September 2013.  Inclusion: Adults hospitalised with all types of acute myocardial infarction.  Exclusion: terminal illness or other factors preventing follow-up. |
| Jenkinson et al. (2013) Stroke Validation | Potentially serious RoB - volunteer bias Directly applicable | Questionnaires were returned by 151 respondents (36.11%) | 19 socio-demographically diverse general practices, 12 in London and 7 in North West England Inclusion: stroke survivors identified using Read codes.  Exclusion: severe illness or mental incapacity. |
| Lewis et al. (2014) MI RCT | Potentially serious RoB - people with EQ5D assessment reported healthier than those in wider VALIANT study Partially applicable- international | 2556/14703 in wider VALIANT study included in EQ5D sub-study | Sites in 10 countries (Argentina, Australia, Canada, Denmark, France, Germany, Italy, Sweden, the United Kingdom, and the United States), December 1998-June 2001.  Inclusion: Adults with an acute MI occurring between 12 hours and 10 days before randomization and clinical evidence of acute heart failure, radiological evidence of heart failure, or left ventricular ejection fraction (LVEF) 35% as assessed by echocardiogram or left ventriculogram or LVEF <40% assessed by radionuclide scan.'.  Exclusion: not reported. |
| Logan et al. (2014) Post-stroke RCT | Low RoB Directly applicable | 0.996 (from RCT methods) Overall, 11,126 patient invitations were sent, with 1448 (13%) interested people replying. 852 (8%) baseline visits arranged. 568 invited people (5.1%) were eligible and randomised. | GPs, primary care therapy teams, community stroke teams or outpatient clinics at 15 sites throughout England, Scotland and Wales, November 2009-August 2011.  Inclusion: Adults who had experienced a stroke > 6 weeks previously  Exclusion: inability to comply with the protocol, and participation in a therapy programme or active rehabilitation. |
| Luengo-Fernandez et al. (2013) Stroke, TIA, Pop. Controls cohort | Low RoB Directly applicable | 748 strokes + 440 TIAs enrolled… EQ5D information was available for 759 (70%), 723 (75%), and 479 (67%) patients alive at the 1-,12-, and 60-month follow-up, respectively. | 9 general practice registers in Oxfordshire, ranging in deprivation levels and across rural/urban areas, April 2002-the time of analysis.  Inclusion: People who had experienced a suspected stroke or transient ischemic attack, ascertained using various methods,  Exclusion: temporary registration at a participating practice. |
| McCreanor et al. (2021) Stable angina RCT | Low RoB Directly applicable | 200 randomised out of the 230 enrolled (87.96%)  195 EQ5D assessments across 200 participants (97.5%) | 5 study sites in the UK: Imperial College Healthcare NHS Trust, Essex Cardiothoracic Centre, Royal Bournemouth and Christchurch Hospitals NHS Trust, East Sussex Healthcare NHS Trust, and Royal Devon and Exeter NHS Trust, January 2014-August 2017.  Inclusion: Adults aged under 85 years with angina or equivalent symptoms and at least one angio-graphically significant lesion (≥70%) in a single vessel that was clinically appropriate for PCI. Exclusion: complicating cardiovascular diseases or clinical history including acute coronary syndrome, previous coronary artery bypass graft surgery, contraindications to drug-eluting stents, moderate-to-severe pulmonary hyper tension, life expectancy less than 2 years. |
| Mejia et al. (2014) Heart Failure RCT | Low RoB Directly applicable | 13/260 enrolled missing EQ5d at baseline, MI used | Heart failure services, acute wards and GPs in Birmingham and Darlington, 2006-2008.  Inclusion: People with a record of heart failure from either hospital discharge following an acute episode or their GP register.  Exclusion: cognitive disabilities, care home residency, life-threatening diseases and inability to give informed consent. |
| Monahan et al. (2017) Heart Failure cohort | Low RoB Partially applicable- population have symptoms of heart failure not confirmed diagnosis | 397 patients were eligible for inclusion; 45 were excluded. Of the 352 participants recruited, 48 were excluded from the final analysis. The remaining 304 participants formed the validation cohort. | 28 primary care practices in central England, May 2011-August 2013.  Inclusion: People aged 55 years or over presenting with symptoms suggestive of heart failure.  Exclusion: previous confirmed diagnosis (that is, with objective evidence) of heart failure or other recent acute coronary syndrome, an obvious alternative diagnosis and severe symptoms requiring immediate management. |
| Munyombwe et al. (2020) MI cohort | Low RoB Directly applicable | EQ5D response rates: 97.5% (9332/9566), 74.7% (6679/8945), 63.9% (5572/8719) and 62.7% (5047/8043) at hospitalisation, 1month, 6 months and 12 months, respectively | People admitted to 77 National Health Service hospitals in England with an acute coronary syndrome, November 2011-June 2015.  Inclusion: Adults experiencing myocardial infarction.  Exclusion: terminal stage of illness and other circumstances making follow-up unsuitable. |
| Nam et al. (2015) MI RCT | Low RoB Partially applicable- NSTEMI | 350/853 (41%) enrolled to primary study randomised. ‘EQ-5D-3L responses were missing in 17 and 24 % of the trial population at 6- and 12-months, respectively.’ | 6 UK hospitals, October 2011-May 2013.  Inclusion: People with a clinical diagnosis of recent NSTEMI and at least one risk factor for coronary artery disease (e.g. diabetes mellitus).  Exclusion: complicating clinical history including CABG, confirmed ischaemic or coronary disease and haemodynamic instability, as well as definite indications or contraindications to treatment and life expectancy of less than a year. |
| Phan et al. (2019) stroke Cross-sectional | Low RoB Directly applicable- international but UK reported separately | n/a dataset; MI for missing data | 4 high-quality population-based incidence studies from Australasia and Europe, 1996-2013. Inclusion: All people experience a first stroke.  Exclusion: studies which did not adhere to reporting standards for stroke incidence studies. |
| Pockett et al. (2018) UA+MI cohort | Low RoB Directly applicable | 1350 responded out of 2179 identified as eligible (61.96%) | 3 UK hospitals (in Barnet, Cardiff and Peterborough), January 2021-May 2021.  Inclusion: Adults discharged within the previous month following admission for a myocardial infarction or unstable angina.  Exclusion: recent revascularization or coronary artery bypass graft and type 1 diabetes mellitus. . |
| Roffe et al. (2018) Stroke RCT | Potentially serious RoBs- partial proxy response Directly applicable | Fully informed consent was given by 6991 (87%) of patients [/8003] | 136 hospitals (secondary care) across England, Northern Ireland and Wales with acute stroke wards, April 2008-June 2013.  Inclusion: stroke survivors who could be treated within 24 hours of hospital admission and 48 hours of stroke onset.  Exclusion: no definite indications for or contraindications to oxygen or a life-threatening disease other than stroke. |
| Sandercock (2013) Post-stroke RCT | Low RoB Partially applicable- international , potentially unrepresentative as 95% participants excluded due to clear indication for treatment | Response rates conditional on the patients being alive varied from 90% to 74% | Sites across Australia, Austria, Belgium, Canada, Italy, Mexico, Norway, Poland, Sweden, and UK, May 2000-July 2011.  Inclusion: stroke survivors with known time of onset for whom treatment was promising but unproven and could be started within 6 hours.  Exclusion: previous CT or MRI of intracranial haemorrhage and structural brain lesions that could mimic stroke (e.g. cerebral tumour). |
| Shawo et al. (2020) Stroke RCT | Potentially serious RoB- mapping used Partially applicable- early supported discharge group potentially unrepresentative | 573/674. Reasons for withdrawal: choice of patients [49/101 (49%)] and study centre staff [20/101 (20%)] to discontinue involvement.' Multiple imputation used for missing data. | 19 NHS study centres, November 2012-July 2015.  Inclusion: Adult stroke survivors receiving early supported discharge.  Exclusion: ability to participate in a rehabilitation programme that focused on extended activities of daily living. |
| Squire et al. (2017) Post-heart failure cross-sectional | Low RoB Directly applicable | 185 EQ5D assessments across 191 participants (96.86%) | 7 centres in England, January 2015-May 2015.  Inclusion: Adults diagnosed with chronic heart failure (New York Heart Association [NYHA] class II–IV) in the previous 12 months.  Exclusion: inability to understand English, and concurrent participation in any clinical trial for heart failure or treatment for an episode of acute decompensated heart failure. |
| Walker et al. (2021) Stable angina RCT | Low RoB Directly applicable | 1202 patients (55% of [2205] eligible) were recruited and allocated to NICE guidelines–directed care | 6 UK hospitals (in Leeds, Glasgow, Leicester, Bristol, Oxford and central London), November 2012-March 2015.  Inclusion: People aged 30 years or older who had suspected stable angina, pre-test likelihood of coronary heart disease of 10-90% and were considered suitable for coronary revascularisation.  Exclusion: being clinically unstable, a previous acute coronary event or revascularisation, and having recently received or being contraindicated for imaging. |
| Wallace et al. (2020) Stroke RCT | Low RoB Partial applicability- spasticity potentially an unrepresentative subgroup | 355 assessed for eligibility, 27/28 received allocated intervention. MI for missing data | Focal spasticity clinics at the National Hospital for Neurology and Neurosurgery, London, 2009-2014.  Inclusion: stroke survivors with functional impairment from focal finger or wrist spasticity who could participate and conceivably benefit from treatment.  Exclusion: contraindications to or recent use of onabotulinumtoxin A, fixed contracture in the upper limb, additional neurological impairment not related to stroke, uncontrolled upper-limb pain, cognitive impairment preventing informed consent and the ability to follow task instructions.. |

| **Populations comparable to the UK (if required)** | | | |
| --- | --- | --- | --- |
| **Title CV health state design** | **Risk of Bias (RoB)**  **Applicability** | **Response details (e.g. participated/invited, utilty assessed/participants)** | **Setting, inclusion- and exclusion- criteria** |
| Vaidya et al. (2019) PAD Case series | Low RoB  Partially applicable – Dutch population taken to be comparable to UK. | ‘Out of 250 patients included, 204 (81.6%) returned the survey instrument containing the SF-36 and EQ-5D.’ | Three hospitals (Atrium Medical Center Heerlen, Orbis Medical Centre Sittard and the Maastricht University Medical Centre) in Limburg, the Netherlands, Jan 2009-Nov 2013.  Inclusion: PAD diagnosed as in-hospital performed ankle brachial index of ≤0.9  Exclusion: use of medication known to affect coagulation (e.g., cumarins, direct factor Xa-inhibitors and factor II-inhibitors, heparin), known coagulation disorders, age under 18 years and the presence of active malignancy or chronic inflammatory diseases. |
